# WEE1 kinase inhibition to overcome acquired resistance to targeted therapies in colorectal cancer

**DOI:** 10.1038/s44321-026-00434-4

**Published:** 2026-05-19

**Authors:** Kristi Buzo, Laura Bizzozero, Marilena Lentini, Alena Opattova, Beatriz Hernández-Suárez, Michael Torres, Giulia Chiabotto, Clelia Nisticò, Giada De Lazzari, Eugenia R Zanella, Gaia Grasso, Elisa Mariella, Erika Durinikova, Michael Linnebacher, Andrea Sartore-Bianchi, Salvatore Siena, Livio Trusolino, Alberto Bardelli, Sabrina Arena

**Affiliations:** 1 Department of Oncology, University of Torino, Torino, Italy; 2 Candiolo Cancer Institute, FPO - IRCCS, Candiolo, TO Italy; 3 IFOM ETS – The AIRC Institute of Molecular Oncology, Milan, Italy; 4 Molecular Oncology and Immunotherapy, Clinic of General Surgery, University Medical Center Rostock, 18057 Rostock, Germany; 5 Department of Oncology and Hemato-Oncology, Università degli Studi di Milano (La Statale), Milan, Italy; 6 Niguarda Cancer Center, Department of Hematology, Oncology and Molecular Medicine, Grande Ospedale Metropolitano Niguarda, Milan, Italy

**Keywords:** Cancer, Digestive System, DNA Replication, Recombination & Repair

## Abstract

Molecular therapies targeting the EGFR/MAPK pathway have improved outcomes in colorectal cancer (CRC), yet acquired resistance remains a major clinical challenge. Oncogenic signaling can activate stress response pathways that sustain tumor survival under therapeutic pressure. Among these, the DNA damage response (DDR) maintains genomic integrity and may represent a targetable vulnerability in resistant tumors. To investigate this, we developed a preclinical platform of CRC models with acquired resistance to anti-EGFR agents (“ARes platform”). A targeted pharmacological screen of DDR inhibitors identified WEE1 kinase as a leading therapeutic candidate. Validation in xenograft models and patient-derived organoids confirmed that anti-EGFR-resistant CRCs retained, and in some cases increased, sensitivity to WEE1 inhibition. Mechanistically, resistant cells exhibited elevated DNA damage, heightened replication stress, and accelerated mitotic entry, culminating in cell death upon WEE1 blockade. These findings establish WEE1 as a promising therapeutic target in CRC with acquired resistance to EGFR inhibition and support the clinical evaluation of WEE1 inhibitors, alone or combined with DNA-damaging agents, for patients progressing on anti-EGFR–based therapies.

## Synopsis

Acquired resistance to anti-EGFR therapies in colorectal cancer induces a replication stress-driven vulnerability that can be therapeutically exploited through WEE1 inhibition.

- CRC models with acquired resistance to anti-EGFR-based therapies exhibit elevated basal replication stress, nucleotide pool imbalance and persistent γH2AX accumulation, consistent with ongoing DNA damage signaling.
- Pharmacologic inhibition of WEE1 exacerbates replication stress, abrogates cell cycle checkpoint control and induces catastrophic DNA damage in resistant derivatives.
- WEE1 inhibition markedly impairs RAD51-mediated homologous recombination (HR), generating a functional HR-deficient-like state despite baseline HR proficiency, thereby creating an actionable therapeutic vulnerability.
- Combination treatment with WEE1 inhibition and SN-38 demonstrates strong synergistic activity (Bliss synergy score >10), supporting replication stress-based combinatorial strategies.
- The therapeutic efficacy of WEE1 inhibition has been validated in vivo using xenograft models and patient-derived organoids progressing upon EGFR-targeted therapies.

## Abstract (web summary)

Acquired resistance to anti-EGFR therapies in colorectal cancer induces a replication stress-driven vulnerability that can be therapeutically exploited through WEE1 inhibition.

## Graphical abstract

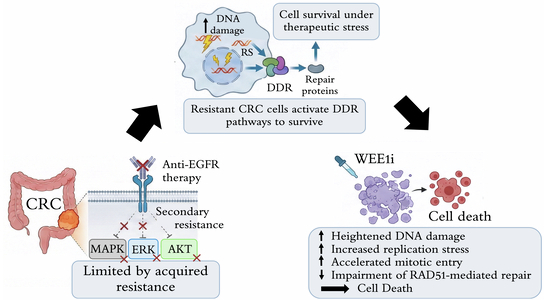

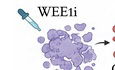

The paper explained **Problem**

Molecularly selected metastatic colorectal cancer (mCRC) patients might initially benefit from anti-EGFR-based targeted therapies, but resistance inevitably develops, leading to disease progression and limited treatment options. Although multiple genetic mechanisms of resistance have been identified, effective therapeutic strategies specifically targeting tumors that progress on EGFR blockade are lacking.

**Results**

Using a comprehensive platform of colorectal cancer models with acquired resistance, including cell lines, xenografts, and patient-derived organoids, we identified WEE1 inhibition as a consistent therapeutic vulnerability. Resistant models displayed elevated basal DNA damage, increased replication stress, and nucleotide imbalance compared to parental counterparts. Pharmacological inhibition of WEE1 induced extensive DNA damage, abrogated cell cycle checkpoints, and impaired RAD51-mediated homologous recombination repair, generating a functional repair-deficient state despite baseline homologous recombination proficiency. In vivo tumor growth suppression was observed in resistant xenografts, and efficacy was confirmed in organoids derived from tumors progressing on targeted therapy. Combination treatment with WEE1 inhibition and chemotherapy showed marked synergistic activity.

**Impact**

This study provides preclinical evidence that acquired resistance to anti-EGFR therapies in mCRC is associated with replication stress-driven vulnerabilities that can be therapeutically targeted. WEE1 inhibition represents a promising strategy for patients who have progressed on anti-EGFR-based regimens, either as monotherapy or in rational combination with irinotecan-based treatments. These findings support the clinical investigation of WEE1 inhibitors in the relapse after anti-EGFR therapy setting and highlight replication stress markers as potential tools for patient stratification.

## Introduction

Colorectal cancer (CRC) ranks as the second leading cause of cancer-related deaths and the third most diagnosed type of cancer globally (Siegel et al, 2024). Up to 2004, the only option for treating metastatic CRC (mCRC) was chemotherapy, which is associated with essential limitations such as dosage-related toxicity (Hossain et al, 2022). The development of molecular therapies targeting the EGFR/MAPK pathway over the last two decades has marked a significant advancement in the standard of care for CRC (Cervantes et al, 2023). The first approved targeted agent, cetuximab, is a monoclonal antibody (moAb) that binds and downregulates the epidermal growth factor receptor (EGFR) and, as panitumumab, it is used for treating wild-type *RAS* and *BRAF* CRC patients (Pissarra et al, 2020). Extensive research has led to the identification of small molecule inhibitors targeting other components of the EGFR/MAPK pathway. Among these, BRAF inhibitors (BRAFi), in combination with the anti-EGFR drugs, have been approved for the treatment of BRAF V600E CRC patients (Bendell et al, 2014). More recently, the approval of KRAS G12C inhibitors (KRAS^G12C^i) has marked a crucial milestone, dispelling the belief that KRAS alterations are not targetable and paving the way for exploring new target molecules against specific mutant *RAS* genes (Amodio et al, 2020; Di Nicolantonio et al, 2021). The use of these targeted molecules has improved the clinical course of mCRC patients both in terms of progression-free survival (PFS) and overall survival (OS). However, this response is often only transient, as acquired resistance inevitably emerges in most patients (Di Nicolantonio et al, 2021; Oddo et al, 2016; Ríos-Hoyo et al, 2024; Yaeger et al, 2023).

The search for new therapeutic options remains a critical challenge for mCRC patients that have developed resistance to targeted agents. Given that genomic instability and replication stress (RS) are key characteristics of CRC (Burrell et al, 2013), targeting the DNA damage response (DDR) might represent a valuable therapeutic strategy. Alterations in *KRAS, BRAF*, and *EGFR*, which are known to drive secondary resistance in CRC (Arena et al, 2015; Arena et al, 2016; Misale et al, 2014b; Misale et al, 2012), can trigger an abnormal increase in cyclin-dependent kinases (CDK) activity, heightened DNA replication origin firing and RS (Kotsantis et al, 2018). Under physiological circumstances, the level of RS is tightly regulated by ATR/CHK1 and WEE1 kinases, which counteract the activation of CDK and ensure a proper rate of DNA replication during the S-phase progression. The loss of ATR, CHK1 or WEE1 in a high RS context can result in excessive exposure to single-strand DNA (ssDNA), followed by extensive DNA breakage and cell death (Thangaretnam et al, 2025). Based on this rationale, many clinical trials are currently testing the efficacy of DDR inhibitors (DDRi) in different solid tumors, including CRC. WEE1 inhibitors (WEE1i) are gathering particular attention considering the crucial role of WEE1 in ensuring proper repair before undergoing cell division (Thangaretnam et al, 2025). Indeed, recently, response to WEE1 inhibition in CRC has been found to correlate with the mutational status of *TP53* tumor suppressor gene and *RAS* gene (Seligmann et al, 2021). Our previous research has brought forth a novel perspective for the treatment of CRC by leveraging the use of DDRi as a novel therapeutic opportunity (Mauri et al, 2020). For the first time, we have provided evidence of the efficacy of several DDRi, including ATRi, CHK1i, WEE1i, ATMi, DNA-PKi and PARPi (Arena et al, 2020; Durinikova et al, 2022). We conducted these studies exploiting an extensive panel of CRC models, including cell lines, organoids and mouse xenografts, recapitulating the molecular CRC landscape. Our findings revealed that approximately 30% of the models, including those carrying *KRAS* and *BRAF* mutations, showed sensitivity to at least one of the DDRi tested as monotherapy (Durinikova et al, 2022).

Despite all the promising evidence on the potential benefit of WEE1 inhibition and possibly other DDRi in CRC, to our knowledge no studies have so far evaluated DDRi efficacy in the setting of acquired resistance to EGFR-blockade in CRC. We hypothesize that chronic treatment with targeted drugs might increase RS and DNA damage, potentially developing a novel Achilles’ heel to overcome secondary resistance to therapies. To demonstrate this, we leverage unique 2D and 3D, in vitro and in vivo CRC models of acquired resistance to drugs targeting the EGFR/MAPK pathway and test the efficacy of several DDRi, leading to WEE1 as the most compelling target. Further analysis has shed light on mechanisms of response that hopefully could lead to the identification of putative biomarkers for improving patient stratification and to the design of novel effective therapeutic approaches for mCRC treatment.

## Results

### Generation and pharmacological testing of models with acquired resistance to anti-EGFR targeted therapies

**Figure 1:**
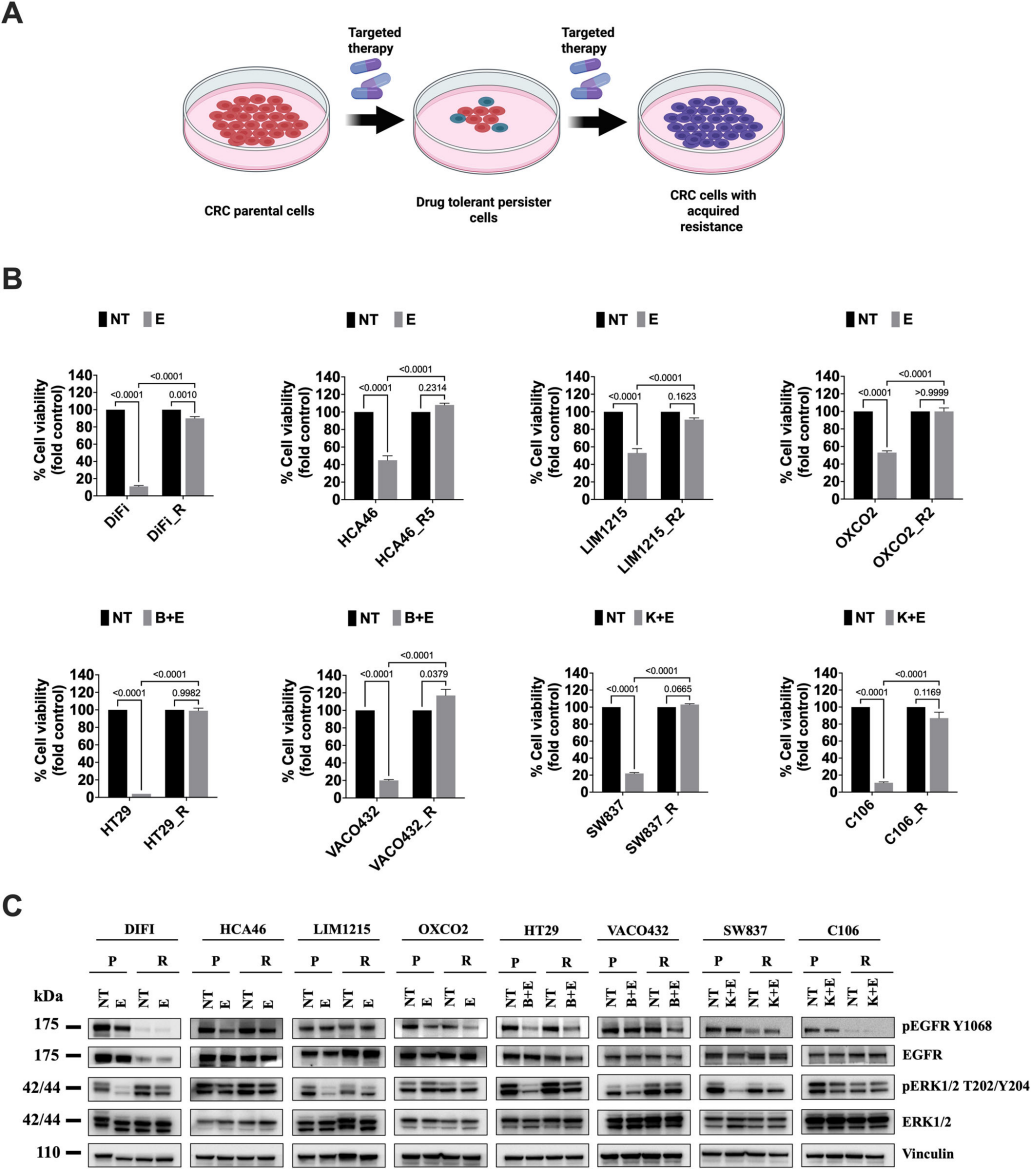
Generation and characterization of colorectal cancer models with acquired resistance to anti-EGFR therapies. (**A**) Schematic overview of the experimental strategy used to generate cell line models with acquired resistance to targeted therapies. (**B**) Cell viability of parental and resistant CRC models seeded on day 0 and treated from day 1 for 6 days with the indicated targeted therapies. Cell viability was measured using CellTiter-Glo. Cells were seeded at the following densities (cells per well): DiFi (1 × 10⁴), HCA46 (1.2 × 10⁴), LIM1215 (4 × 10³), OXCO2 (1 × 10⁴), HT29 (3 × 10³), VACO432 (6 × 10³), C106 (1.2 × 10⁴), SW837 (1 × 10⁴). Treatments: E: EGFRi-cetuximab (25 µg/ml) for HCA46, OXCO2, LIM1215 and DiFi; B + E: BRAFi-dabrafenib (4 µM) plus EGFRi-cetuximab (5 µg/ml) for HT29; B + E: BRAFi-dabrafenib (2 µM) plus EGFRi-cetuximab (5 µg/ml) for VACO432; K + E: KRASG12Ci (AMG 510, 3 µM) plus EGFRi-cetuximab (15 µg/ml) for SW837; K + E: KRASG12Ci (AMG 510, 0.5 µM) plus EGFRi-cetuximab (15 µg/ml) for C106. Data are presented as mean + SEM from *n* = 3 biological replicates each performed in technical triplicate. Statistical significance was assessed using two-way ANOVA with Tukey’s multiple-comparisons test. (**C**) Immunoblot analysis of parental (P) and resistant (R) cells treated for 24 h with the same drug concentrations as in (**B**). Vinculin was used as loading control. Images are representative of *n* = 3 independent biological replicates. Source data are available online for this figure.

**Figure EV1:**
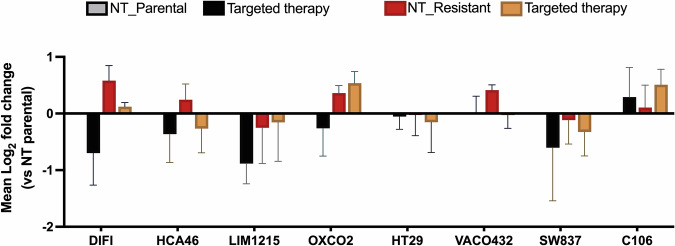
Biochemical assessment of MAPK activity by phospho-ERK quantification in HT29 and HCA46 pairs post-treatment with targeted therapy. Quantification of pERK levels normalized to total ERK and Vinculin. Data are presented as mean ± SEM from *n* = 3 independent biological experiments.

Due to the critical lack of models derived from patients who developed resistance during treatment, we have taken advantage of a unique platform of in vitro cell models with acquired resistance to the anti-EGFR moAb cetuximab (Acquired Resistance – ARes platform), partly previously established in our laboratory. These include two microsatellites stable (MSS) cell lines, DiFi and HCA46, and two microsatellites instable (MSI) cell lines, OXCO2 and LIM1215 (Arena et al, 2015; Misale et al, 2014a; Misale et al, 2012) (Table EV1 and Fig. 1A). In light of the approval of BRAFi and, more recently, KRAS-G12Ci molecules, we have expanded our platform, including additional models that reflect the latest advancements in CRC treatment. Specifically, we generated two new models with acquired resistance to a combination of BRAFi (dabrafenib) and anti-EGFR (cetuximab) drugs by chronically treating the parental HT29 (MSS) and VACO432 (MSI) BRAF mutant cell lines. Additionally, following the same procedure, we developed other two new MSS models resistant to a combination of KRAS-G12Ci (AMG 510) and anti-EGFR (cetuximab), derived from the KRAS-G12C mutant C106 and SW837 cell lines (Table EV1). The acquisition of resistance was confirmed through viability assay after pharmacological treatment (Fig. 1B). Importantly, biochemical analysis demonstrated the sustained activation of ERK signaling in all resistant models, regardless of the drugs used to generate the resistant derivatives (Figs. 1C and EV1).

**Figure 2:**
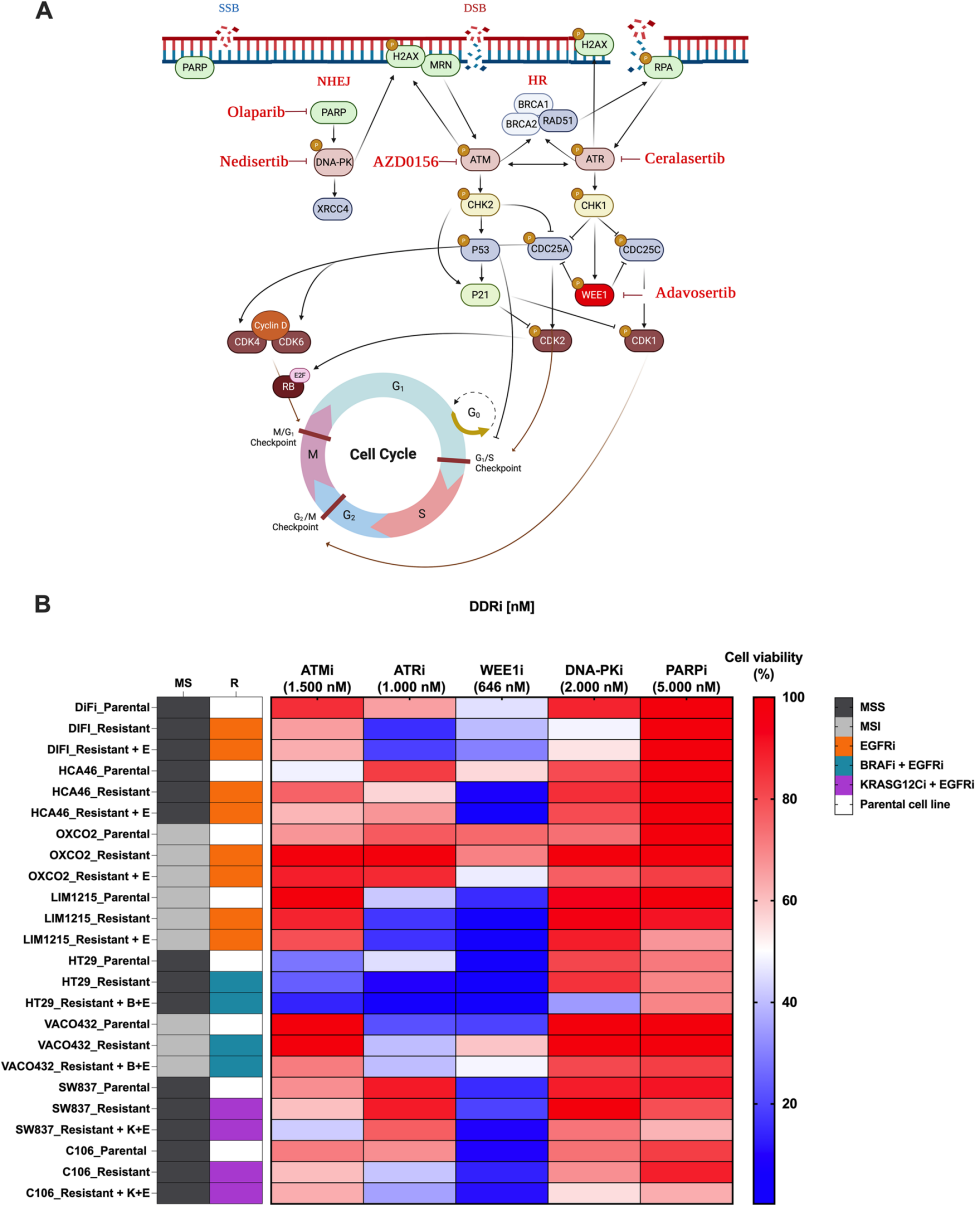
Replication stress inhibitors effectively overcome acquired resistance in colorectal cancer models. (**A**) Schematic overview of the DNA damage response (DDR) pathway and inhibitors investigated in this study. (**B**) Heatmap showing sensitivity to DDR inhibitors at clinically relevant concentrations in parental and resistant CRC cell line pairs determined by 6-day viability assays. Parental and resistant cells were seeded on day 0 at the following densities (cells per well): DiFi (1 × 10⁴), HCA46 (1.2 × 10⁴), LIM1215 (4 × 10³), OXCO2 (1 × 10⁴), HT29 (3 × 10³), VACO432 (6 × 10³), C106 (1.2 × 10⁴), SW837 (1 × 10⁴). The next day increased concentrations of DDR inhibitors were added either alone or, for the resistant cells, in combination with the targeted therapy: cetuximab (EGFRi, 25 µg/ml) for resistant HCA46, OXCO2, LIM1215 and DiFi; dabrafenib (BRAFi; 4 µM) plus cetuximab (5 µg/ml) for resistant HT29; dabrafenib (2 µM) plus cetuximab (5 µg/ml) for resistant VACO432; AMG510 (KRAS-G12Ci, 3 µM) plus cetuximab (15 µg/ml) for resistant SW837; AMG 510 (0.5 µM) plus cetuximab (15 µg/ml) for resistant C106. In the heatmap, only clinically relevant concentrations are shown in Fig. EV1: AZD0156 (ATMi, 1.5 µM), ceralasertib (ATRi, 1 µM), adavosertib (WEE1i, 646 nM), nedisertib (DNA-PKi, 2 µM), and olaparib (PARPi, 5 µM), while complete drug–response curves are shown in Fig. EV2. Heatmap values represent the mean of *n* = 3 independent biological experiments performed with technical triplicates. Microsatellite status and drugs used in the resistance setting are indicated. Source data are available online for this figure.

**Figure EV2:**
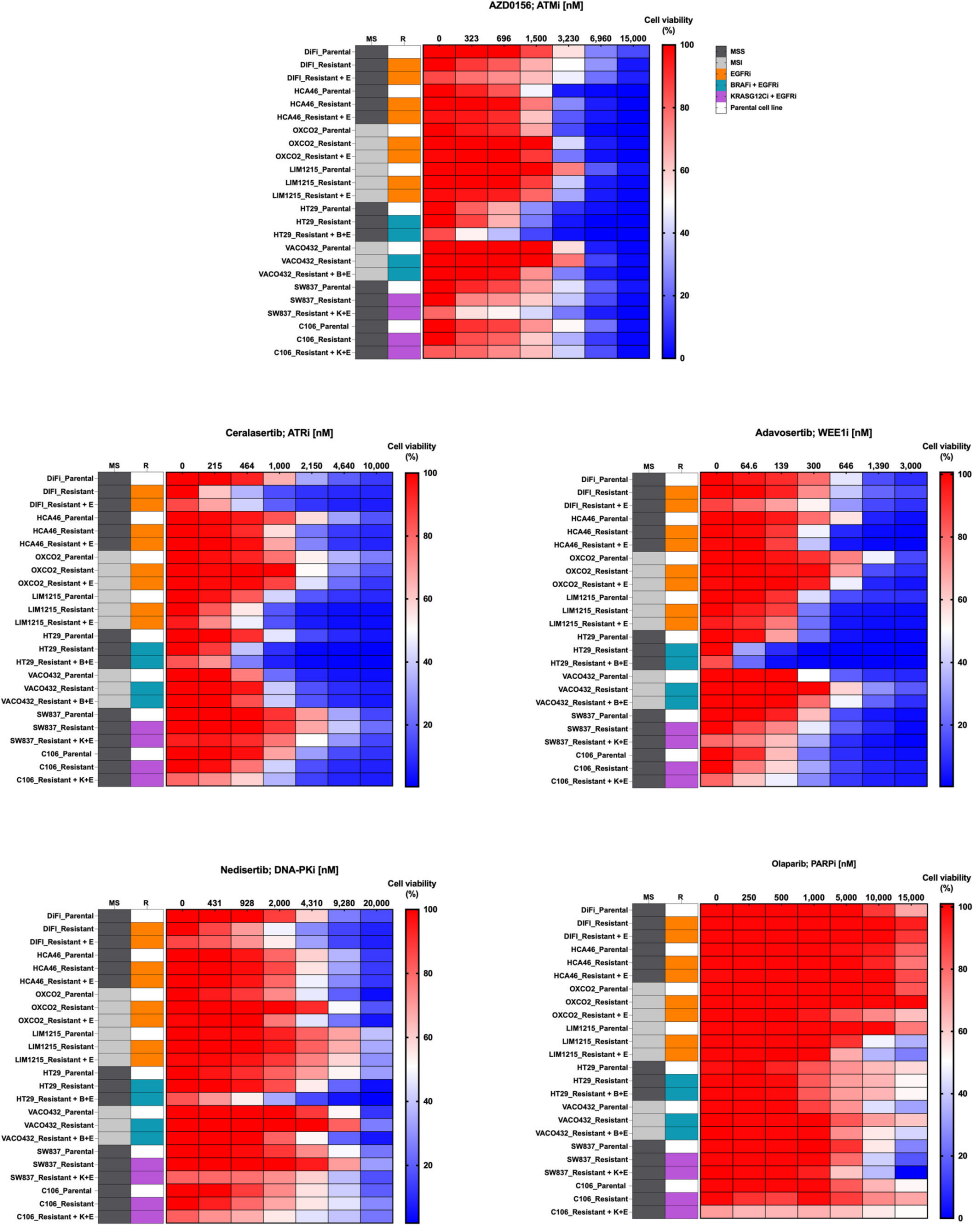
Pharmacological screening of models with acquired resistance to EGFR-based targeted treatments. Heatmaps depict sensitivity to five different DNA damage response inhibitors (DDRi) across eight CRC cell line pairs with acquired resistance to distinct molecularly targeted therapies. Drug sensitivity was assessed using a 7-day viability assay using CellTiter-Glo® Assay. Parental cell lines were seeded at the following densities (cells per well): DiFi (1 × 10⁴), HCA46 (1.2 × 10⁴), LIM1215 (4 × 10³), OXCO2 (1 × 10⁴), HT29 (3 × 10³), VACO432 (6 × 10³), C106 (1.2 × 10⁴), SW837 (1 × 10⁴). 24 h after seeding cells were treated with DDRi as monotherapy at the indicated range of concentrations: AZD0156 (ATMi, 0–15 µM), ceralasertib (ATRi, 0–10 µM), adavosertib (WEE1i, 0–3 µM), nedisertib (DNA-PKi, 0–20 µM), and olaparib (PARPi, 0–15 µM), whereas cell lines with acquired resistance were treated either with DDRi monotherapy (same range) or in combination with the molecularly targeted therapy to which resistance had developed: E: EGFRi-cetuximab (25 µg/ml) for HCA46, OXCO2, LIM1215 and DiFi; B + E: BRAFi-dabrafenib (4 µM) plus EGFRi-cetuximab (5 µg/ml) for HT29; B + E: BRAFi-dabrafenib (2 µM) plus EGFRi-cetuximab (5 µg/ml) for VACO432; K + E: KRAS-G12Ci (AMG 510, 3 µM) plus EGFRi-cetuximab (15 µg/ml) for SW837; K + E: KRAS-G12Ci (AMG 510, 0.5 µM) plus EGFRi-cetuximab (15 µg/ml) for C106. For each cell line, three independent biological replicates were performed, each with technical triplicates. The first column indicates microsatellite (MS) status: microsatellite stable (MSS, dark gray) or microsatellite instable (MSI, gray). The second column indicates the targeted therapy to which resistance was acquired: EGFRi-cetuximab (E, orange), BRAFi-dabrafenib plus EGFRi-cetuximab (B + E, ochre), or KRAS-G12Ci-(AMG 510) plus EGFRi-cetuximab (K + E, purple). Heatmaps were generated using GraphPad Prism software.

To determine whether acquired resistance to anti-EGFR–based therapies reshapes reliance on specific DDR pathways, we tested a selected panel of DDR inhibitors (PARPi, DNA-PKi, ATMi, ATRi, and WEE1i) within our ARes platform (Arena et al, 2020) (Fig. 2A). DDRi were used either as single agents or, in the case of resistant cell derivatives, also in combination with targeted agents to identify any potential additive or synergistic effects (Figs. 2B and EV2).

Inhibitors of the RS pathway proved to be the most effective ones and, among them, the WEE1i (adavosertib) emerged as the most potent one (Fig. 2B). When considering the complete drug response curve (Fig. EV2), WEE1i demonstrated greater efficacy in most of the resistant models compared to their parental counterparts. Regarding the combination of targeted treatment with DDRi, no higher efficacy was observed respect to the use of DDRi as single agents (Figs. 2B and EV2).

### WEE1, a druggable target in the CRC secondary resistance setting

**Figure 3:**
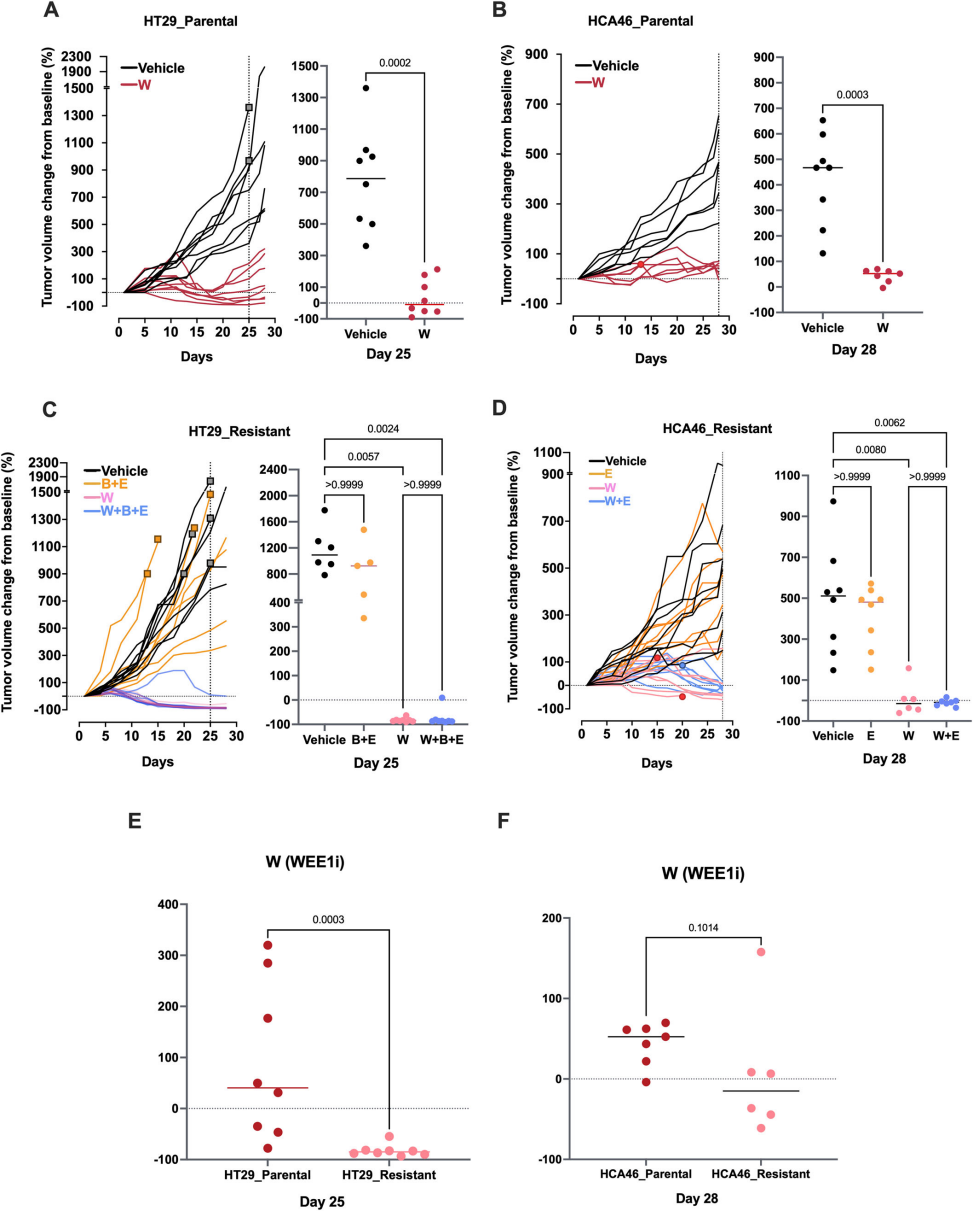
WEE1 inhibition overcomes resistance to targeted therapies in CRC xenograft models. HT29 and HCA46 parental and resistant cells (1:1 mixture with Matrigel) were injected subcutaneously into female NOD-SCID mice (5–9 weeks of age). Tumors were measured three times weekly, and once tumors reached 100–150 mm³ mice were randomized in 6 groups (*n* = 8 mice per group). Specifically, for the parental models, vehicle and WEE1i, while for the resistant ones: Vehicle, WEE1i, targeted therapy alone (EGFRi for HCA46; BRAFi+EGFRi for HT29), or combination therapy (WEE1i+EGFRi for HCA46; WEE1i+BRAFi+EGFRi for HT29). Treatment dosing and schedule was as follows: For WEE1i-adavosertib (60 mg/kg, twice a day (BID) by oral gavage), EGFRi-cetuximab (10 mg/kg, twice a week (BIW) intraperitoneally), BRAFi-dabrafenib (15 mg/kg, once a day (QD) by oral gavage). Animals were euthanised at ethical endpoint (1200–1500 mm³) or upon >20% body-weight loss. (**A**,** B**) Tumor growth curves in parental xenografts. (**C**,** D**) Tumor growth curves in resistant xenografts. (**E**,** F**) Endpoint tumor volume comparison between parental and resistant tumors. Statistical analysis: Mann–Whitney U test (**A**, **B**, **E**, **F**) or Kruskal–Wallis test with Dunn’s multiple-comparisons correction (**C**,** D**). Squares indicate removal due to tumor burden; circles indicate removal due to weight loss. Source data are available online for this figure.

**Figure EV3:**
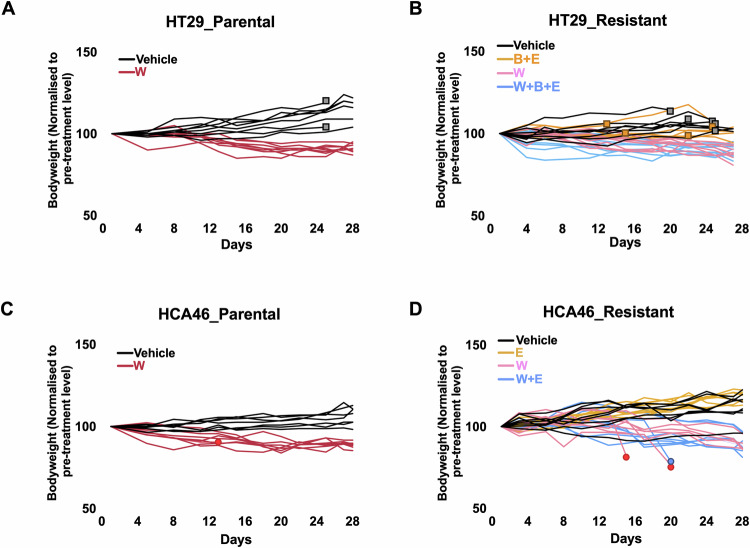
Drug tolerability in the xenograft experiment. Ten millions HT29 and HCA46 parental and resistant cells (1:1 mixture with Matrigel) were injected subcutaneously into female NOD-SCID mice (5–9 weeks old). Tumors were measured three times weekly, and once volume reached 100–150 mm³ mice were randomized and treated (*n* = 8 mice per group) twice a week (BIW) intraperitoneally with E: EGFRi-cetuximab (10 mg/kg) and/or twice a day (BID) by oral gavage for W:WEE1i-adavosertib (60 mg/kg) and once a day (QD) by oral gavage for B:BRAFi-dabrafenib (15 mg/kg,). The mean body mass was normalized to the mean value recorded prior to treatment initiation. Circles indicate mice removed from the study due to ≥20% body weight loss. (**A**,** B**) Body weight monitoring during treatment in HT29_Parental and HT29_Resistant cohorts, respectively. (**C**,** D**) Body weight monitoring during treatment in HCA46 parental and HCA46_Resistant cohorts, respectively. W: WEE1i; B: BRAFi; E: EGFRi.

**Figure EV4:**
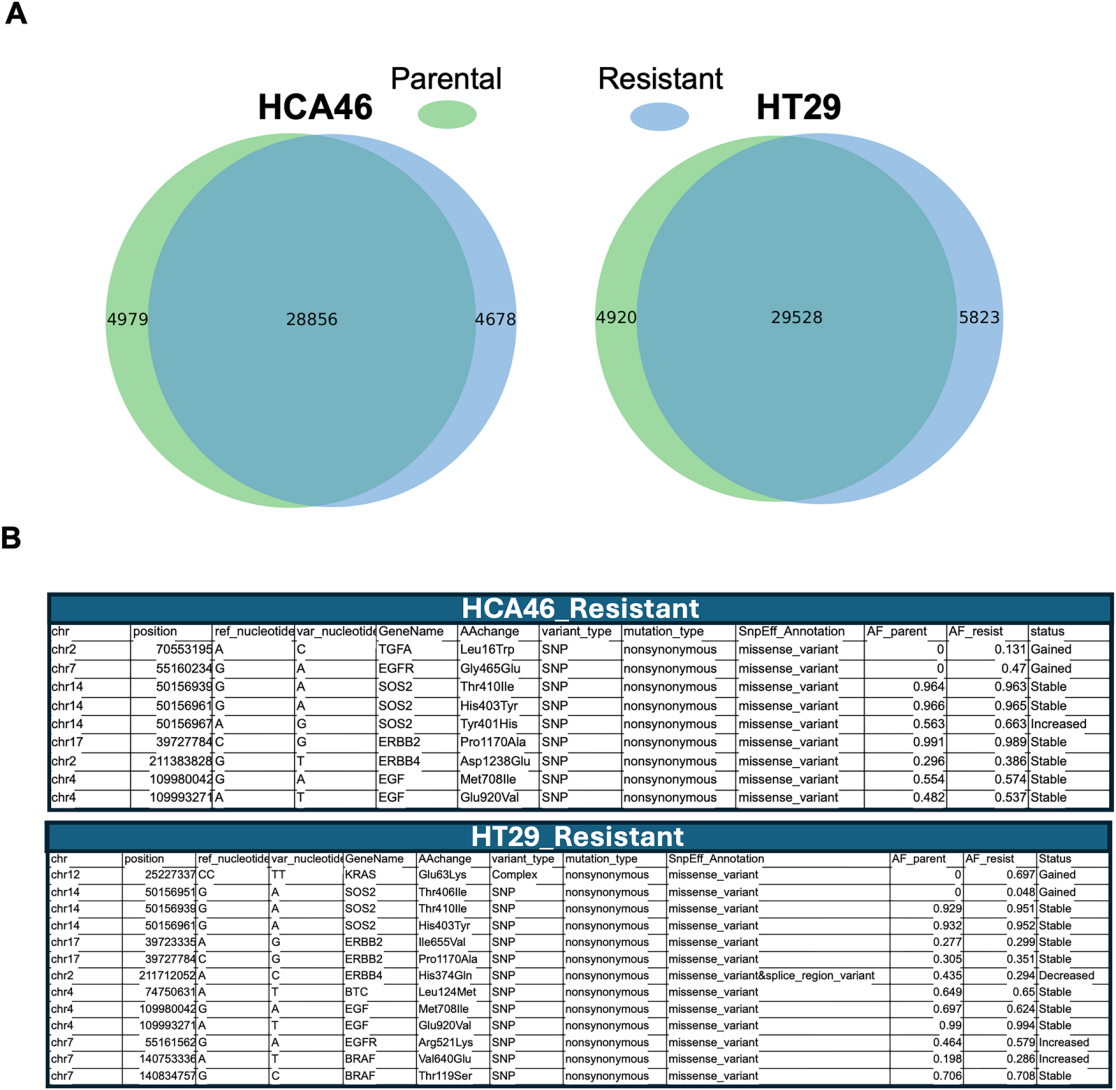
Analysis of genetic alterations upon acquisition of resistance in HT29 and HCA46 pairs. (**A**) Venn diagram illustrates the distribution of total somatic variants, depicting the number of variants shared between parental (green) and resistant (blue) cell lines as well as those unique to each. (**B**) Table of EGFR-MAPK pathway somatic nonsynonymous variants identified in resistant cell lines following whole-exome sequencing analysis. For each resistant model (HCA46 and HT29), variants are annotated for genomic position, allelic frequency, gene name and predicted functional impact.

Encouraged by our in vitro findings, we conducted a proof-of-concept in vivo study using colorectal xenograft models of acquired resistance to test the efficacy of WEE1i both as monotherapy and in combination with targeted agents. Given that MSS CRC patients represent nearly 85% of total diagnosed cases and the lack of newer therapeutic options compared to MSI patients, we focused specifically on MSS models. We selected the two most common genotypes in CRC patients eligible for targeted treatment: RAS/BRAF wild-type and BRAF-V600E mutant. Our study initially considered these two MSS cell line pairs: HCA46_Parental/HCA46_Acquired Resistant to cetuximab (EGFRi) and HT29_Parental/HT29_Acquired Resistant to the combination of dabrafenib plus cetuximab (BRAFi plus EGFRi). To mirror the in vitro experiments, the xeno-resistant models were treated with clinically relevant doses of WEE1i either as a single agent or in combination with anti-EGFR targeted therapies (Fig. 3A–F). WEE1i impaired tumor proliferation in both HT29 and HCA46 parental xenograft models (Fig. 3A,B). A more pronounced response was observed in the HT29_Resistant model, where WEE1 inhibition resulted in a near-complete suppression of tumor growth (Fig. 3C,E). In the HCA46_Resistant model, WEE1i treatment similarly reduced tumor growth (Fig. 3D), although the increase in sensitivity relative to parental tumors did not reach statistical significance (Fig. 3F), in part due to variability driven by a single outlier tumor. Consistently with our in vitro data, the combination of WEE1i with targeted agents did not show significant differences in either resistant model (Fig. 3C,D). Regarding drug tolerability, no major issues were observed concerning body weight; all treatments were well tolerated except for three isolated cases of body weight loss in the HCA46_Resistant model (Fig. EV3). Overall, the in vivo results recapitulated the in vitro findings, confirming WEE1 inhibition as a highly effective therapeutic strategy, particularly in the setting of acquired resistance. Notably, enhanced efficacy was observed in the resistant models, with the most pronounced effect in the HT29_Resistant cells (Fig. 3E,F). To exclude the possibility that the increased susceptibility to WEE1 inhibition resulted from a specific clonal selection, we performed whole-exome sequencing (WES) analyses before and after the acquisition of resistance. Comparative analysis revealed that the majority of variants detected in the parental models were retained in the corresponding resistant derivatives (Fig. EV4A). As expected, the acquisition of resistance was associated with the emergence of different and specific variants known to sustain resistance, including a KRAS mutation in the HT29_Resistant model and, consistently with previous reports (Arena et al, 2015; Arena et al, 2016), the acquisition of an EGFR extracellular domain mutations in the HCA46_Resistant model (Fig. EV4B). Collectively, these data support the notion that response to WEE1 inhibition is unlikely to be driven by clonal genetic selection, but rather reflects resistance-associated adaptations. This experimental evidence supports WEE1 inhibition as a promising therapeutic strategy for treating MSS CRC patients who develop secondary resistance to anti-EGFR therapies.

### Acquisition of resistance to EGFR blockade results in higher basal DNA damage

**Figure 4:**
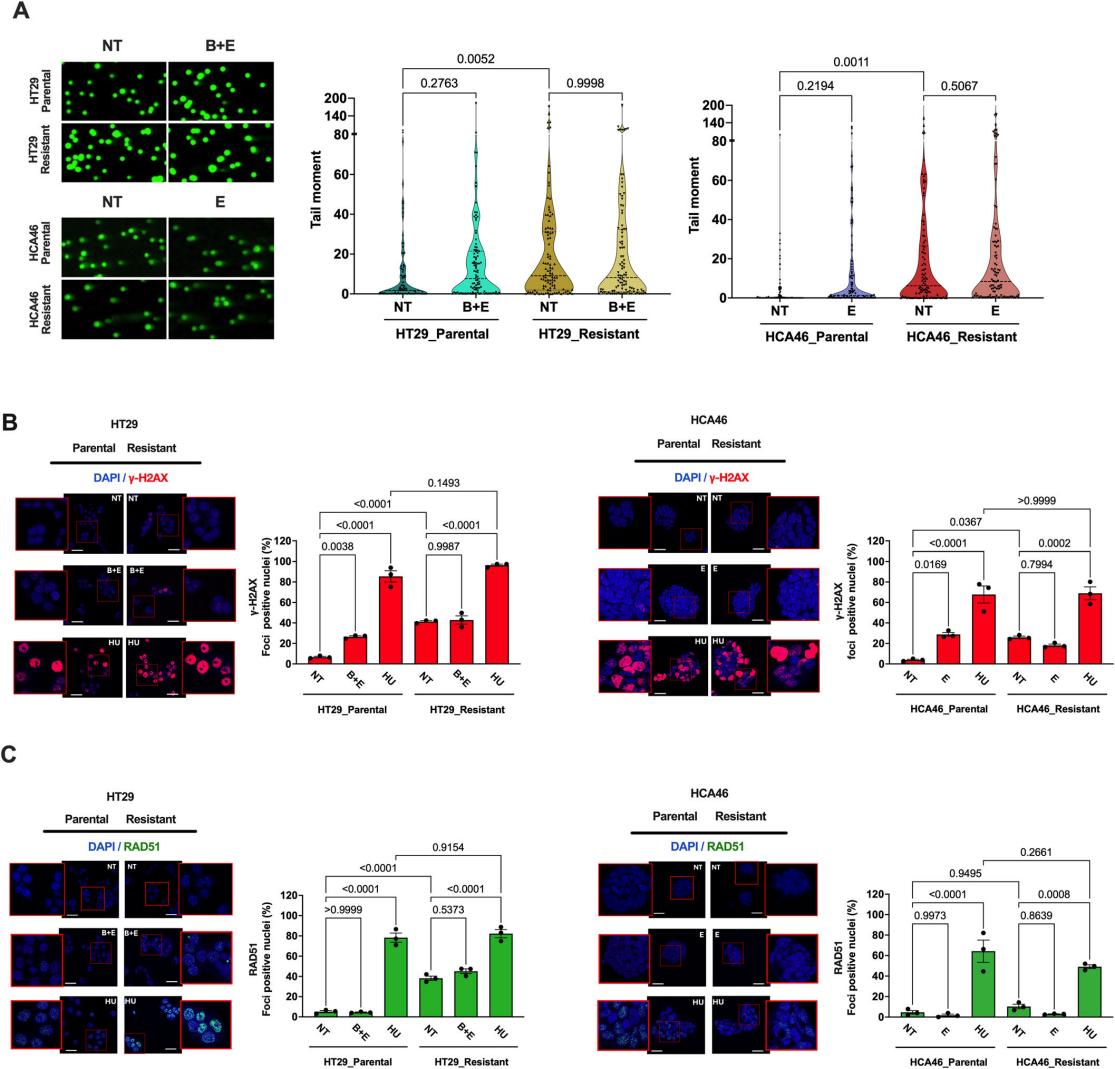
Acquired resistant models exhibit increased DNA damage at baseline. (**A**) Alkaline comet assay in HT29 and HCA46 parental and resistant cells under basal conditions or following treatment with targeted therapy (B + E): BRAFi-dabrafenib (4 µM, 24 h) plus EGFRi-cetuximab (5 µg/ml, 24 h) for HT29/E: EGFRi-cetuximab (25 µg/ml, 24 h) for HCA46. HU: hydroxyurea (2.5 mM, 24 h) was used as a positive control for replication stress and DNA damage induction. Violin plots show the tail moment distribution from 100 nuclei per condition derived from *n* = 2 independent biological experiments performed in technical duplicate. Statistical significance was assessed using Kruskal–Wallis test with Dunn’s multiple-comparisons correction. γ-H2AX (**B**) and RAD51 (**C**) immunofluorescence analysis. Cells were seeded on coverslips and treated with targeted therapy (B + E): BRAFi-dabrafenib (4 µM, 24 h) plus (EGFRi-cetuximab (5 µg/ml, 24 h) for HT29/E: EGFRi-cetuximab (25 µg/ml, 24 h) for HCA46; HU: hydroxyurea (2.5 mM, 24 h) was used as a positive control for replication stress and DNA damage induction. γ-H2AX was co-stained with RAD51, nuclei were counterstained with DRAQ-5. Data are presented as mean ± SEM from *n* = 3 independent biological experiments (at least 5 independent fields were quantified, for a total of 100 nuclei/biological experiment). Images were acquired using a Leica Stellaris confocal microscope equipped with a 63× oil-immersion objective (NA 1.4) under identical acquisition settings. Scale bar, 25 µm. Statistical significance was assessed using one-way ANOVA with Tukey’s multiple-comparisons test. Source data are available online for this figure.

Considering the increase of susceptibility to WEE1i observed in HCA46 and HT29 cells following the acquisition of resistance to EGFRi or to the combination of BRAFi and EGFRi, respectively, we sought to further elucidate the mechanisms underlying this phenomenon. We initially assessed DNA damage levels at baseline and following targeted treatment in both cell pair models. Comet assay analysis revealed elevated basal DNA damage (NT label) in the resistant models compared with their respective parental counterparts (Fig. 4A). The treatment with anti-EGFR-based targeted agents triggered increased DNA damage in the parental models; in contrast, no significant changes were observed in the acquired resistant cells after treatment (Fig. 4A).

**Figure EV5:**
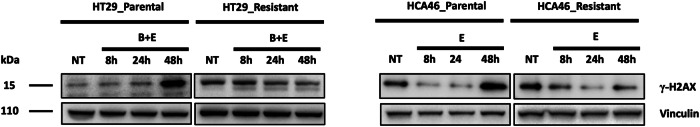
Biochemical assessment of DNA damage in HT29 and HCA46 pairs post-treatment with targeted therapy. γ-H2AX protein levels were analyzed by immunoblot as a marker of DNA damage in parental and resistant HT29 and HCA46 cell line pairs under basal conditions or after time-course treatment with targeted therapy for 8, 24, or 48 h. Cells were seeded at 5 × 10⁵ cells per well in 6-well plates on day 0 and treated the following day with E: EGFRi-cetuximab (25 µg/ml) for HCA46 or B + E: BRAFi-dabrafenib (4 µM) plus EGFRi-cetuximab (5 µg/ml) for HT29. Vinculin was used as a loading control in all panels. Images are representative of two biological replicates. Source data are available online for this figure

This observation was independently validated by biochemical analysis assessing γ-H2AX levels, a well-established marker of DNA damage. Western blot analysis of γ-H2AX protein levels in a time-course experiment confirmed increased basal DNA damage in both resistant models, as well as treatment-induced DNA damage with targeted therapy exclusively in parental cells (Fig. EV5). These results were further corroborated by immunofluorescence analysis of γ-H2AX foci formation at the basal condition (NT) and under treatment with targeted therapy or hydroxyurea (used as positive control). In line with the previous results, resistant HT29 and HCA46 cells displayed higher basal levels of γ-H2AX foci compared with their respective parental counterparts (Fig. 4B). Upon exposure to targeted therapies, parental cells showed a significant increase in γ-H2AX foci, whereas no further induction was detected in resistant cells (Fig. 4B), indicating a differential DNA damage response following treatment. We then analyzed RAD51 foci level as a marker of response to DNA damage and consistently observed that, at baseline, HT29-resistant cells exhibited higher RAD51 foci levels compared with their parental counterparts (Fig. 4C), while a similar but less pronounced trend was observed in the HCA46 cell pair. Treatment with the respective targeted agents did not induce RAD51 foci formation in either parental or resistant cells despite the cells being proficient in repair (Fig. 4C). These findings corroborate previous independent findings suggesting that anti-EGFR–based therapies may downregulate components of the HR pathway (Russo et al, 2019).

**Figure EV6:**
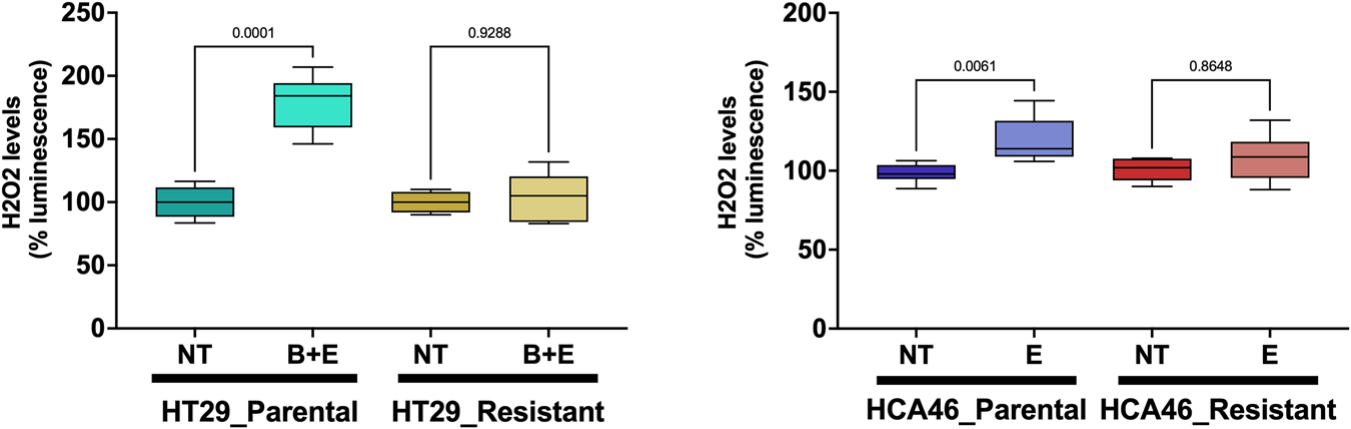
Assessment of reactive oxygen species following targeted therapy treatment. Reactive oxygen species evaluation in HT29 and HCA46 pairs in basal conditions and after treatment with targeted therapy. Cells were seeded on day 0 and treated from day 1 with targeted therapies E: EGFRi-cetuximab (25 µg/ml), while HT29 cells were treated with B + E: BRAFi-dabrafenib (4 µM) in combination with E: EGFRi-cetuximab (5 µg/ml). After 48 h, reactive oxygen species were quantified using the ROS-Glo™ H2O2 Assay. Luminescence values were normalized to untreated controls. Data are presented as mean ± SEM from *n* = 3 independent biological experiments. In the box plots the center line represents the median, the box bounds represent the 25th and 75th percentiles (interquartile range), and the whiskers extend from the minimum to the maximum value. Individual data points are overlaid. Statistical significance was assessed using a two-tailed Mann–Whitney U test.

Notably, this evidence was further supported by the parallel observation that reactive oxygen species (ROS) production increased in parental models upon exposure to anti-EGFR-based targeted agents, whereas no significant changes were observed in resistant cells following treatment (Fig. EV6), suggesting that cells with acquired resistance might be more tolerant or adapted to oxidative stress induced by chronic drug pressure.

### Emergence of secondary resistance triggers increased replication stress in CRC models

**Figure 5:**
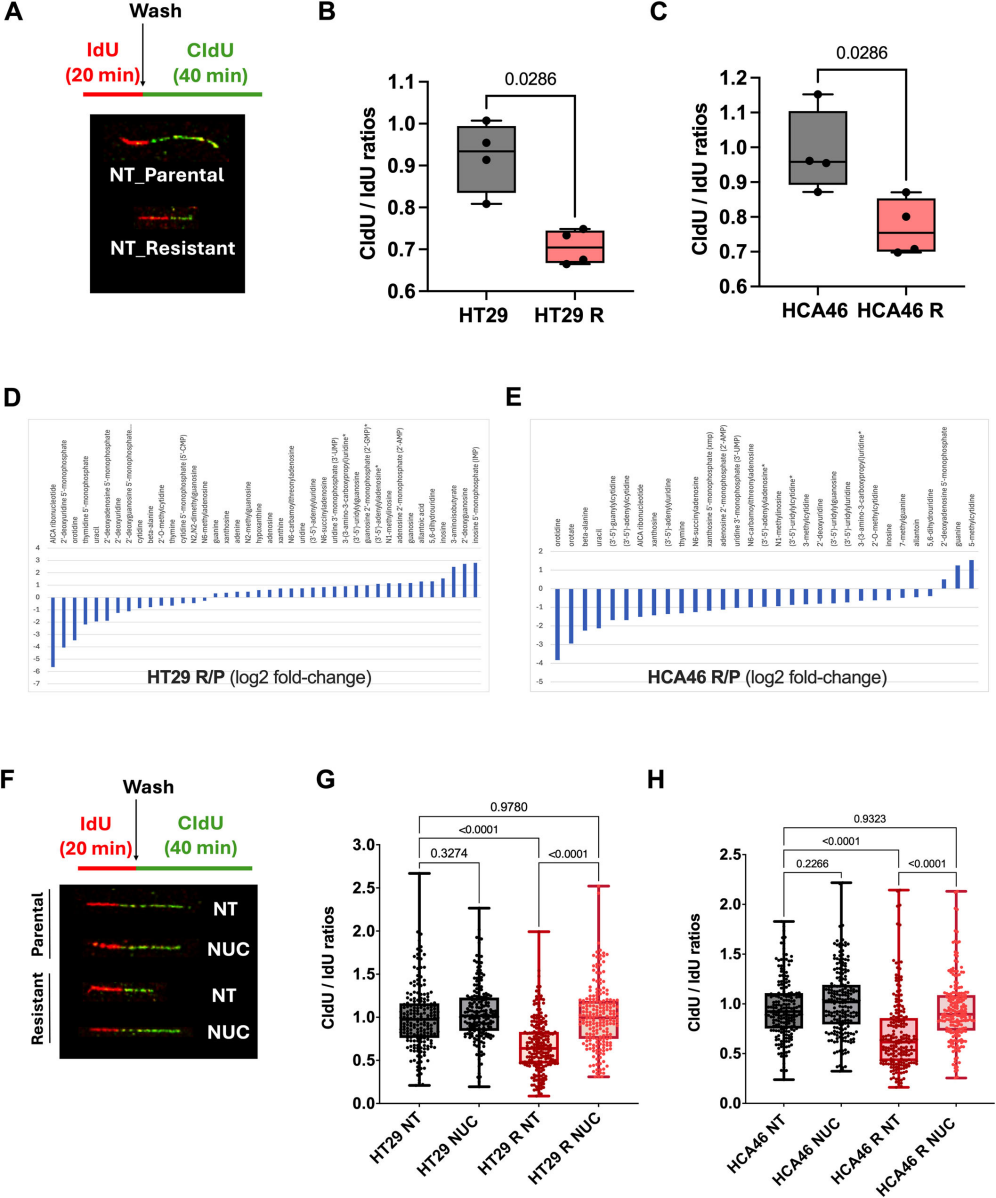
Acquired resistance to anti-EGFR therapies is associated with replication stress and nucleotide pool imbalance. (**A**) Schematic representation of the DNA fiber labeling protocol showing sequential pulsing with IdU (25 µM, 20 min) and CldU (250 µM, 40 min) and representative fibers from parental and resistant cells. (**B**,** C**) Replication fork progression in untreated parental and resistant HT29 (**B**) and HCA46 (**C**) cells measured by DNA fiber assay. Cells were seeded at 2 × 10⁵ cells per well in 6-well plates and processed 48 h later. Replication dynamics were assessed by measuring the CldU/IdU tract length ratio (with IdU values normalized to 2× to account for pulse duration differences). Data are presented as mean ± SEM from *n* = 4 independent biological replicates; ≥100 fibers were quantified per replicate (total *n* ≥ 400 fibers per condition). Statistical significance was assessed using a two-tailed Mann–Whitney U test. (**D**) Relative abundance of nucleotide metabolism intermediates in resistant versus parental HT29 and HCA46 cells (**E**) determined by untargeted metabolomics. Data are shown as log₂ fold change for significantly deregulated metabolites (*P* < 0.05). Statistical significance was assessed using a Welch two-sample t-test. (**F**) Schematic overview of the DNA fiber assay. Quantification of replication fork progression in parental and resistant HT29 (**G**) and HCA46 (**H**) cells cultured under basal conditions or supplemented with a nucleoside mixture (EmbryoMax® Nucleoside Mix, 100× stock; Merck/Millipore). Plots show the CldU/IdU tract length ratio (with IdU values normalized to 2× to account for pulse duration differences) distribution from 200 nuclei per condition derived from *n* = 2 independent biological experiments. Statistical significance was assessed using Kruskal–Wallis test with Dunn’s multiple-comparisons correction. In box plots (**B**, **C** and **G**, **H**), the center line represents the median, the box bounds represent the 25th and 75th percentiles (interquartile range), and the whiskers extend from the minimum to the maximum value. Individual data points are overlaid. Source data are available online for this figure.

To further investigate the role of RS and DNA damage, we performed a DNA fiber assay in both cell models under basal conditions, directly comparing parental and resistant counterparts (Fig. 5A–C). As expected, resistant cells exhibited a significantly lower CIdU/IdU ratio (Fig. 5B,C) indicative of impaired replication fork progression and increased replication stress associated with resistance acquisition. To corroborate these findings, we next evaluated nucleotide metabolism, a critical determinant of replication dynamics. Both resistant HT29 and HCA46 cells exhibited a pronounced imbalance in nucleotide pools between purines and pyrimidines (Fig. 5D,E). This metabolic dysregulation is consistent with the DNA fiber assay results and supports the presence of elevated basal DNA damage and replication stress in CRC cells that developed acquired resistance to anti-EGFR therapies.

**Figure EV7:**
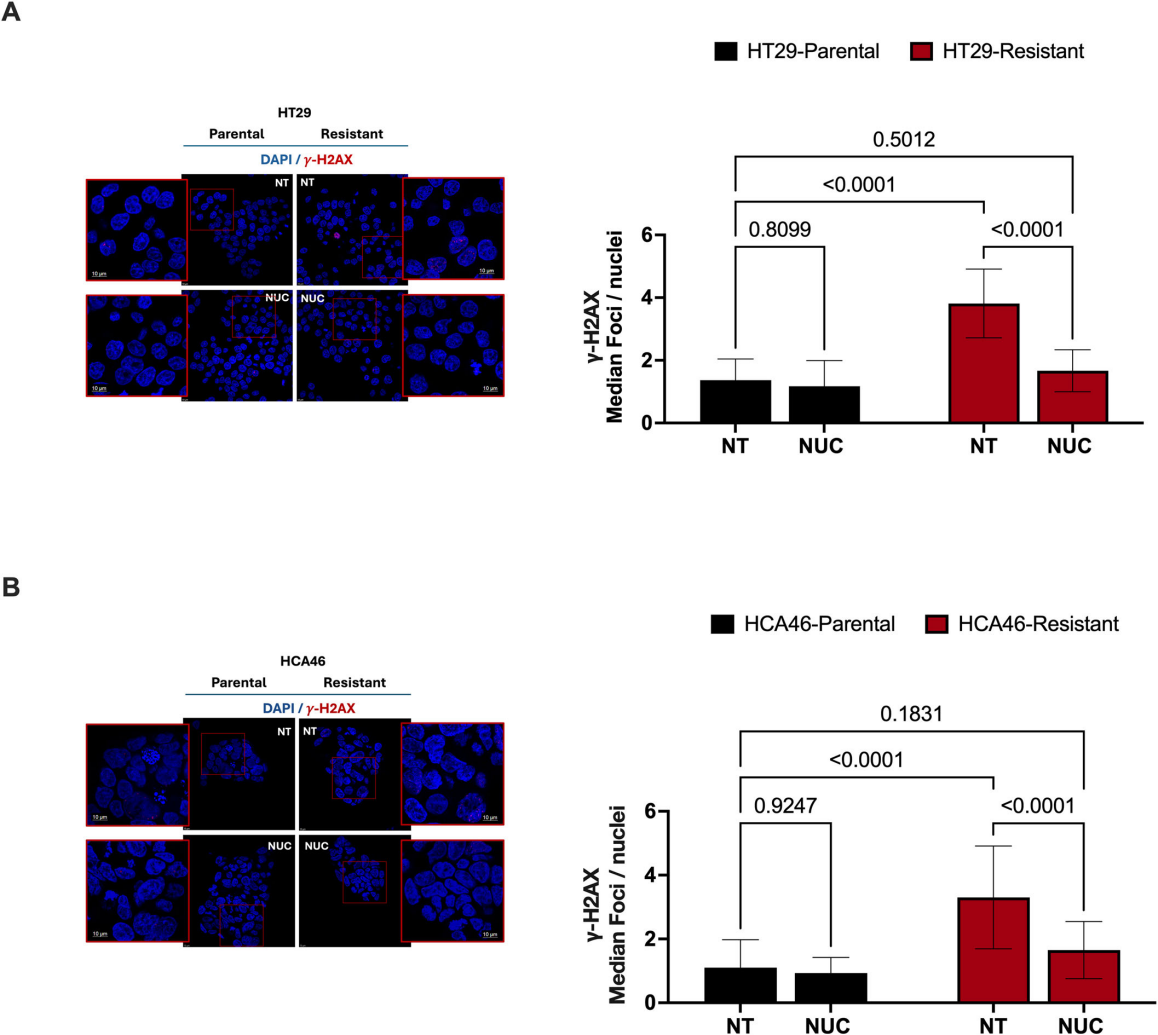
Rescue of replication of DNA damage by nucleoside supplementation in CRC cells with acquired resistance to anti-EGFR therapies. γ-H2AX immunofluorescence analysis in parental and resistant HT29 (**A**) and HCA46 (**B**) cells under basal conditions or after nucleoside supplementation (NUC). Cells were seeded on coverslips and supplemented the following day. Twenty-four hours post-supplementation, cells were fixed and processed for γ-H2AX immunostaining. Nuclei were counterstained with DAPI. Data are presented as mean ± SEM from *n* = 3 independent biological experiments (at least 5 images were quantified, counting for a total of 100 nuclei/biological experiment). Images were acquired using a Leica Stellaris confocal microscope equipped with a 63× oil-immersion objective (NA 1.4) under identical acquisition settings for all conditions. Scale bar, 10 µm. Statistical significance was assessed using one-way ANOVA with Tukey’s multiple-comparisons test.

To directly determine whether the elevated basal replication stress observed in resistant cells was driven by dysregulated nucleotide metabolism, we performed a rescue experiment using exogenous nucleoside supplementation. Resistant cells were supplemented with nucleosides (NUC) prior to DNA fiber assays (Fig. 5F–H) and γ-H2AX immunofluorescence analyses (Fig. EV7) to quantify replication dynamics and basal DNA damage, respectively. Nucleoside supplementation led to a marked reduction in replication fork stalling (Fig. 5F–H). Consistently, γ-H2AX foci were significantly decreased in resistant cells following nucleoside supplementation both in HT29 (Fig. EV7A) and HCA46_Resistant cells (Fig. EV7B). In contrast, both parental cell models did not exhibit significant changes under the same experimental conditions (Fig. EV7A,B). These findings indicate that the heightened replication stress and basal DNA damage observed in resistant cells are likely attributable to nucleotide pool imbalance and might be functionally rescued by restoring nucleotide availability.

### WEE1 inhibition triggers high levels of DNA damage and accelerated G2/M entry in tumor cells that have developed acquired resistance to EGFR blockade

**Figure 6:**
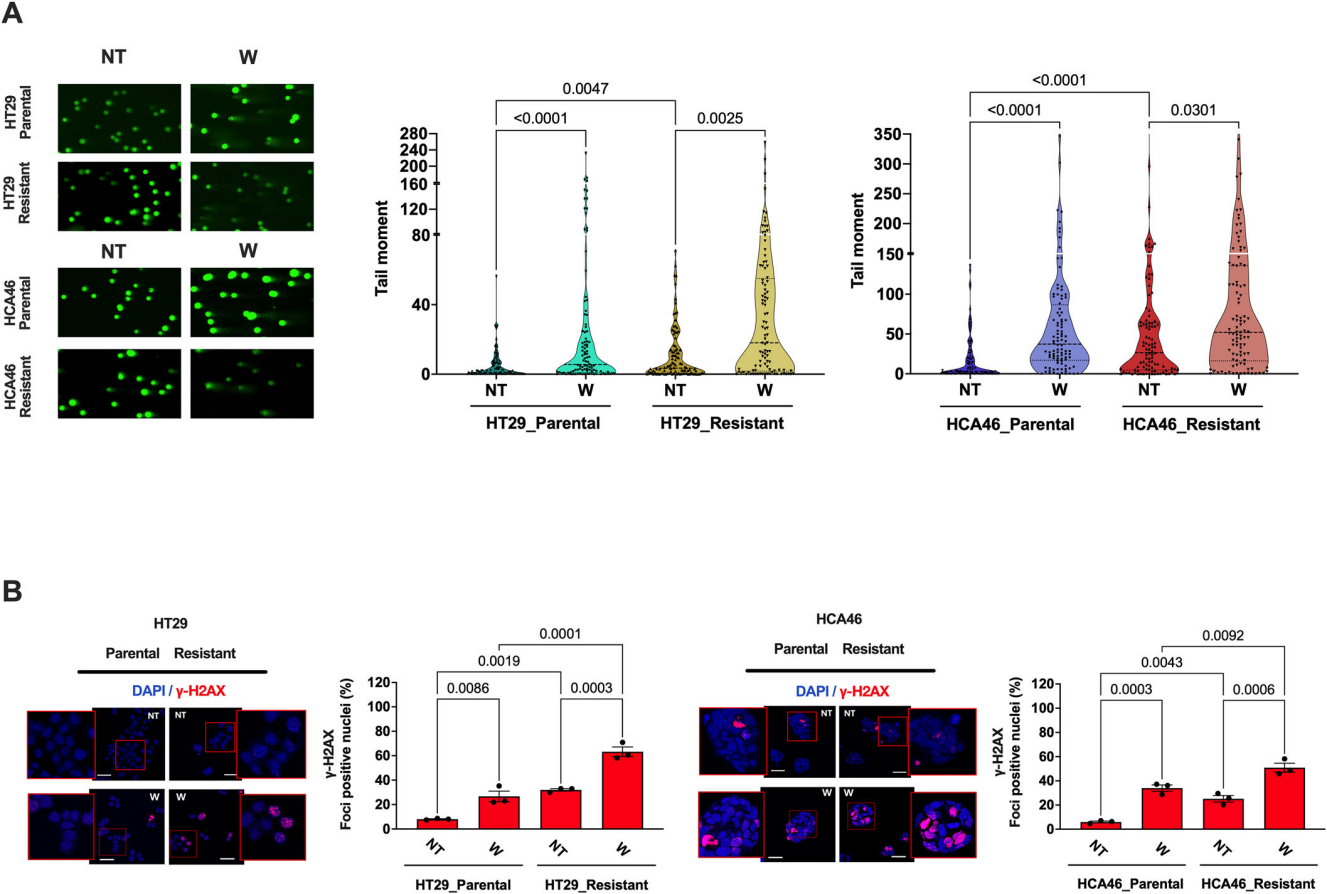
Acquired resistant models exhibit increased DNA damage at baseline and after WEE1 inhibition. (**A**) Alkaline comet assay in HT29 and HCA46 parental and resistant cells under basal conditions or following treatment with W-WEE1i-adavosertib (300 nM 8 h). Violin plots show the tail moment distribution from 100 nuclei per condition derived from *n* = 2 independent biological experiments performed in technical duplicate. Statistical significance was assessed using Kruskal–Wallis test with Dunn’s multiple-comparisons correction. (**B**) γ-H2AX immunofluorescence analysis. Cells were seeded on coverslips and treated with following treatment with W-WEE1i-adavosertib (300 nM, 8 h). Nuclei were counterstained with DRAQ-5. Data are presented as mean ± SEM from *n* = 3 independent biological experiments performed in technical duplicate (at least 5 independent fields were quantified, for a total of 100 nuclei/biological experiment). Images were acquired using a Leica Stellaris confocal microscope equipped with a 63× oil-immersion objective (NA 1.4) under identical acquisition settings. Scale bar, 25 µm. Statistical significance was assessed using one-way ANOVA with Tukey’s multiple-comparisons test. Source data are available online for this figure.

**Figure EV8:**
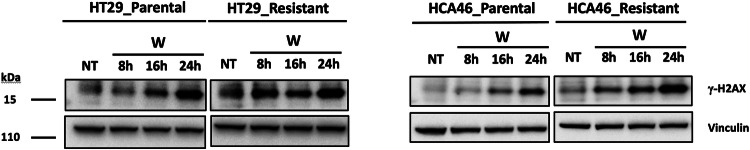
Biochemical assessment of DNA damage in HT29 and HCA46 pairs upon treatment with WEE1i. γ-H2AX protein levels were analyzed by immunoblot as a marker of DNA damage in parental and resistant HT29 and HCA46 cell line pairs under basal conditions or after time-course treatment with the WEE1 inhibitor adavosertib (300 nM) for 8, 16, or 24 h. Cells were seeded at 5 × 10⁵ cells per well in 6-well plates on day 0 and treated the following day. Vinculin was used as a loading control in all panels. Images are representative of two biological replicates.

To determine whether the differences in response to WEE1i are solely attributed to variances in basal damage and RS levels or not, we assessed these two features after WEE1 inhibition. The treatment resulted in substantial damage in both HT29 and HCA46 pairs (Fig. 6A). Notably, resistant models exhibited significantly greater DNA damage upon WEE1i exposure, as independently shown by both the comet assay (Fig. 6A), by the γ-H2AX foci formation analysis (Fig. 6B) and by a time-course western blot experiment (Fig. EV8).

**Figure EV9:**
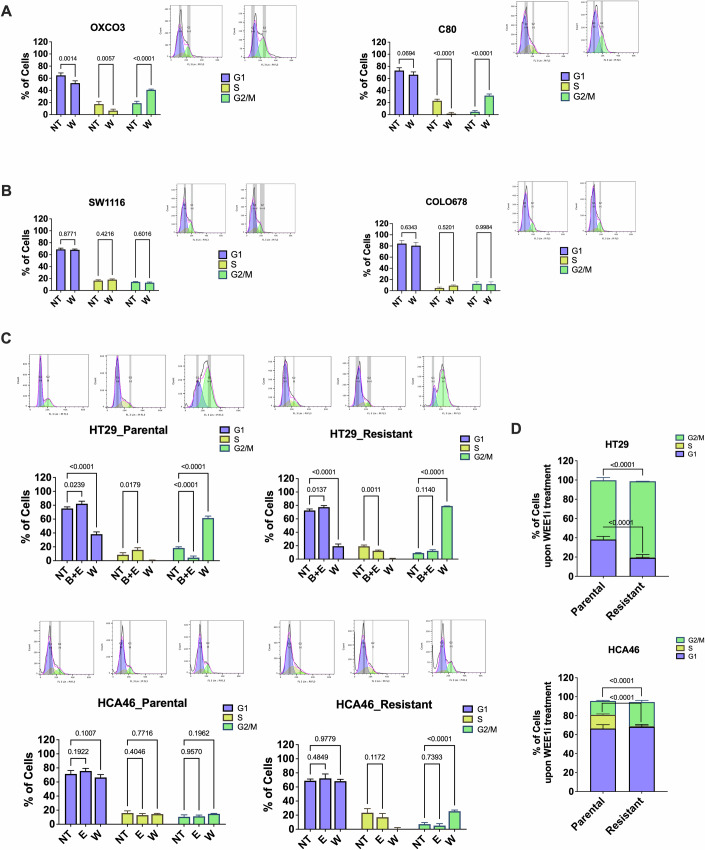
Effect of WEE1 Inhibition on the cell cycle in CRC cells. Cell cycle profiles analyzed in control models including WEE1 inhibitor-sensitive (OXCO3, C80; (**A**)) and WEE1 inhibitor-resistant (SW1116, COLO678; (**B**)) cell lines, as well as HT29 and HCA46 parental and resistant pairs (**C**,** D**). Cells were treated with adavosertib (300 nM) for 24 h. For HT29 and HCA46 pairs, additional conditions included E: EGFRi-cetuximab (25 µg/ml) for HCA46 or B + E: BRAFi-dabrafenib (4 µM) in in combination with EGFRi-cetuximab (5 µg/ml). DNA content was measured by propidium iodide staining followed by flow cytometry, and the percentage of cells in G1, S, and G2/M phases was quantified using FlowJo software. Data are presented as mean ± SEM from *n* = 3 independent biological experiments. Statistical significance was assessed using two-way ANOVA with Tukey’s multiple-comparisons test.

**Figure EV10:**
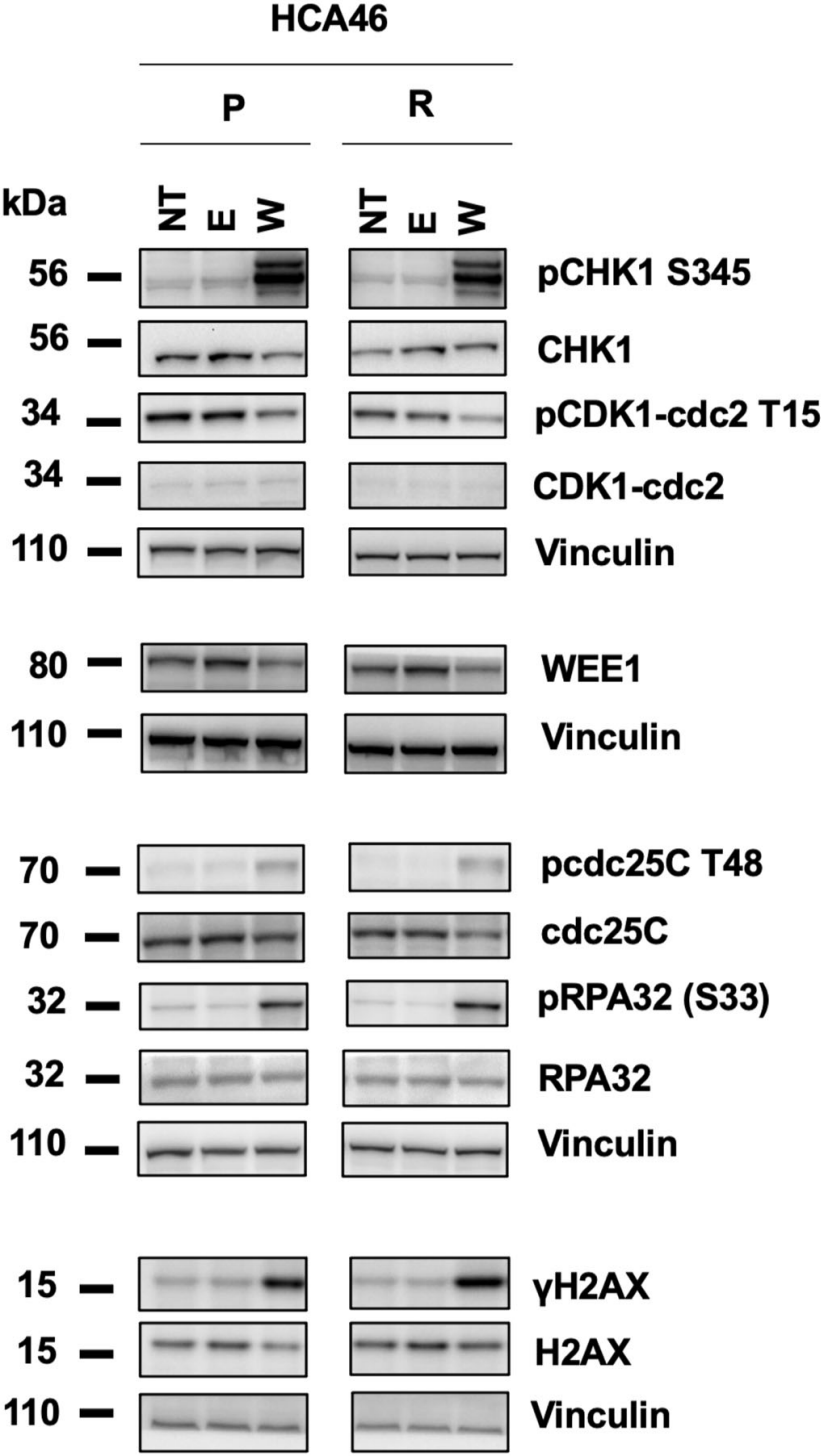
WEE1 inhibition induces replication stress and DNA damage signaling in parental and resistant HCA46 cells. Immunoblot analysis of markers of replication stress, cell cycle and DNA damage response players in in parental (P) and resistant (R) HCA46 cells seeded at 5 × 10⁵ cells per well in 6-well plates and treated after 24 h with EGFRi-cetuximab (25 µg/ml, 24 h) or W-WEE1i-adavosertib (300 nM, 24 h). Total protein levels are shown as controls, and vinculin was used as a loading control. Representative immunoblots from two independent biological experiments are shown. Source data are available online for this figure.

Given that DNA damage activates a response program promoting cell cycle arrest for ensuring proper DNA repair and considering the crucial role played by WEE1 in cell cycle checkpoint control, we performed a comprehensive cell cycle analysis. Being HT29 and HCA46 parental cells both sensitive to WEE1i, although to different extents, to strengthen the interpretation of these results, we included additional control models with known intrinsic sensitivity or resistance to WEE1i, based on our previous work (Durinikova et al, 2022). Specifically, SW1116 and COLO678 cells were used as intrinsically resistant controls, whereas OXCO3 and C80 were included as sensitive controls (Fig. EV9A,B). Consistent with their known sensitivity profile, WEE1 inhibition induced a significant accumulation of cells in the G2/M phase in sensitive models (OXCO3, C80) (Fig. EV9A), whereas no substantial changes were observed in the intrinsically resistant ones (SW1116, COLO678) (Fig. EV9B). Although both parental and resistant HT29 and HCA46 pairs were intrinsically sensitive to WEE1i, distinct cell cycle alterations were observed between parental and resistant derivatives. In HT29 cells, both parental and resistant lines displayed a shift from G1 and S phases toward G2/M following WEE1 inhibition (Fig. EV9C, upper panel). In the HCA46_Resistant model, a higher percentage of cells in the G2/M phase was observed after treatment with WEE1i (Fig. EV9D). Yet a block in G1 after WEE1i was also observed in both HCA46 parental and resistant cells. Although HCA46 cells accumulate in the G1 gate following WEE1 inhibition, this is unlikely to represent a canonical G1 arrest, as these cells are p53-null (Kramer et al, 2016). Instead, strong induction of replication-stress markers (pCHK1, pRPA and γ-H2AX) followed by activation of CDC25C (T48) and reduction of inhibitory pCDK1-Y15 (Fig. EV10) is observed and suggest premature CDK activation and entry into non-productive S-phase, resulting in replication collapse.

### WEE1 inhibition significantly hinders RAD51-mediated DNA repair and synergizes with DDRi or DNA damaging agents

In our previous study, we highlighted a functional interplay between the RS and HR pathways, as they both involve common effectors, such as RAD51 (Durinikova et al, 2022).

**Figure 7:**
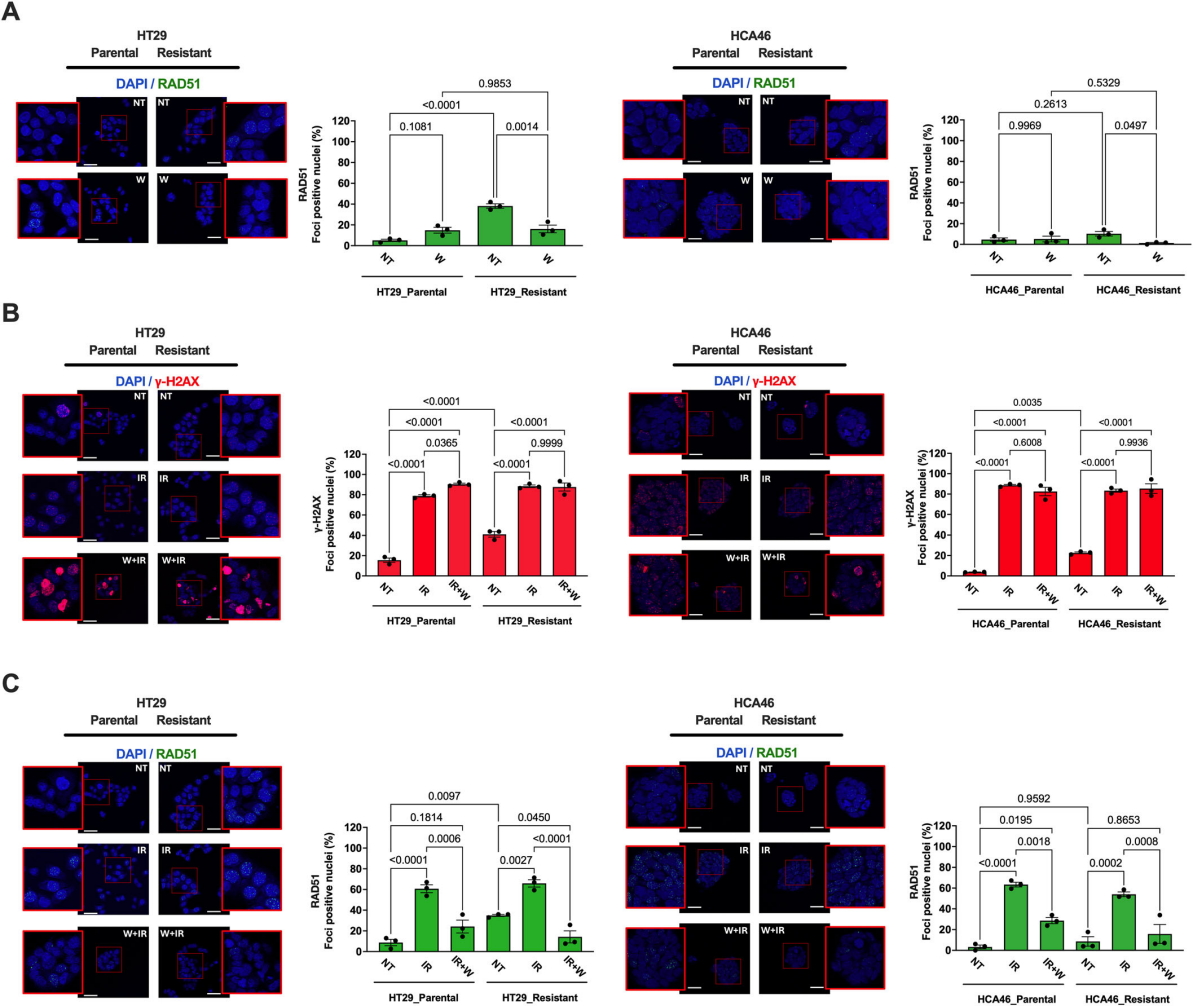
WEE1 inhibition impairs RAD51-mediated homologous recombination in CRC models. (**A**) RAD51 foci formation in parental and resistant HT29 and HCA46 cells treated with W: WEE1i-adavosertib (300 nM, 8 h). (**B**,** C**) γ-H2AX and RAD51 foci following ionizing radiation (5 Gy) with or without adavosertib pretreatment (100 nM, 48 h). Cells were fixed 4 h after irradiation. γ-H2AX was performed as a co-staining with RAD51, nuclei were counterstained with DRAQ-5. For all panels, data are presented as mean ± SEM of *n* = 3 biological experiments (at least 5 independent fields were quantified, for a total of 100 nuclei/biological experiment). Images were acquired using a Leica Stellaris confocal microscope equipped with a 63× oil-immersion objective (NA 1.4) under identical acquisition settings. Scale bar, 25 µm. Magnified images 2.5x. Statistical significance was assessed using one-way ANOVA with Tukey’s multiple-comparisons test. Source data are available online for this figure.

**Figure 8:**
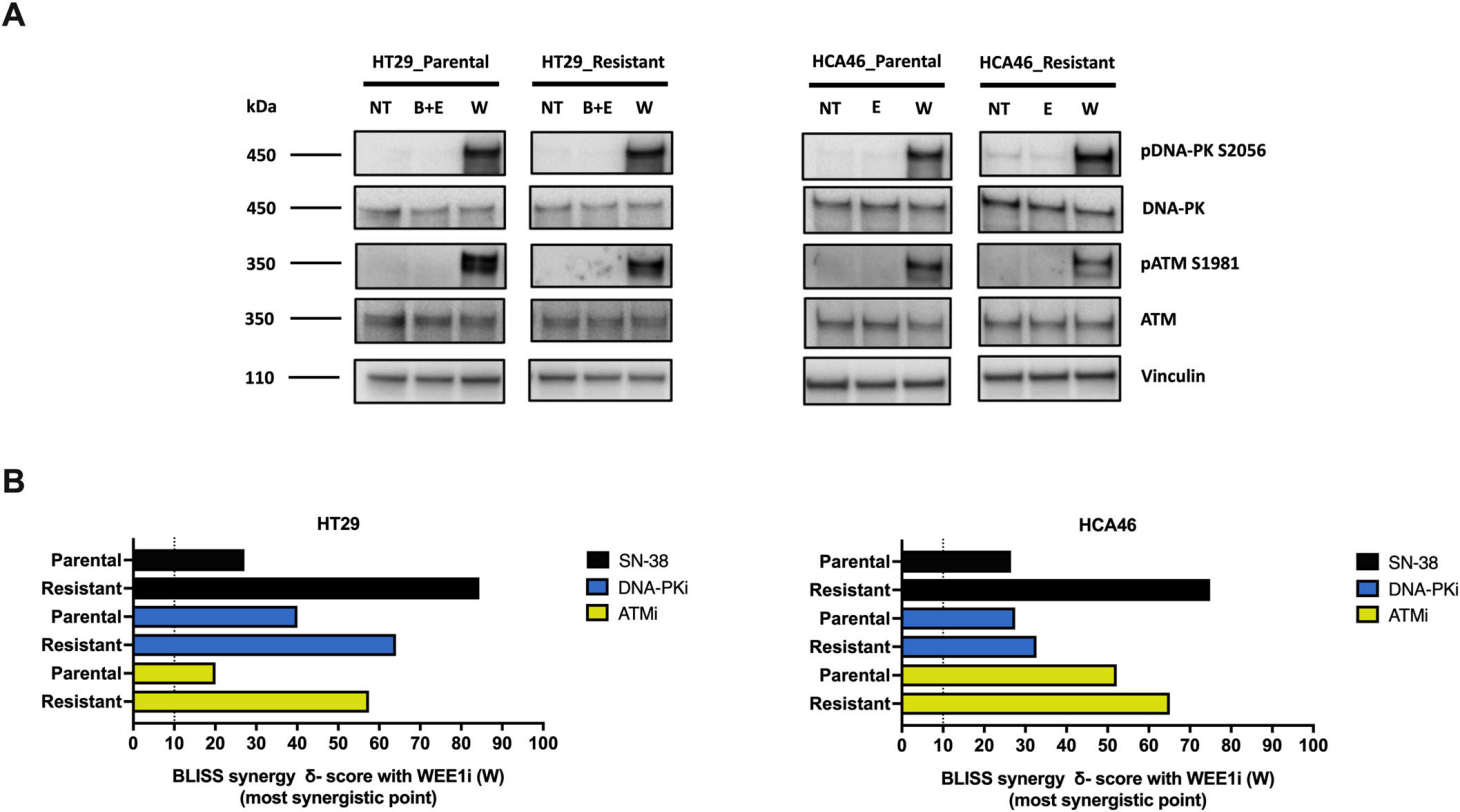
WEE1 inhibition synergises with chemotherapeutic agents or other DNA damage response inhibitors. (**A**) Immunoblot analysis of ATM and DNA-PK activation after 24 h treatment with targeted therapy (B + E): BRAFi-dabrafenib (4 µM, 24 h) plus EGFRi-cetuximab (5 µg/ml, 24 h) for HT29/E: EGFRi-cetuximab (25 µg/ml, 24 h) for HCA46 or W: WEE1i-adavosertib (300 nM, 8 h). Vinculin was used as a loading control. Images are representative of *n* = 3 independent biological replicates. (**B**) BLISS synergy scores for combinations of WEE1 inhibition with ATM inhibition, DNA-PK inhibition, or SN-38 in HT29 and HCA46 cell pairs derived from 7-day crystal violet viability assays. Source data are available online for this figure.

**Figure EV11:**
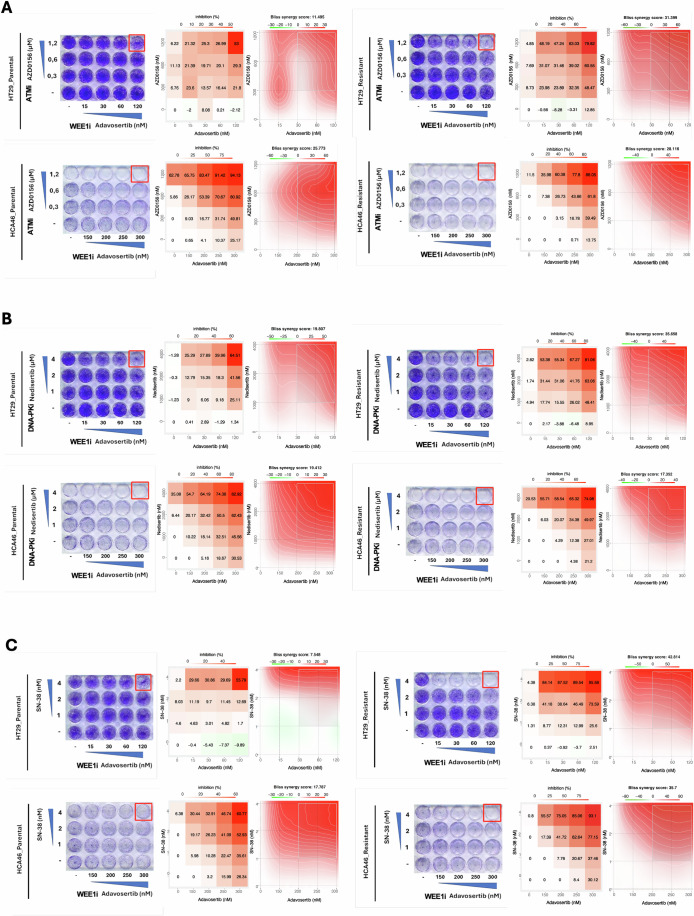
Evaluation of synergism by combining WEE1i with different DDRi (ATM, DNA-PK) or chemotherapeutic agents (SN-38). The indicated parental and resistant cells were seeded on day 0 and treated the following day with combinations of sublethal concentrations of a W:WEE1-adavosertib (15–120 nM) for HT29 and (150–300 nM) for HCA46 together with ATMi-AZD0156 (300–1200 nM), DNA-Pki Nedisertib (1–4 µM), or SN-38-active compound of irinotecan (1–4 nM), arranged in a dose–response matrix for synergy score calculation (see Methods). After 6 days of treatment, cells were fixed and stained with crystal violet. Quantification was performed by measuring the absorbance of the dye dissolved in acetic acid. Panel (**A**) shows combinations of WEE1 inhibitor with ATMi; panel (**B**) shows combinations with DNA-PKi; panel (**C**) shows combinations with SN-38. Images are representative of two biological replicates.

Despite inducing substantial DNA damage (Fig. 6A,B), WEE1 inhibition resulted in minimal RAD51 foci formation in both parental and resistant models (Fig. 7A), suggesting an impairment of RAD51-mediated repair. To further validate this observation, we performed co-immunofluorescence staining for RAD51 and γ-H2AX foci formation in irradiated (IR) cells pre-treated with sublethal doses of WEE1i (Fig. 7B,C). As expected, irradiation alone increased DNA damage (i.e. γ-H2AX levels) in both parental and resistant models (Fig. 7B) and induced robust RAD51 foci formation to a comparable extent in each pair, confirming their HR proficiency (Fig. 7C). However, RAD51 foci levels resulted significantly downregulated when irradiation followed sublethal doses of WEE1i (Fig. 7C), despite the high levels of DNA damage (Fig. 7B). Notably, both resistant models exhibited an even lower percentage of RAD51-positive nuclei compared with parental cells, with fewer than 20% of cells displaying RAD51 foci, indicating a profound impairment of RAD51-mediated repair capacity with potential therapeutic relevance. Building on these findings and considering that WEE1 inhibition enhanced DNA-PK and ATM phosphorylation (Fig. 8A), we tested combination strategies pairing WEE1i with ATM inhibitors (ATMi) (Fig. EV11A) or DNA-PK inhibitors (DNA-PKi) (Fig. EV11B) in both parental and resistant models. In addition, we tested SN-38 (Fig. EV11C), the active metabolite of irinotecan, a chemotherapeutic agent routinely used in CRC that induces replication stress through topoisomerase I inhibition (Voigt et al, 1998). All combinations displayed synergistic activity in both parental and resistant cells, with synergy scores exceeding 10 (Figs. 8B and EV11A–C). Notably, the combination of WEE1i with SN-38 (Fig. EV11C) emerged as the most synergistic and effective, particularly in resistant models compared with their parental counterparts (Synergy score >70 in resistant versus score <30 in parental cells).

Mechanistically, SN-38 is known to stabilize Top1–DNA cleavage complexes, thereby generating replication-associated DNA lesions that promote replication fork stalling and collapse. Concomitant WEE1 inhibition abrogates checkpoint control and we propose that the combination pushes replication stress beyond a tolerable threshold, resulting in excessive accumulation of DNA damage and cell death.

Collectively, these results provide a mechanistic rationale for the strong and consistent synergy observed between WEE1 inhibition and SN-38 (Fig. EV11C) and further support the translational potential of this combination as a strategy to overcome acquired resistance to EGFR-targeted therapies in CRC.

### Validation in clinically relevant patient-derived colorectal cancer models

To further strengthen the translational relevance of our findings, we evaluated the efficacy of WEE1 inhibition within the framework of secondary resistance to targeted therapies leveraging two distinct and unique patient-derived organoid models. The first model (the HER-B model) originates from an MSS HER2-amplified tumor nodule excised post-treatment with the anti-HER2 regimen consisting of trastuzumab emtansine (T-DM1) combined with pertuzumab in a patient that was enrolled in the HERACLES-B trial (Sartore-Bianchi et al, 2020).

**Figure EV12:**
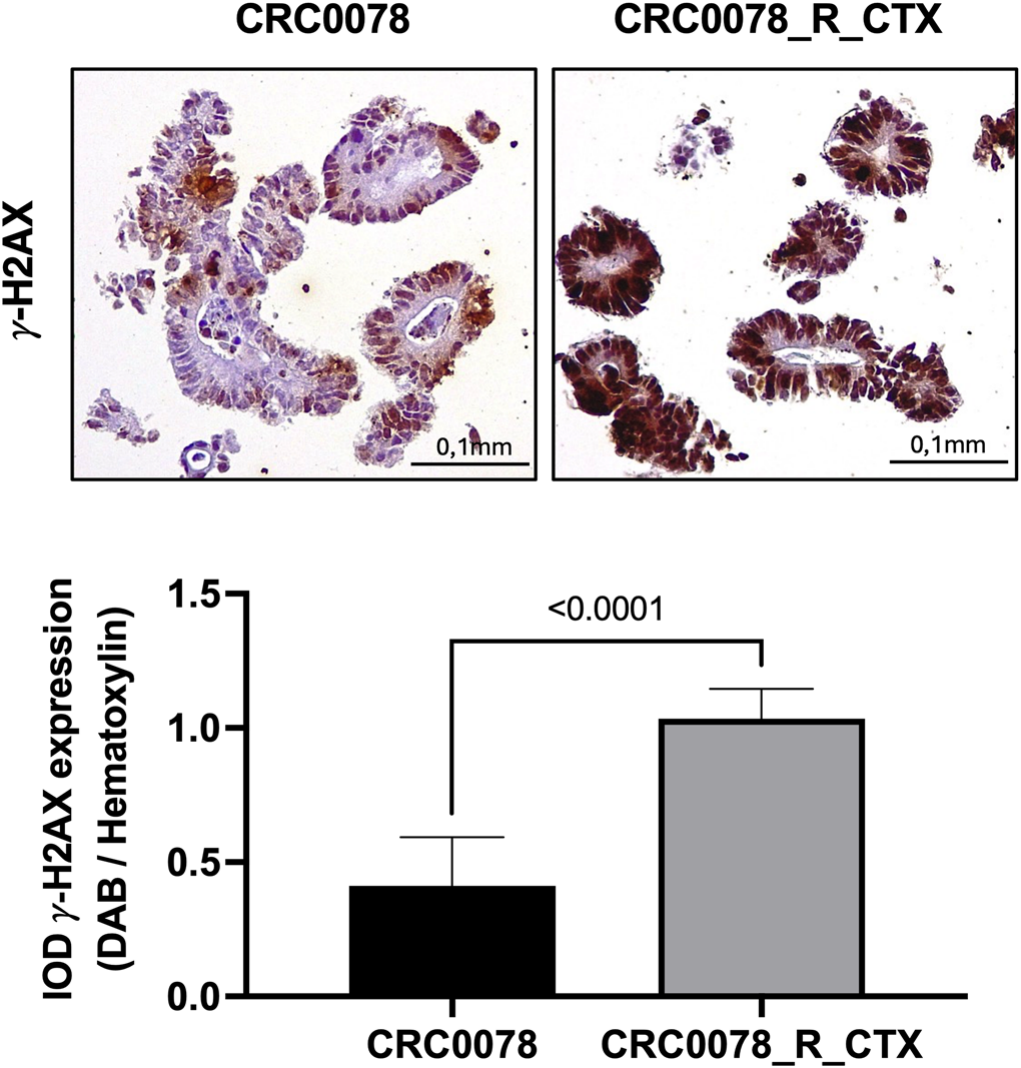
γ-H2AX expression analysis in patient-derived xenograft FFPE. Representative immunohistochemistry images showing γ-H2AX staining as a marker of DNA damage in patient-derived organoids under basal conditions. Organoids were cultured embedded in matrix until reaching adequate size, collected, formalin fixed and paraffin-embedded (FFPE), sectioned, and stained. Scale bar, 0.1 mm. Immunoreactivity was quantified with the NIH Image J software using the color-deconvolution plug-in that has a built-in vector for separating hematoxylin (H) and diaminobenzidine (DAB) stainings. After color deconvolution DAB images and hematoxylin are processed separately. Data are presented as mean ± SD from *n* = 10 images/condition. Statistical significance was assessed using a two-tailed Mann–Whitney U test.

**Figure 9:**
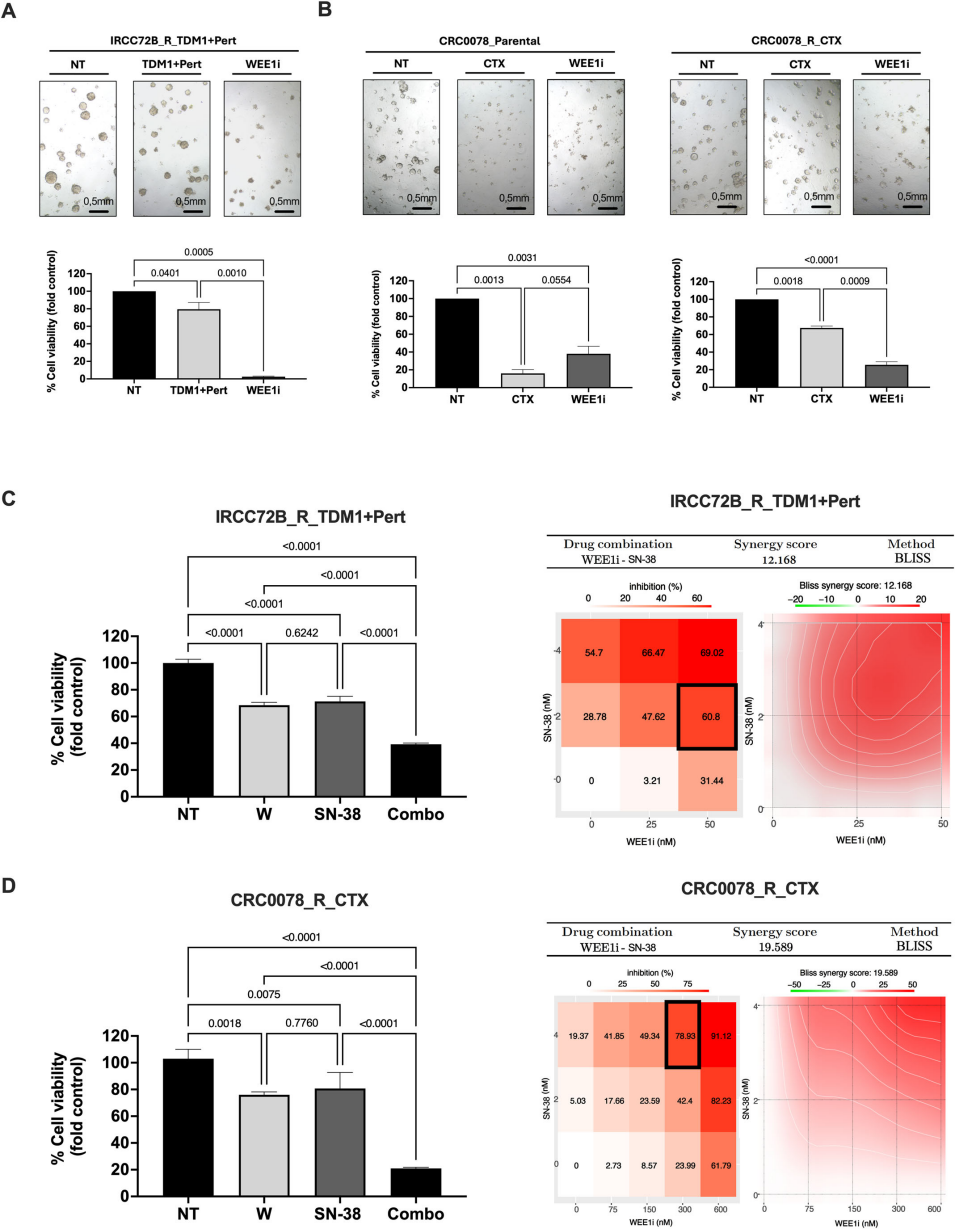
WEE1 inhibition is effective in patient-derived models with acquired resistance to EGFR pathway blockade. Patient-derived organoids were dissociated to single cells (5 × 10⁵) and embedded on day 0. Treatments were applied from day 4 for 6 days and bright-field images acquired prior to viability assessment. (**A**) Viability of IRCC72B_TDM1+Pert organoids treated with TDM1 + pertuzumab or adavosertib. (**B**) Viability of CRC0078 parental and cetuximab-resistant organoids treated with cetuximab (CTX) or adavosertib (WEE1i). (**C**, **D**) BLISS synergy analysis of adavosertib combined with SN-38 in resistant PDO models. Drug concentrations are indicated in the heatmap. Cell viability was measured using CellTiter-Glo. Data are presented as mean ± SD from *n* = 2 independent biological experiments performed in technical quadruplicate. Statistical significance was assessed using one-way ANOVA with Tukey’s multiple-comparisons test. Source data are available online for this figure.

The second model was derived from a quadruple wild-type (4WT), cetuximab-sensitive colorectal tumor obtained from a patient identified as CRC0078. We previously generated a unique in vivo-induced PDX model of acquired resistance to anti-EGFR therapy (Lorenzato et al, 2020) and later developed PDXO from the vehicle-treated control and from the cetuximab-treated resistant tumor, thereby obtaining a clinically relevant paired model of acquired resistance. Consistent with our observations in 2D systems, the resistant PDXO displayed higher basal DNA damage compared with its parental counterpart, as demonstrated by γ-H2AX immunohistochemical analysis (Fig. EV12). Notably, in both patient-derived models, WEE1 inhibition demonstrated significant therapeutic efficacy, effectively overcoming resistance to prior targeted therapies (Fig. 9A,B). Finally, prompted by the strong synergy observed in 2D systems, we evaluated the combination of sublethal doses of WEE1 inhibitor and SN-38 in resistant 3D organoid models. This combination exhibited robust synergistic activity (Fig. 9C,D), consistent with the mechanistic interaction previously described. Collectively, these data validate our findings in clinically relevant 3D models and further support WEE1 inhibition, either as monotherapy or in combination with Irinotecan-based regimens, as a promising therapeutic strategy for colorectal cancer patients who develop resistance to anti-EGFR-based targeted therapies.

## Discussion

The emergence of acquired resistance in colorectal cancer (CRC) is a significant challenge in oncology, undermining the long-term efficacy of targeted therapies (Ríos-Hoyo et al, 2024). Although patients initially benefit from EGFR-targeted therapies and other MAPK pathway inhibitors, resistance inevitably emerges through various pre-existing or adaptive mechanisms (Di Nicolantonio et al, 2021; Russo et al, 2019), including genetic mutations, activation of alternative signaling pathways, metabolic reprogramming and phenotypic plasticity. Despite this heterogeneity, many resistance mechanisms often converge on sustained ERK pathway activation (Misale et al, 2014a), that fuels oncogenic signaling and triggers RS (Primo and Teixeira, 2019). Persistent RS increases reliance on the DNA damage response (DDR), a network that detects DNA lesions and orchestrates repair, checkpoint activation, or apoptosis. While DDR inhibitors have shown clinical promise in several malignancies, their role in overcoming acquired resistance to targeted therapies in CRC remains insufficiently explored (Groelly et al, 2023).

In this study, we evaluate the potential use of DDRi as a novel and promising approach for treating mCRC patients who have developed secondary resistance to anti-EGFR targeted therapies. Leveraging our ARes platform, which includes CRC cell pairs with acquired resistance, xenograft models and patient-derived organoids, we found that inhibitors targeting replication stress were particularly effective, with WEE1 inhibitors showing the strongest and most consistent activity. Resistant models displayed elevated basal RS and DNA damage, likely reflecting chronic drug-induced stress. We hypothesize that this state renders resistant cells increasingly dependent on the ATR–CHK1–WEE1 axis for survival, thereby sensitizing them to WEE1 inhibition. A recent study from Dias and colleagues (Dias et al, 2024) highlighted how the paradoxical activation of oncogenic signaling, achieved artificially by using PP2A phosphatase inhibitors, could be exploited therapeutically. Similarly, we postulate that oncogenic signaling activated by chronic treatment with targeted agents might drive a resistance state characterized by persistent DNA damage and RS, which can be therapeutically leveraged by WEE1 targeting. While resistant cells adapt to tolerate anti-EGFR therapy-induced stress, they remain highly vulnerable when WEE1 is inhibited, leading to accelerated G2/M entry and cell death.

Given our hypothesis that the acquisition of resistance is accompanied by increased replication stress, we first sought to determine whether the elevated baseline DNA damage observed in resistant cells could be attributed to heightened replication stress. DNA fiber assays performed under basal conditions revealed a significantly reduced CldU/IdU ratio in resistant derivatives compared to parental cells, indicating increased RS. We then investigated the underlying cause of this phenotype and found that resistant cells displayed a deregulated nucleotide metabolic profile. Importantly, exogenous nucleoside supplementation restored replication fork dynamics and reduced γ-H2AX levels in resistant HT29 and HCA46 models, linking RS directly to nucleotide imbalance. These findings suggest that altered nucleotide metabolism contributes to replication vulnerability and may serve as a potential functional biomarker of RS in resistant CRC. We next investigated how WEE1 inhibition affected DNA repair capacity. Following ionizing radiation, both parental and resistant cells recruited RAD51 to sites of damage, indicating preserved homologous recombination (HR) proficiency. However, WEE1 inhibition triggered substantial DNA damage while markedly impairing RAD51 foci formation, consistent with a WEE1-induced defective HR-mediated repair (Duda et al, 2017). This defect, confirmed across multiple assays, was more pronounced in the acquired resistance models, which displayed elevated basal RAD51 foci, likely reflecting chronic endogenous damage and a greater reliance on DDR signaling. Although the precise mechanism by which WEE1 inhibition suppresses RAD51 recruitment remains to be clarified, similar observations have been reported in breast and ovarian cancers (Ha et al, 2020; Roering et al, 2022). Notably, even sublethal WEE1 inhibitor doses induced an HR-deficient-like phenotype in resistant CRC models, suggesting a therapeutically exploitable vulnerability. Given that most CRCs are HR proficient (Mauri et al, 2020), pharmacologically induced HR impairment may open up a new scenario for leveraging synthetic lethal interactions with other DNA damaging drugs currently used for CRC treatment or under clinical development.

Combination of WEE1i with other therapies has provided promising results in other types of cancers, including recurrent uterine serous cancer with *TP53* mutation (Liu et al, 2021), platinum resistant high-grade serous ovarian cancer (Leijen et al, 2016; Lheureux et al, 2021; Moore et al, 2022), advanced HNSCC (Méndez et al, 2018) and unresectable pancreatic cancer (Cuneo et al, 2019). In CRC, the first-in-class WEE1i adavosertib showed encouraging efficacy and safety in the randomized FOCUS4-C trial (Seligmann et al, 2021), enrolling KRAS/TP53-mutant patients in the maintenance setting. Our data expand this benefit to KRAS wild-type and BRAF-mutant CRC, consistent with previous large-scale screening results (Durinikova et al, 2022), and extend to the novel acquired resistance setting, including tumors bearing EGFR ECD and KRAS mutations and HER2 amplification. It is important to note that adavosertib completed most phase II trials in various solid tumors, but was terminated due to undesirable toxicities, likely due to off-target effects, although in mCRC it appeared to be better tolerated compared to other types of tumors (Seligmann et al, 2021), suggesting that this drug might have a potentially wider therapeutic window in treating this cancer type. In our experiments, we minimized such effects by using low or sublethal concentrations, ensuring observed phenotypes primarily reflected WEE1 blockade. To date, no trials have addressed WEE1 inhibition specifically in CRC patients progressing on anti-EGFR-based regimens and our preclinical results highlight additional opportunities. Combination of sublethal WEE1i doses with DNA-damaging agents such as SN-38 (the active metabolite of irinotecan), or with other DDRi such as ATMi or DNA-PKi, showed synergistic effects in cells and patient-derived models, with even greater efficacy in resistant tumors, supporting further exploration of rational combinations centered on WEE1 inhibition. This concept is consistent with the emerging paradigm that therapy-resistant cancers often harbor targetable non-oncogene dependencies, particularly in DNA damage response pathways. From a translational perspective, these findings are highly relevant. Irinotecan-based regimens remain a backbone of CRC treatment and the strong and reproducible synergy between WEE1 inhibition and SN-38, especially in EGFR inhibitor-resistant models, provides a compelling mechanistic rationale for clinical evaluation of this combination. Pharmacological inhibition of WEE1 overrides protective checkpoints, and, when combined with SN-38, these effects push replication stress beyond a tolerable threshold, leading to catastrophic DNA damage accumulation and cell death.

Moreover, the preferential activity in resistant cells suggests the potential to selectively target tumors that have progressed on EGFR-targeted therapies, addressing a major unmet clinical need in metastatic CRC. Encouragingly, several next-generation WEE1 inhibitors—including ZN-c3, Debio 0123, IMP7068, SC0191, and SY-4835 are currently in early-phase clinical development, with potentially improved pharmacologic and toxicity profiles. These advances may facilitate the clinical translation of rational WEE1i-based combinations aimed at maximizing replication stress beyond tumor tolerance while preserving therapeutic windows.

Importantly, we do not propose that acquired resistance universally enhances WEE1 inhibitor sensitivity. Rather, sensitivity is generally maintained and may increase in contexts characterized by elevated RS and basal DNA damage. The identification of biomarkers predicting response to WEE1i remains an unmet need. In other cancers, features such as *cyclin E* amplification or *BRCA* mutations have guided patient selection (Cong et al, 2021; Fu et al, 2023). In CRC, we did not identify analogous alterations. Instead, our findings point to increased RS and basal DNA damage as functional predictors of sensitivity, supporting biopsy-based stratification approaches to identify patients whose tumors exhibit RS-associated vulnerabilities, such as high γ-H2AX levels, that may benefit from replication stress–targeting strategies. However, clinical validation is hampered by the very limited availability of post-progression surgical samples, which restricts confirmation in larger human cohorts. This gap underscores the importance of developing non-invasive biomarkers and integrating functional assays into translational pipelines.

In conclusion, our study identifies WEE1 inhibition as a therapeutically exploitable vulnerability in CRC, particularly in the setting of acquired resistance to anti-EGFR therapies. By targeting replication stress and impairing RAD51-mediated repair, WEE1 inhibition induces lethal DNA damage accumulation, especially when combined with DNA-damaging agents such as SN-38. The development of next-generation WEE1 inhibitors with improved selectivity and tolerability may facilitate clinical translation of rational combination strategies designed to maximize tumor-selective replication stress while preserving therapeutic windows. Collectively, these findings provide a strong preclinical rationale for integrating WEE1-targeted approaches into treatment paradigms for mCRC patients that progressed upon anti-EGFR-based therapies.

## Methods

**Table:** Reagents and tools table

Reagent/Resource	Reference or Source	Identifier or Catalog Number
**Experimental models**		
Human: DiFi cell line	Laboratory of J. Baselga	RRID:CVCL_6895
Human: DiFi_R1 cetuximab-resistant cell line	Laboratory of A.Bardelli	RRID:CVCL_A2BX
Human: HCA46 cell line	ECACC	RRID:CVCL_2468
Human: HCA46_R5 cetuximab-resistant cell line	Laboratory of A.Bardelli	N/A
Human: LIM1215 cell line	ECACC	RRID:CVCL_2574
Human: LIM1215_R cetuximab-resistant cell line	Laboratory of A.Bardelli	RRID:CVCL_A2CB
Human: OXCO2 cell line	ECACC	RRID:CVCL_2H53
Human: OXCO2_R cetuximab-resistant cell line	Laboratory of A.Bardelli	N/A
Human: HT29 cell line	NCI60	RRID:CVCL_0320
Human: HT29_R cell line	This study	N/A
Human: VACO432 cell line	Laboratory of B. Vogelstein	RRID: CVCL_5402
Human: VACO432_R cell line	This study	N/A
Human: SW837 cell line	ECACC	RRID:CVCL_1729
Human: SW837_R cell line	This study	N/A
Human: C106 cell line	ECACC	RRID:CVCL_M011
Human: C106_R cell line	This study	N/A
Human: SW1116 cell line	ATCC	RRID:CVCL_0544
Human: COLO678 cell line	Laboratory of A.Bardelli	RRID:CVCL_1129
Human: OXCO3 cell line	Laboratory of V. Cerundolo	RRID:CVCL_B452
Human: C80 cell line	Laboratory of A.Bardelli	RRID:CVCL_5249
NOD SCID mice (xenografts)	Axis Bioservices	N/A
Patient-derived organoids: IRCC72B PDXO	FPO-IRCCS Candiolo	N/A
Patient-derived organoids: CRC0078 PDXO	This study	N/A
Patient-derived organoids: CRC0078_CTX_R cetuximab-resistant PDXO	This study	N/A
**Antibodies**		
Anti-phospho-EGFR (Y1068)	Cell Signaling Technology	2234S/RRID:AB_331701
Anti-EGFR	Cell Signaling Technology	2232S/RRID:AB_331707
Anti-phospho-ERK (T202/Y204)	Cell Signaling Technology	9101S/RRID:AB_331646
Anti-ERK (1/2)	Cell Signaling Technology	9102S/RRID:AB_330744
Anti-phospho-H2AX (gamma) (Ser139)	Cell Signaling Technology	80312S/RRID:AB_2799949
Anti-H2AX	Cell Signaling Technology	7631S/RRID:AB_10860771
Anti-phospho-DNA-PK (Ser2056)	Cell Signaling Technology	68716S/RRID:AB_2939025
Anti-DNA-PK	Cell Signaling Technology	12311S/RRID:AB_2797881
Anti-phospho-ATM	Cell Signaling Technology	4526S/RRID:AB_2062663
Anti-ATM	Cell Signaling Technology	2873S/RRID:AB_2062659
Anti-Vinculin (clone V284)	MERCK-Millipore	05-386/RRID:AB_309711
Anti-RAD51	Antibodies.com	A351
Alexa Fluor 647 goat anti-mouse IgG2a	Thermo Fisher Scientific	Cat A-21241/RRID:AB_2535810
Alexa Fluor 555 donkey anti-mouse	Thermo Fisher Scientific	Cat A-31570/RRID:AB_2536180
Alexa Fluor 546 Goat anti-Mouse IgG1	Thermo Fisher Scientific	Cat A-21123/RRID: AB_2535765
Alexa Fluor 488 Chicken anti-Rat IgG (H + L)	Thermo Fisher Scientific	Cat A-21470/RRID:AB_2535873
Alexa Fluor 488 donkey anti-rabbit	Thermo Fisher Scientific	Cat A-21206/RRID:AB_2535792
Anti-BrdU antibody [BU1/75 (ICR1)]	Abcam	ab6326
Mouse anti-BrdU, clone B44	BD Biosciences	Cat 347580/RRID:AB_400326
Mouse anti-ssDNA antibody clone TNT-3	Sigma	MAB3868
**Chemicals, Enzymes and other reagents**		
Dulbecco’s Modified Eagle Medium/F-12 (DMEM/F-12)	Thermo Fisher Scientific	11330032
Advanced DMEM/F-12 medium	Thermo Fisher Scientific	12634028
Dulbecco’s Modified Eagle Medium (DMEM) high glucose	Thermo Fisher Scientific	41965062
RPMI-1640	Thermo Fisher Scientific	61870010
Iscove’s Modified Dulbecco’s Medium (IMDM)	Thermo Fisher Scientific	31980022
McCoy’s 5A (Modified) medium	Thermo Fisher Scientific	16600082
Fetal Bovine Serum (FBS)	Sigma-Aldrich	F7524
Penicillin-Streptomycin	Thermo Fisher Scientific	15140163
L-Glutamine (200 mM)	Thermo Fisher Scientific	A2916801
Trypsin-EDTA (0.25%), phenol red.	Thermo Fisher Scientific	25200056
LDS & Reducing Agent	Invitrogen	NP0008
DMSO	Sigma-Aldrich	34943-M
SybrSafe DNA Gel Stain	Invitrogen	S33102
Ceralasertib (ATR inhibitor/ATRi)	MedChemExpress	HY-19323
Adavosertib (Wee1 inhibitor/WEE1i)	MedChemExpress	HY-10993
AZD0156 (ATM inhibitor/ATMi)	MedChemExpress	HY-100016
Nedisertib (DNA-PK inhibitor/DNA-PKi)	MedChemExpress	HY-101570
Dabrafenib	MedChemExpress	HY-14660
Hydroxyurea (HU)	Sigma	H8627
Cetuximab (Erbitux)	Merck KGaA	Provided by company
Sotorasib (AMG 510**)**	ChemGood	C-1499
SN-38	Selleckchem	S4908
Olaparib	Selleckchem	AZD2281, Ku-0059436, S1060
Staurosporine solution from Streptomyce	Sigma-Aldrich	S6942-200UL
PEG300	Aurogene s.r.l.	S6704-100ML
Tween-80	Aurogene s.r.l.	S6702-100ML
Propidium Iodide	Sigma-Aldrich	P3566
Crystal Violet Solution	Sigma-Aldrich	V5265-500ML
Fluoromount-G	SouthernBiotech	0100-01
DRAQ-5	Invitrogen	Cat 62251
DAPI	MERCK LIFE SCIENCE SRL	5087410001
Peroxidase substrate kit (for IHC, SK-4100)	Vector Lab	SK-4100
Mayers Hematoxylin	Diapath	C0303
Cultrex Basement Membrane Extract Type 2	Amsbio	3533-010-02
	Corning	354230
TrypLE Express Enzyme	Thermo Fisher Scientific	12587001
GlutaMAX™ Suplement	Thermo Fisher Scientific	35050061
Primocin	Aurogene s.r.l.	ant-pm-1
HEPES	MERCK LIFE SCIENCE SRL	H3375-25G
B27 Supplement	Thermo Fisher Scientific	17504044
N-Acetyl-L-cysteine	MERCK LIFE SCIENCE SRL	A9165-5G
Nicotinamide, 99%	MERCK LIFE SCIENCE SRL	N0636-100G
Gastrin I Human	MERCK LIFE SCIENCE SRL	G9145-1MG
A83-01	BIO-TECHNE S.R.L	2939/10
SB202190	BIO-TECHNE S.R.L	1264/10
Recombinant Human I Noggin	BIO-TECHNE S.R.L	6057-NG-01M
EGF	MERCK LIFE SCIENCE SRL	E9644-2MG
BCA Protein Assay Kit	Thermo Fisher Scientific	23225
Cell Titer-Glo Luminescent Cell Viability Assay	Promega	G7573
Mycoplasma Detection Kit (PCR)	ABM	G238
**Software**		
FlowJo™ Software (10.8.1)	BD	RRID:SCR_008520
CometScore software	TriTek Corp.	RRID:SCR_016352
ImageJ/Fiji	NIH	RRID:SCR_003070
SynergyFinder	O’Connor Group	RRID:SCR_019318
GraphPad Prism	GraphPad	RRID:SCR_002798
Image Lab	Bio-Rad	RRID:SCR_014210
Leica Application Suite X (LAS X)	Leica Microsystems	RRID:SCR_013673
Tecan Spark Control Magellan software	Tecan	RRID:SCR_021897
HP D300 Digital Dispenser	HP	RRID:SCR_027242
**Other**		
Tecan SPARK M10 plate reader	Tecan	30104686
Tecan D300e Digital Dispenser	Tecan	F0L56A
ChemiDoc Imaging System	Bio-Rad	RRID:SCR_019037
Leica TCS SP8 AOBS confocal microscope	Leica Microsystems	219183055

### Cell lines and cell authentication

For this study, we used a panel of 8 pairs (parental and resistant derivatives) of human colorectal cell lines (Table EV1). The parental cell lines DiFi (RRID:CVCL_6895) and VACO432 (RRID:CVCL_5402) were obtained from the laboratories of J. Baselga and B. Vogelstein, respectively. HCA46 (RRID:CVCL_2468), LIM1215 (RRID:CVCL_2574), OXCO2 (RRID:CVCL_2H53), SW837 (RRID:CVCL_1729), and C106 (RRID:CVCL_M011) were purchased from the European Collection of Authenticated Cell Cultures (ECACC). SW1116 (RRID:CVCL_0544) was obtained from the American Type Culture Collection (ATCC). HT29 (RRID:CVCL_0320) was sourced from the NCI-60 panel. COLO678 (RRID:CVCL_1129), C80 (RRID:CVCL_5249), and OXCO3 (RRID:CVCL_B452) were provided by the laboratories of A. Bardelli and V. Cerundolo, respectively. Cetuximab-resistant cells were established as previously described (Arena et al, 2015; Arena et al, 2016; Misale et al, 2014b; Misale et al, 2012) in the laboratory of A. Bardelli. The other four resistant derivatives were recently generated and are presented for the first time in this work. All four of the remaining derivatives (HT29_R, VACO432_R, SW837_R, C106_R) were established upon continuous treatment with respective concentrations of targeted therapy, (B + E: BRAFi-dabrafenib (4 µM) plus EGFRi-cetuximab (5 µg/ml) for HT29; B + E: BRAFi-dabrafenib (2 µM) plus EGFRi-cetuximab (5 µg/ml) for VACO432; K + E: KRASG12Ci-AMG510 (3 µM) plus EGFRi-cetuximab (15 µg/ml) for SW837; K + E: KRASG12Ci-AMG510 (0.5 µM) plus EGFRi-cetuximab (15 µg/ml) for C106) until the emergence of resistance. DiFi (RRID:CVCL_6895) and SW837 (RRID:CVCL_1729) cells were cultured in Dulbecco’s Modified Eagle Medium/Nutrient Mixture F-12 (DMEM/F-12; #11330032, Thermo Fisher Scientific), while LIM1215 (RRID:CVCL_2574) cells were grown in RPMI-1640 medium + GlutaMAX (#61870–010, Thermo Fisher Scientific). OXCO2 (RRID:CVCL_2H53) and C106 (RRID:CVCL_M011) cells were grown in Iscove’s Modified Dulbecco’s Medium (IMDM, GlutaMAX™ supplement, #31980022, Thermo Fisher Scientific). HT29 (RRID:CVCL_0320) and HCA46 (RRID:CVCL_2468) were grown in Dulbecco’s Modified Eagle Medium (DMEM) while VACO432 cells were grown in McCoy’s. Media were supplemented with 10% fetal bovine serum (FBS; Sigma-Aldrich), and 1% penicillin-streptomycin (#15140163, Thermo Fisher Scientific), and cells were maintained in exponential growth in 5% CO_2_/95% air in a humidified incubator at 37 °C. All resistant cells were maintained under continuous exposure to the same concentration of targeted therapy used during their generation. The other resistant derivatives were kept under constant treatment with the respective concentrations 4 µM dabrafenib+ 5 µg/ml cetuximab for HT29; 2 µM dabrafenib+ 5 µg/ml cetuximab for VACO432; 3 µM AMG510 + 15 µg/ml cetuximab for SW837; 0.5 µM AMG510 + 15 µg/ml cetuximab for C106. Cells were routinely screened for mycoplasma contamination using the PCR Mycoplasma Detection Kit (ABM) according to the manufacturer’s protocol. Authentication of each cell line was performed with the PowerPlex 16 HS system (Promega), using Short Tandem Repeats (STR) at 16 different loci as previously described (Arena et al, 2020). The microsatellite instability (MSI) status was evaluated as previously described (Arena et al, 2020).

### DDR inhibitors and drugs

Ceralasertib [ATR inhibitor (ATRi); HY-19323], adavosertib [Wee1 inhibitor (Wee1i); HY-10993], AZD0156 [ATM inhibitor (ATMi); HY-100016], nedisertib [DNA-PK inhibitor (DNA-PKi); HY-101570] and dabrafenib (CS-1641; HY-14660) were purchased from MedChemExpress, hydroxyurea (HU; H8627) from Sigma, cetuximab (Erbitux) was provided by The healthcare business of MerckKGaA, Darmstadt, Germany (CrossRef Funder ID: 10.13039/100009945) and AMG510 (C-1499) was purchased from ChemGood. SN-38 (S4908) and olaparib (AZD2281, Ku-0059436, S1060) were purchased from Selleckchem.

### DDRi screening

The sensitivity was tested in a 7-day-long proliferation assay. Cells were seeded in 48-well culture plates. The seeding numbers for each cell line were accordingly calculated based on the capacity to reach 80–90% confluency of control wells at the end of the assay (DiFi: 1 × 10^4^; HCA46: 1,2 × 10^4^; LIM1215: 4 × 10^3^; OXCO2: 1 × 10^4^; HT29: 3 × 10^3^; VACO432: 6 × 10^3^; C106: 1,2 × 10^4^; SW837: 1 × 10^4^). The following day, treatment was performed initially manually adding the targeted treatment (to which cells have developed resistance) at a fixed concentration then serial dilutions of ceralasertib (0.215–10 μmol/L), adavosertib (0.65–3 μmol/L), AZD-0156 (0.32–15 μmol/L), nedisertib (0.43–20 μmol/L) and olaparib (0.25–20 μmol/L). Vehicle controls were treated with DMSO only. All drugs were added using the Tecan D300e Digital Dispenser. Seven days later, the cell viability was assessed by Cell TiterGlo Luminescent Cell Viability assay (Promega) and measured by the Tecan SPARK M10 plate reader. Viability measured for each treatment condition was normalized to vehicle-treated controls.

### Genomic DNA extraction, whole-exome sequencing, and bioinformatic analysis

Genomic DNA samples were extracted from each cell line using Maxwell RSC Blood DNA Kit (Promega) and sent to Cogentech (Milan Italy) for sequencing. Data analysis was performed in-house following the procedure described below: Somatic variants were identified in both parental and resistant cell lines following the GATK Mutect2 best practices (https://gatk.broadinstitute.org/hc/en-us/articles/360037593851-Mutect2), with parameters optimized for whole-exome sequencing data as recommended by the GDC DNA-Seq variant calling pipeline (https://docs.gdc.cancer.gov/Data/Bioinformatics_Pipelines/DNA_Seq_Variant_Calling_Pipeline/). To identify variants acquired de novo in the resistant lines, we employed an in-house bash script that classified variants as de novo, shared, or lost relative to the parental reference.

### In vivo xenograft models

The in vivo study was conducted externally at the Axis Bio company (Testing Facility: Axis Bioservices, Biological Research Unit/Coleraine, BT51 3RP). Female NOD SCID mice aged 5–9 weeks were supplied by Envigo. Mice were housed in individually ventilated cages (IVC; maximum of five mice per cage), with individual animals identified by tail marking. Cages, bedding, and water were sanitized prior to use. Animals were provided with appropriate bedding for environmental enrichment and nesting material. All personnel entering the animal holding room wore suitable barrier clothing (e.g., Tyvek suits, gloves, appropriate footwear, and masks). Each cage was clearly labeled with a card indicating the number of animals, sex, strain, date of birth, study number, license number, start date, and treatment. Animals had ad libitum access to a standard certified commercial diet and water. Cages were changed weekly, with food and water replenished as necessary. The animal holding room was maintained under controlled conditions: temperature 20–24 °C, humidity 45–65%, and a 12-h light/dark cycle.

All experimental protocols were approved by the Axis Bioservices Animal Welfare and Ethical Review Committee, and all procedures were conducted in accordance with the Animal (Scientific Procedures) Act 1986. The relevant Project Licence number for the studies was PPL2885. In brief: 1 × 10^7^ HT29_Parental/HT29_Resistant and HCA46_Parental/HCA46_Resistant cells were implanted subcutaneously on the flank of female NOD SCID mice aged between 5 and 9 weeks, weighing approximately 18–26 g. When the tumors reached approximately 100–150 mm^3^, animals were randomly assigned to the different treatment arms (*n* = 8 mice per group). Treatment groups were defined as follows: parental HT29 and HCA46 xenografts received either vehicle or WEE1 inhibitor (WEE1i). Resistant HT29 and HCA46 xenografts received vehicle, WEE1i alone, targeted therapy alone (cetuximab for HCA46; dabrafenib plus cetuximab for HT29), or combination therapy (WEE1i plus EGFR inhibitor for HCA46; WEE1i plus BRAF inhibitor plus EGFR inhibitor for HT29). The treatment schedule was as follows: adavosertib (WEE1i) was administered twice daily (BID) by oral gavage at 60 mg/kg; cetuximab (EGFRi) was administered intraperitoneally twice weekly (BIW) at 10 mg/kg; and dabrafenib (BRAFi) was administered once daily (QD) by oral gavage at 15 mg/kg. Tumor volume was measured three times a week. The duration of treatment lasted 28 days at the end of which the tumor was excised. Early removal of an animal took place in the following circumstances: weight loss ≥20%, impairment of the animal (e.g. signs and symptoms, inability to eat/drink), tumor volume greater than 1700 mm^3^.

### Single-cell gel electrophoresis—comet assay

DNA damage measurements were performed by alkaline comet assay fully described in (Collins et al, 2023). Briefly, Cells were seeded in 6-well culture plates (10^6^ cells/well), treated the next day with Wee1 inhibitor adavosertib for 8 h and with targeted therapies for 24 h. For the 12-mini gel format, 60 μl of cell suspension was directly mixed with 120 μl of pre-warmed low melting point agarose (0.5%) at 37 °C on a fully frosted slides coated with 1.5% of normal melting point agarose. The slides were then immersed in cold lysis solution (2.5 M NaCl, 100 mM EDTA, 10 mM Trizma Base, 1% Triton X-100, pH = 10, 4 °C) for 40 min. After lysis, slides were placed in a horizontal electrophoresis chamber filled with cold alkaline solution (freshly prepared—0.3 M NaOH, 1 mM EDTA, 4 °C) incubated for 40 min in the dark for DNA unwinding and then electrophoresis was performed (1 V/cm, 30 min). After electrophoresis slides were washed in PBS and then in distilled water each for 10 min. For dehydration of mini gels, slides were placed in 70% ethanol for 15 min and then in 100% ethanol for 30 min. After air-drying, all samples were stained with SybrSafe DNA gel stain (Invitrogen, Massachusetts, USA) for scoring. Results are shown like a median of tail moment, which represents % of the DNA in the tail together with the tail length. Each replicate was scored using CometScore software.

### Western blotting

Cells were seeded in 6-well culture plates (5 × 10^5^ cells /well), treated the next day with WEE1i adavosertib 300 nM or with anti-EGFR cetuximab 50 µg/ml (HCA46) and BRAFi (dabrafenib 10 µM) + anti-EGFR (cetuximab 20 µg/ml) (HT29) for the indicated time. Cells were subsequently lysed in using boiling SDS buffer [50 mmol/L Tris-HCl (pH 7.5), 150 mmol/L NaCl, and 1% SDS] to extract total cellular proteins, quantified by the BCA Protein Assay Reagent kit (Thermo Fisher Scientific), and prepared using LDS and Reducing Agent (Invitrogen). Western blot analysis was performed with Enhanced Chemiluminescence System (GE Healthcare) and peroxidase-conjugated secondary antibodies (Amersham). The following primary antibodies were used for Western blotting: anti-phospho-EGFR (y1068) (Cell Signaling Technology, 2234S; 1:1000 RRID:AB_331701), anti-EGFR (Cell Signaling Technology, 2232S; 1:1000 RRID:AB_331707), anti-phospho-AKT (s473) (Cell Signaling Technology, 9271S; 1:1000 RRID:AB_329825), anti-AKT (Cell Signaling Technology, 9272S; 1:1000 RRID:AB_329827), anti-phospho-p44/42 MAPK (ERK 1/2) (Cell Signaling Technology, 9101S; 1:1000 RRID:AB_331646), anti-p44/42 MAPK (ERK 1/2) (Cell Signaling Technology, 9102S; 1:1000 RRID:AB_330744), anti-phospho-H2AX (gamma) (Cell Signaling Technology, 80312s; 1:1000 RRID:AB_2799949), anti-H2AX (Cell Signaling Technology, 7631s; 1:1000 RRID:AB_10860771), anti-phospho-DNA-PK (Ser2056; Cell Signaling Technology, 68716S; 1:1000 RRID:AB_2939025), anti-DNA-PK (Cell Signaling Technology, 12311S; 1:1000 RRID:AB_2797881), anti-phospho-ATM (Cell Signaling Technology, 4526S; 1:1000 RRID:AB_2062663), anti-ATM (Cell Signaling Technology, 2873S; 1:1000 RRID:AB_2062659), anti-Vinculin (clone V284) (MERCK-Millipore, 05-386; 1:1000 RRID:AB_309711). Detection of the chemiluminescent signal was performed with ChemiDoc Imaging System (Bio-Rad) (RRID:SCR_019037).

### Immunofluorescence analysis

Cells were seeded at a density of 1 × 10^5^ cells on a glass coverslip in a 24-well plate and were treated the next day with: Targeted treatment (cetuximab 25 μg/L or dabrafenib+cetuximab 4 μmol/L + 5 μg/L for 24 h), adavosertib 0.1 or 0.3 μmol/L for 8 h, HU at a concentration of 2.5 mmol/L for 24 h or supplementation with nucleoside mixture (EmbryoMax® Nucleoside Mix, 100× stock; Merck/Millipore) for 24 h. At the end of treatment, cells were fixed in 4% paraformaldehyde for 20 min at room temperature and permeabilized with 0.3% Triton-X100 in PBS for 10 min. Cells were incubated at room temperature with 3% BSA 0.03% Triton-X100 in PBS for 60 min, followed by incubation overnight at 4 °C with the following primary antibodies diluted in PBS containing 1% of BSA: anti-phospho-Histone H2AX (Ser139; Bethyl Laboratories A300-081A; 1:600 RRID:AB_203288) and anti-RAD51 (Millipore ABE257; 1:100 RRID:AB_10850319). After washing, cells were fluorescently labeled with Alexa Fluor 555 donkey anti-mouse antibody (Molecular Probes; 1:400) (Thermo Fisher Scientific Cat# A-31570, RRID:AB_2536180) and Alexa Fluor 488 donkey anti-Rabbit antibody (Molecular Probes; 1:400) (Thermo Fisher Scientific Cat# A-21206, RRID:AB_2535792) for 1 h at room temperature. Nuclei were stained with DRAQ-5 in blue (Invitrogen Cat# 62251). A Leica TCS SP8 AOBS confocal microscope (Leica Microsystems) under a 63× oil objective was used to detect γ-H2AX, and RAD51 foci stainings. For the detection of nuclear-localized foci, images were captured at 3 individual z-planes and were merged using the “Z Project” function in ImageJ (RRID:SCR_003070). Individual nuclei were scored for foci positivity as identified based upon signal intensity above general background staining levels and present within the nucleus as assessed by DRAQ-5 staining and/or DAPI. Cells containing ≥5 distinct foci were defined as foci-positive, and the percentage of positive nuclei was calculated as [(number of foci-positive nuclei)/(number of nuclei scored)] *100. A minimum of 400 nuclei per each condition were scored.

### ROS production assay

Cells were seeded in a 96-well white-walled plate (10 × 10^3^ cells/well for HT29 parental and resistant cells and 12 × 10^3^ cells/well for HCA46 parental and resistant cells) and incubated overnight for their attachment. The following day, parental and resistant cells were treated with targeted agents, such as dabrafenib (4 µM) and cetuximab (5 µg/ml) for the HT29 cell pair and cetuximab (50 µg/ml) for HCA46 cell pair. After 48 h ROS were measured by ROS-Glo™ H2O2 Assay (Promega) according to the manufacturer’s protocol. Luminescence was measured using a plate-reading luminometer (TECAN Spark 10 M) and the resulting data were normalized to untreated cells.

### Untargeted metabolomics

Untargeted metabolomics profiling was performed by Metabolon, Inc. (Durham, NC, USA) using their global metabolomics platform. Samples were processed by protein precipitation with methanol, followed by centrifugation to recover metabolites. Extracts were analyzed using ultra-high-performance liquid chromatography–tandem mass spectrometry (UHPLC-MS/MS) in both positive and negative ionization modes, and by gas chromatography–mass spectrometry (GC-MS). Metabolites were identified by comparison to a library of authenticated standards based on retention index, accurate mass, and MS/MS spectral data.

Data extraction, peak integration, and quality control were performed using Metabolon’s proprietary informatics pipeline. Raw values were log-transformed and normalized to correct for inter-day variation and batch effects, and missing values were imputed with the minimum observed value for each compound. Statistical analyses, including univariate testing (e.g., Welch’s two-sample t-tests or ANOVA as appropriate), were applied to assess differential metabolite abundance between groups.

### DNA fiber assay

CRC cells were seeded at 2 × 10^5^ cell density in 6-well plate and the next day cells were supplemented with nucleoside mixture (EmbryoMax® Nucleoside Mix, 100× stock; Merck/Millipore) for 24 h before labeling. Sequential pulse labeling was performed with 25 µM iododeoxyuridine (IdU, Sigma) for 20 min, followed by 250 µM chlorodeoxyuridine (CldU, Sigma) for 40 min. Cells were washed with PBS after each labeling step, trypsinized for 3 min, resuspended in cold PBS, and counted. Cell suspensions were adjusted to 4–15 × 10^5^ cells/ml and maintained on ice until processing.

DNA spreading was performed by placing two microliters of cell suspension on a glass slide, allowed to air-dry partially (∼7 min), and lysed with 7 µl spreading buffer (200 mM Tris-HCl pH 7.4, 50 mM EDTA, 0.5% SDS) for 5 min. Slides were tilted (25–40°) to allow DNA fibers to spread by gravity and subsequently air-dried for 4–12 h. DNA was fixed in methanol/acetic acid (3:1) for 10 min, air-dried, and stored at 4 °C overnight.

Slides were washed with water, denatured in 2.5 M HCl for 1 h, and rinsed in PBS. Following fixation with 4% paraformaldehyde (10 min), slides were blocked in PBS containing 1% BSA and 0.1% Tween-20 for 1 h at 4 °C. For thymidine analogue detection, mouse anti-BrdU antibody (clone B44, BD Biosciences, 1:500, specific for IdU) and rat anti-BrdU antibody (clone BU1/75, Abcam, 1:500, specific for CldU) were incubated for 1 h at room temperature. After three washes, secondary antibodies Alexa Fluor 555 goat anti-rat IgG (1:500) and Alexa Fluor 488 F(ab′)2 goat anti-mouse IgG (1:500, Thermo Fisher) were applied for 2 h in the dark. After washing, slides were mounted with Fluoromount-G (SouthernBiotech) and stored at −20 °C until imaging. Images were acquired on a confocal microscope using a 63× oil-immersion objective. Channels were set to detect Alexa Fluor 555 (CIdU, 568 nm) and Alexa Fluor 488 (ldU, 488 nm). A minimum of 150 fibers were scored per condition from three independent experiments. Replication track lengths were measured using TIFF export files and analyzed with ImageJ/Fiji.

### Cell cycle analysis

CRC cells were seeded at (5 × 10^5^ cells/well) in a complete medium in 6-well plastic culture plates at day 0. The following day, the indicated treatments were added BRAFi+anti-EGFR (4 μM dabrafenib + 5 μg/ml cetuximab) for HT29 and anti-EGFR (25 μg/ml cetuximab) for HCA46, WEE1i (300 nM adavosertib). In the same way, control cell lines intrinsically sensitive or resistant to adavosertib were seeded (5 × 10^5^ cells/well) and treated with the same concentration of WEE1i. Plates were incubated at 37 °C in 5% CO_2_ overnight. Following treatment, cells were stained with Propidium Iodide (Sigma-Aldrich) following manufacturer’s instructions and analyzed by flow cytometry. The percentage of cells in each phase was calculated using FlowJo software (RRID:SCR_008520).

### BLISS synergy scoring

To perform this assay, cells were seeded in 24-well plates on day 0. The following day cells were treated with different concentrations of drugs (sublethal dosage selected) both as single and combined creating a matrix (see Fig. EV9F). At day 7 post treatment all cells were fixed with 4% paraformaldehyde for 20 min at room temperature. After that, cells were colored using crystal violet solution for 30–60 min Plates were washed with water and left to dry at room temperature. After drying, images were taken for each condition, crystal violet was dissolved in acetic acid. Absorbance was then measured calculating viability scoring normalized to the untreated wells. Matrix data were then uploaded to the free version of SynergyFinder program (RRID:SCR_019318) calculating the BLISS synergy score for each combination of drugs (Ianevski et al, 2022).

To calculate this in the PDXOs, matrix data were obtained from viability measurements post-treatment with combinations of WEE1i and SN-38 at sublethal dosages as described in the drug screening section and were then uploaded to the free version of the SynergyFinder program (RRID:SCR_019318) calculating the BLISS synergy score for each combination of drugs (Ianevski et al, 2022).

### Organoid establishment, ethics and drug screening

Original tumor samples were collected from patients treated at the Niguarda Cancer Center in Milan, Italy, after obtaining written consent and used to establish PDX models. The study was conducted under the approval of the local Ethical Committee of the institutions (for Niguarda Cancer Center: study 194/2010, n.22, date 24th February 2010, for Candiolo Cancer Center: PROFILING study, 001-IRCC-00IIS-10, 6.0 version, date 24th April 2015). All procedures involving human samples were performed in accordance with the Declaration of Helsinki and the Department of Health and Human Services Belmont Report. For the IRCC72B PDXO model (patient #3 in Arena et al, Clin Cancer Res 2020), the donor was a 76-year-old female with stage IV MSS, HER2-amplified rectal cancer with liver metastases. The CRC0078 PDXO was derived from the xenopatient collection established at the Candiolo Cancer Institute, FPO-IRCCS under the PROFILING study (001-IRCC-00IIS-10); individual demographic details for this patient are not publicly available due to privacy constraints associated with the institutional consent framework. Detailed clinicopathological characteristics of the xenopatient cohort from which CRC0078 originates have been previously reported (Bertotti et al, Cancer Discov 2011; Leto, Grassi et al, Nat Commun 2024). Organoids (PDXO) were established from PDX explants or cryopreserved material. Briefly, tumor specimens were chopped with a scalpel and washed with PBS. After centrifugation, the final cell preparation was embedded in Matrigel (Corning) or Cultrex Basement Membrane Extract (BME, R&D Systems) and dispensed onto 24-well plates (Corning). After 10–20 min at 37 °C, culture medium was added. Complete PDXO medium composition is the following: DMEM/F12 supplemented with 50 U/ml penicillin-streptomycin, 2 mmol/L L-glutamine, 1 mmol/L n-Acetyl Cysteine, B27 (Thermo Fisher Scientific), N2 (Thermo Fisher Scientific), and 20 ng/mL EGF (Sigma-Aldrich). Organoids were tested for Mycoplasma and maintained at 37 °C in a humidified atmosphere of 5% CO_2_.

In order to test the effects of the drugs of interest, organoids were enzymatically dissociated using TrypLE Express Enzyme for 10 to 20 min at 37 °C to obtain single-cell suspensions. These cells were then seeded at a density of 4000 to 6000 cells per well in 96-well plates that were precoated with basement membrane extract (BME; Cultrex BME Type 2; Amsbio). An additional 100 μL of growth media containing 2% BME was overlayed on top. Drug treatments began on day 4 after seeding. Organoids were treated in fresh 150 μL medium containing 2% BME, with increasing doses of targeted treatment (trastuzumab emtansine [T-DM1] in combination with pertuzumab in the HER model or cetuximab in the CRC0078 model), WEE1 inhibitor (WEE1i) or SN-38 in technical quadruplicates. These doses covered sublethal and physiologic concentrations of the drugs. Treatments were administered automatically using a Tecan D300e Digital Dispenser. A total of 4 μmol L MG-132 was used as a positive control, while DMSO served as a negative control. Cell viability was assessed at the end of the experiment, 7 days after treatment, using the CellTiter-Glo Luminescent Cell Viability Assay (Promega). All experiments were independently repeated at least twice, and the final results are expressed as the average of biological replicates.

### Immunohistochemical staining in organoids

For immunohistochemistry (IHC) analyses, biological samples were sliced into 4-μm-thick sections. To quench endogenous peroxidase activity, the slides were treated with 0.3% hydrogen peroxide in distilled water. These sections were then deparaffinized in xylene and rehydrated through a series of decreasing concentrations of ethyl alcohol (100%, 95%, 80%, and 70%), followed by rinsing in distilled water. Antigen retrieval was performed using a preheated target retrieval solution for 30 min. Subsequently, the slides were incubated in a closed humid chamber overnight at 4 °C with a solution containing 5% goat serum, 0.1% Tween 20, 0.3% tritonX100 along with anti-γ-H2AX at a dilution of 1:250. The binding of the antibody was detected using a polymer detection kit (GAR-HRP, Microtech), followed by a diaminobenzidine (DAB) chromogen reaction using a Peroxidase substrate kit (SK-4100; Vector Lab). Finally, all sections were counterstained with Mayer’s Hematoxylin (Diapath, C0305) and visualized using a bright-field microscope (Leica DM750). 12 images per each model were acquired and considered for the quantification.

Immunoreactivity was quantified with the NIH Image J software using the color-deconvolution plug-in that has a built-in vector for separating hematoxylin (H) and diaminobenzidine (DAB) stainings. After color deconvolution DAB images and hematoxylin are processed separately. Quantification is expressed as IOD (DAB/H).

### Statistical analysis

Data are presented as mean ± SEM or SD, as indicated in the respective figure legends. Sample size was chosen based on established practice in the field and are reported in the respective figure legends and Methods sections. For in vitro experiments, all cell lines that successfully grew under the indicated conditions were included; no samples were excluded from the analyses unless technical failure occurred, as specified in the individual assays. For in vivo xenograft studies, mice bearing tumors of 100–150 mm³ were randomly assigned to treatment groups (*n* = 8 per group); animals were excluded only if tumor engraftment failed prior to randomization. Investigators were not blinded to group allocation during treatment administration or data acquisition, as the nature of the experiments (e.g., drug dosing schedules, cell line identity) precluded blinding; however, quantitative readouts were obtained using automated or semi-automated analysis tools to minimize subjective bias. Statistical analyses were performed using GraphPad Prism software (RRID:SCR_002798). For comparisons between two groups, a two-tailed Mann–Whitney U test was used for non-parametric data, whereas Welch’s two-sample t-test was applied when appropriate (e.g., metabolomics analyses). For comparisons involving more than two groups, one-way ANOVA followed by Tukey’s multiple-comparisons test was used for normally distributed data, while the Kruskal–Wallis test followed by Dunn’s multiple-comparisons correction was applied for non-parametric data (e.g., comet assay analyses). Two-way ANOVA followed by Tukey’s multiple-comparisons test was used for experiments involving two independent variables (e.g., viability assays). A *P* value < 0.05 was considered statistically significant. For all images, exact *P*-values are reported.

## Supplementary information

Table EV1

Peer Review File

Expanded View Figures

## Data Availability

The source data of this paper are collected in the following database record: https://www.ebi.ac.uk/biostudies/studies/S-BSST2901?query=%20S-BSST2901. Whole-exome sequencing data produced in this study are deposited in the following database https://www.ebi.ac.uk/ena/browser/view/PRJEB108852. The source data of this paper are collected in the following database record: biostudies:S-SCDT-10_1038-S44321-026-00434-4.
