# Supplementary material for: WEE1 kinase inhibition to overcome acquired resistance to targeted therapies in colorectal cancer
Source: EMBO Mol Med. 2026 May 19;18(6):2322–59. doi: 10.1038/s44321-026-00434-4 (PMC13269805; doi:10.1038/s44321-026-00434-4)
Supplement: Supplementary file 1 — Table EV1 [file 44321_2026_434_MOESM1_ESM.pdf]

Table EV1. List of cell models (ARes platform) with acquired resistance to different molecularly targeted therapies
(MSS=stable microsatellites. MSI=unstable microsatellites)

Pair nr.	Status	Cell lines	Microsatellite status	Acquired resistance to: Target	Drug
1	Parental	DiFi	MSS	-	-
	Resistant	DiFi_R2	MSS	EGFR	cetuximab
2	Parental	HCA46	MSS	-	-
	Resistant	HCA46_R5	MSS	EGFR	cetuximab
3	Parental	OXCO2	MSI	-	-
	Resistant	OXCO2_R2	MSI	EGFR	cetuximab
4	Parental	LIM1215	MSI	-	-
	Resistant	LIM1215_R2	MSI	EGFR	cetuximab
5	Parental	HT29	MSS	-	-
	Resistant	HT29_R	MSS	BRAF+EGFR	dabrafenib + cetuximab
6	Parental	VACO432	MSI	-	-
	Resistant	VACO432_R	MSI	BRAF+EGFR	dabrafenib + cetuximab
7	Parental	SW837	MSS	-	-
	Resistant	SW837_R	MSS	$KRAS^{G12C}$+EGFR	sotorasib + cetuximab
8	Parental	C106	MSS	-	-
	Resistant	C106_R	MSS	$KRAS^{G12C}$+EGFR	sotorasib + cetuximab
