# Supplementary material for: WEE1 kinase inhibition to overcome acquired resistance to targeted therapies in colorectal cancer
Source: EMBO Mol Med. 2026 May 19;18(6):2322–59. doi: 10.1038/s44321-026-00434-4 (PMC13269805; doi:10.1038/s44321-026-00434-4)
Supplement: Supplementary file 3 — Expanded View Figures [file 44321_2026_434_MOESM3_ESM.pdf]

Expanded View Figures

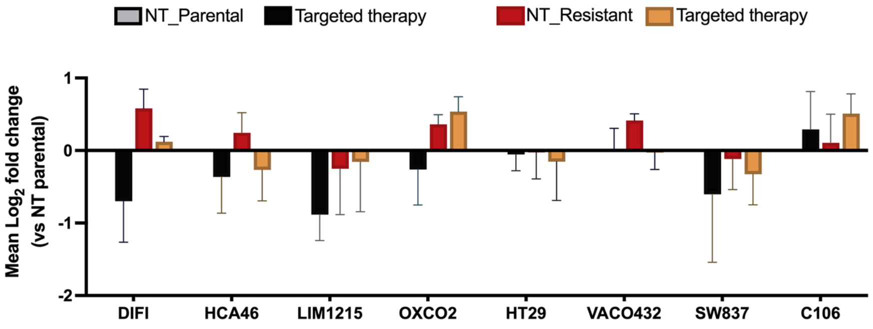

Figure EV1. Biochemical assessment of MAPK activity by phospho-ERK quantification in HT29 and HCA46 pairs post-treatment with targeted therapy.

Quantification of pERK levels normalized to total ERK and Vinculin. Data are presented as mean ± SEM from $n = 3$ independent biological experiments.

 © The Author(s)

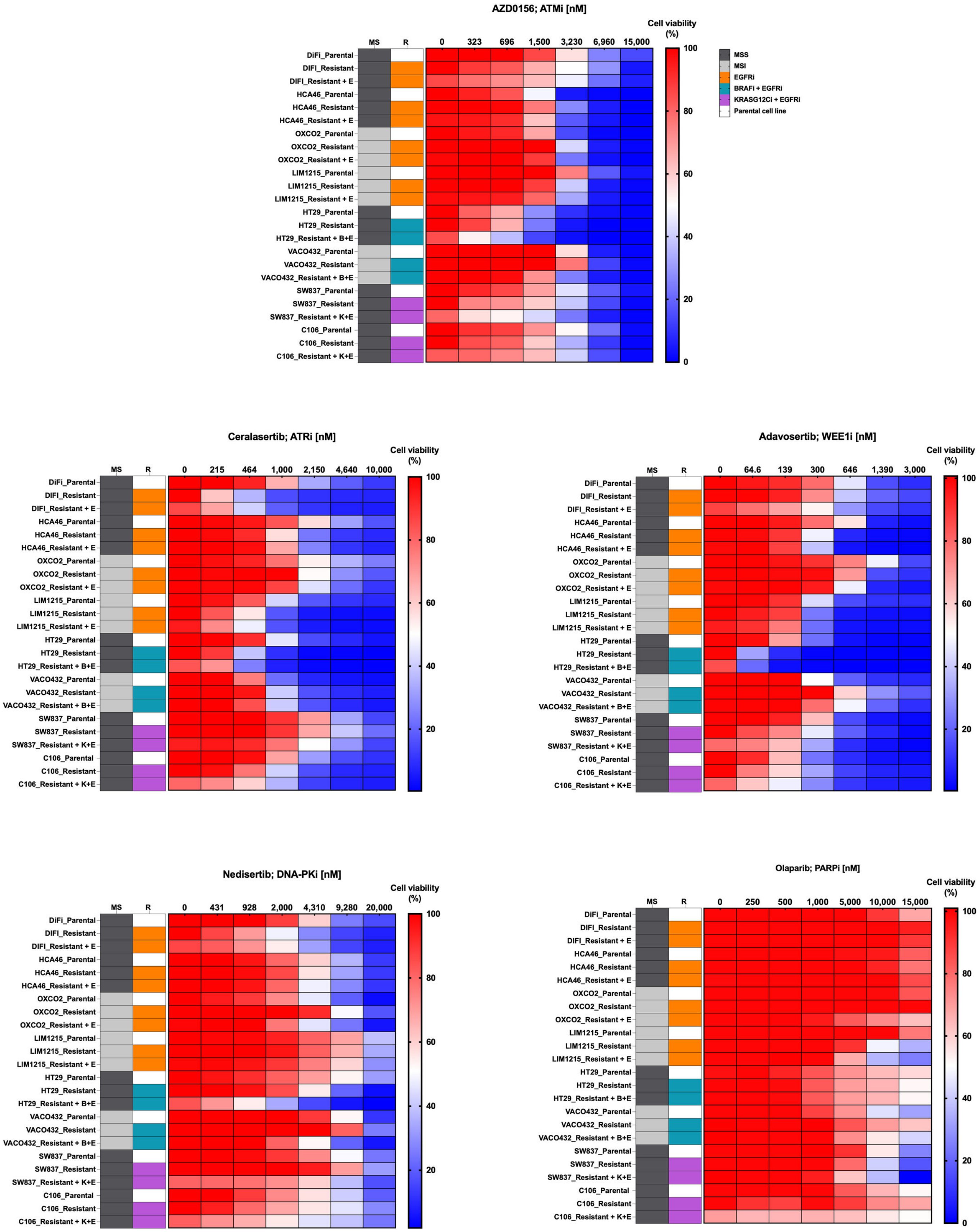
AZD0156; ATMi [nM]
MS
R
0
323
696
1,500
3,230
6,960
15,000
Cell viability (%)
100
80
60
40
20
0
MSS
MSI
EGFRi
BRAFi + EGFRi
KRASG12Ci + EGFRi
Parental cell line
DiFi_Parental
DIFI_Resistant
DIFI_Resistant + E
HCA46_Parental
HCA46_Resistant
HCA46_Resistant + E
OXCO2_Parental
OXCO2_Resistant
OXCO2_Resistant + E
LIM1215_Parental
LIM1215_Resistant
LIM1215_Resistant + E
HT29_Parental
HT29_Resistant
HT29_Resistant + B+E
VACO432_Parental
VACO432_Resistant
VACO432_Resistant + B+E
SW837_Parental
SW837_Resistant
SW837_Resistant + K+E
C106_Parental
C106_Resistant
C106_Resistant + K+E
Ceralasertib; ATRi [nM]
0
215
464
1,000
2,150
4,640
10,000
Adavosertib; WEE1i [nM]
0
64.6
139
300
646
1,390
3,000
Nedisertib; DNA-PKi [nM]
0
431
928
2,000
4,310
9,280
20,000
Olaparib; PARPi [nM]
0
250
500
1,000
5,000
10,000
15,000

Figure EV2. Pharmacological screening of models with acquired resistance to EGFR-based targeted treatments.

Heatmaps depict sensitivity to five different DNA damage response inhibitors (DDRi) across eight CRC cell line pairs with acquired resistance to distinct molecularly targeted therapies. Drug sensitivity was assessed using a 7-day viability assay using CellTiter-Glo® Assay. Parental cell lines were seeded at the following densities (cells per well): DiFi (1×10^4), HCA46 (1.2×10^4), LIM1215 (4×10^3), OXCO2 (1×10^4), HT29 (3×10^3), VACO432 (6×10^3), C106 (1.2×10^4), SW837 (1×10^4). 24 h after seeding cells were treated with DDRi as monotherapy at the indicated range of concentrations: AZD0156 (ATMi, 0–15 μM), ceralasertib (ATRi, 0–10 μM), adavosertib (WEE1i, 0–3 μM), nedisertib (DNA-PKi, 0–20 μM), and olaparib (PARPi, 0–15 μM), whereas cell lines with acquired resistance were treated either with DDRi monotherapy (same range) or in combination with the molecularly targeted therapy to which resistance had developed: E: EGFRi-cetuximab (25 μg/ml) for HCA46, OXCO2, LIM1215 and DiFi; B + E: BRAFi-dabrafenib (4 μM) plus EGFRi-cetuximab (5 μg/ml) for HT29; B + E: BRAFi-dabrafenib (2 μM) plus EGFRi-cetuximab (5 μg/ml) for VACO432; K + E: KRAS-G12Ci (AMG 510, 3 μM) plus EGFRi-cetuximab (15 μg/ml) for SW837; K + E: KRAS-G12Ci (AMG 510, 0.5 μM) plus EGFRi-cetuximab (15 μg/ml) for C106. For each cell line, three independent biological replicates were performed, each with technical triplicates. The first column indicates microsatellite (MS) status: microsatellite stable (MSS, dark gray) or microsatellite instable (MSI, gray). The second column indicates the targeted therapy to which resistance was acquired: EGFRi-cetuximab (E, orange), BRAFi-dabrafenib plus EGFRi-cetuximab (B + E, ochre), or KRAS-G12Ci-(AMG 510) plus EGFRi-cetuximab (K + E, purple). Heatmaps were generated using GraphPad Prism software.

 © The Author(s)

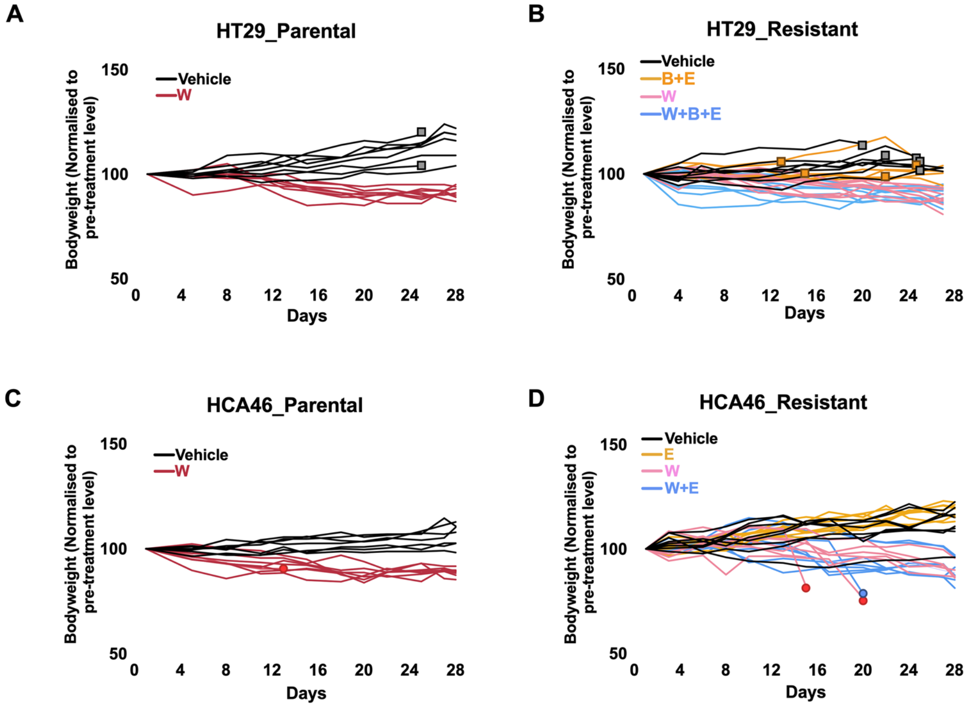

Figure EV3. Drug tolerability in the xenograft experiment.

Ten millions HT29 and HCA46 parental and resistant cells (1:1 mixture with Matrigel) were injected subcutaneously into female NOD-SCID mice (5–9 weeks old). Tumors were measured three times weekly, and once volume reached 100–150 mm³ mice were randomized and treated ($n = 8$ mice per group) twice a week (BIW) intraperitoneally with E: EGFRi-cetuximab (10 mg/kg) and/or twice a day (BID) by oral gavage for W:WEE1i-adavosertib (60 mg/kg) and once a day (QD) by oral gavage for B:BRAFi-dabrafenib (15 mg/kg,). The mean body mass was normalized to the mean value recorded prior to treatment initiation. Circles indicate mice removed from the study due to ≥20% body weight loss. (**A**, **B**) Body weight monitoring during treatment in HT29_Parental and HT29_Resistant cohorts, respectively. (**C**, **D**) Body weight monitoring during treatment in HCA46 parental and HCA46_Resistant cohorts, respectively. W: WEE1i; B: BRAFi; E: EGFRi.

© The Author(s)

A

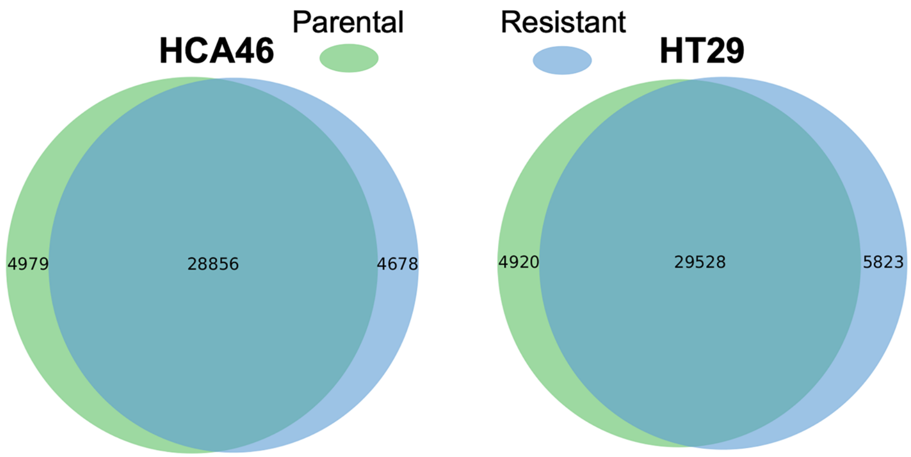

B

HCA46_Resistant

chr	position	ref_nucleotide	var_nucleotide	GeneName	AAchange	variant_type	mutation_type	SnpEff_Annotation	AF_parent	AF_resist	status
chr2	70553195	A	C	TGFA	Leu16Trp	SNP	nonsynonymous	missense_variant	0	0.131	Gained
chr7	55160234	G	A	EGFR	Gly465Glu	SNP	nonsynonymous	missense_variant	0	0.47	Gained
chr14	50156939	G	A	SOS2	Thr410Ile	SNP	nonsynonymous	missense_variant	0.964	0.963	Stable
chr14	50156961	G	A	SOS2	His403Tyr	SNP	nonsynonymous	missense_variant	0.966	0.965	Stable
chr14	50156967	A	G	SOS2	Tyr401His	SNP	nonsynonymous	missense_variant	0.563	0.663	Increased
chr17	39727784	C	G	ERBB2	Pro1170Ala	SNP	nonsynonymous	missense_variant	0.991	0.989	Stable
chr2	211383828	G	T	ERBB4	Asp1238Glu	SNP	nonsynonymous	missense_variant	0.296	0.386	Stable
chr4	109980042	G	A	EGF	Met708Ile	SNP	nonsynonymous	missense_variant	0.554	0.574	Stable
chr4	109993271	A	T	EGF	Glu920Val	SNP	nonsynonymous	missense_variant	0.482	0.537	Stable

HT29_Resistant

chr	position	ref_nucleotide	var_nucleotide	GeneName	AAchange	variant_type	mutation_type	SnpEff_Annotation	AF_parent	AF_resist	Status
chr12	25227337	CC	TT	KRAS	Glu63Lys	Complex	nonsynonymous	missense_variant	0	0.697	Gained
chr14	50156951	G	A	SOS2	Thr406Ile	SNP	nonsynonymous	missense_variant	0	0.048	Gained
chr14	50156939	G	A	SOS2	Thr410Ile	SNP	nonsynonymous	missense_variant	0.929	0.951	Stable
chr14	50156961	G	A	SOS2	His403Tyr	SNP	nonsynonymous	missense_variant	0.932	0.952	Stable
chr17	39723335	A	G	ERBB2	Ile655Val	SNP	nonsynonymous	missense_variant	0.277	0.299	Stable
chr17	39727784	C	G	ERBB2	Pro1170Ala	SNP	nonsynonymous	missense_variant	0.305	0.351	Stable
chr2	211712052	A	C	ERBB4	His374Gln	SNP	nonsynonymous	missense_variant&splice_region_variant	0.435	0.294	Decreased
chr4	74750631	A	T	BTC	Leu124Met	SNP	nonsynonymous	missense_variant	0.649	0.65	Stable
chr4	109980042	G	A	EGF	Met708Ile	SNP	nonsynonymous	missense_variant	0.697	0.624	Stable
chr4	109993271	A	T	EGF	Glu920Val	SNP	nonsynonymous	missense_variant	0.99	0.994	Stable
chr7	55161562	G	A	EGFR	Arg521Lys	SNP	nonsynonymous	missense_variant	0.464	0.579	Increased
chr7	140753336	A	T	BRAF	Val640Glu	SNP	nonsynonymous	missense_variant	0.198	0.286	Increased
chr7	140834757	G	C	BRAF	Thr119Ser	SNP	nonsynonymous	missense_variant	0.706	0.708	Stable

Figure EV4. Analysis of genetic alterations upon acquisition of resistance in HT29 and HCA46 pairs.

(**A**) Venn diagram illustrates the distribution of total somatic variants, depicting the number of variants shared between parental (green) and resistant (blue) cell lines as well as those unique to each. (**B**) Table of EGFR-MAPK pathway somatic nonsynonymous variants identified in resistant cell lines following whole-exome sequencing analysis. For each resistant model (HCA46 and HT29), variants are annotated for genomic position, allelic frequency, gene name and predicted functional impact.

 © The Author(s)

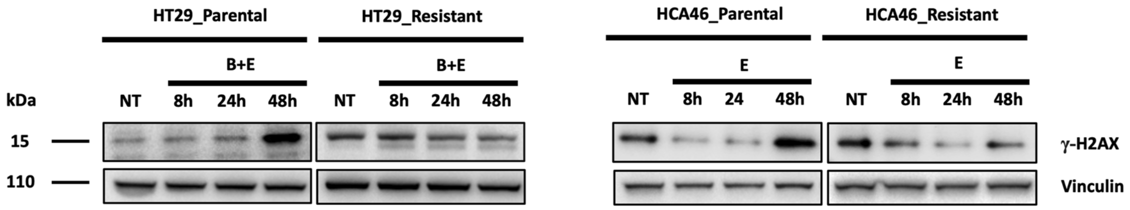

Figure EV5. Biochemical assessment of DNA damage in HT29 and HCA46 pairs post-treatment with targeted therapy.

γ-H2AX protein levels were analyzed by immunoblot as a marker of DNA damage in parental and resistant HT29 and HCA46 cell line pairs under basal conditions or after time-course treatment with targeted therapy for 8, 24, or 48 h. Cells were seeded at 5×10^5 cells per well in 6-well plates on day 0 and treated the following day with E: EGFRi-cetuximab (25 μg/ml) for HCA46 or B + E: BRAFi-dabrafenib (4 μM) plus EGFRi-cetuximab (5 μg/ml) for HT29. Vinculin was used as a loading control in all panels. Images are representative of two biological replicates. Source data are available online for this figure

© The Author(s)

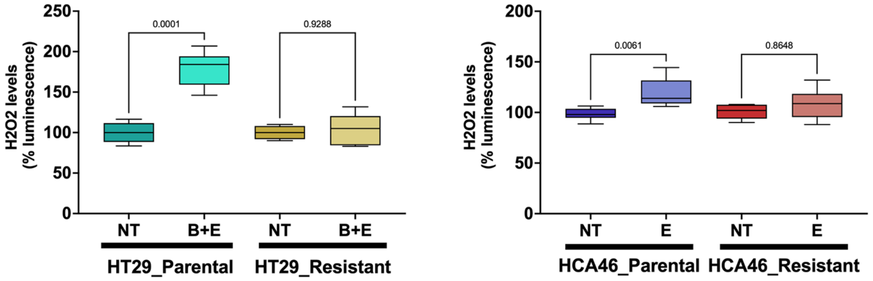

Figure EV6. Assessment of reactive oxygen species following targeted therapy treatment.

Reactive oxygen species evaluation in HT29 and HCA46 pairs in basal conditions and after treatment with targeted therapy. Cells were seeded on day 0 and treated from day 1 with targeted therapies E: EGFRi-cetuximab (25 µg/ml), while HT29 cells were treated with B + E: BRAFi-dabrafenib (4 µM) in combination with E: EGFRi-cetuximab (5 µg/ml). After 48 h, reactive oxygen species were quantified using the ROS-Glo™ H2O2 Assay. Luminescence values were normalized to untreated controls. Data are presented as mean ± SEM from $n = 3$ independent biological experiments. In the box plots the center line represents the median, the box bounds represent the 25th and 75th percentiles (interquartile range), and the whiskers extend from the minimum to the maximum value. Individual data points are overlaid. Statistical significance was assessed using a two-tailed Mann–Whitney U test.

 © The Author(s)

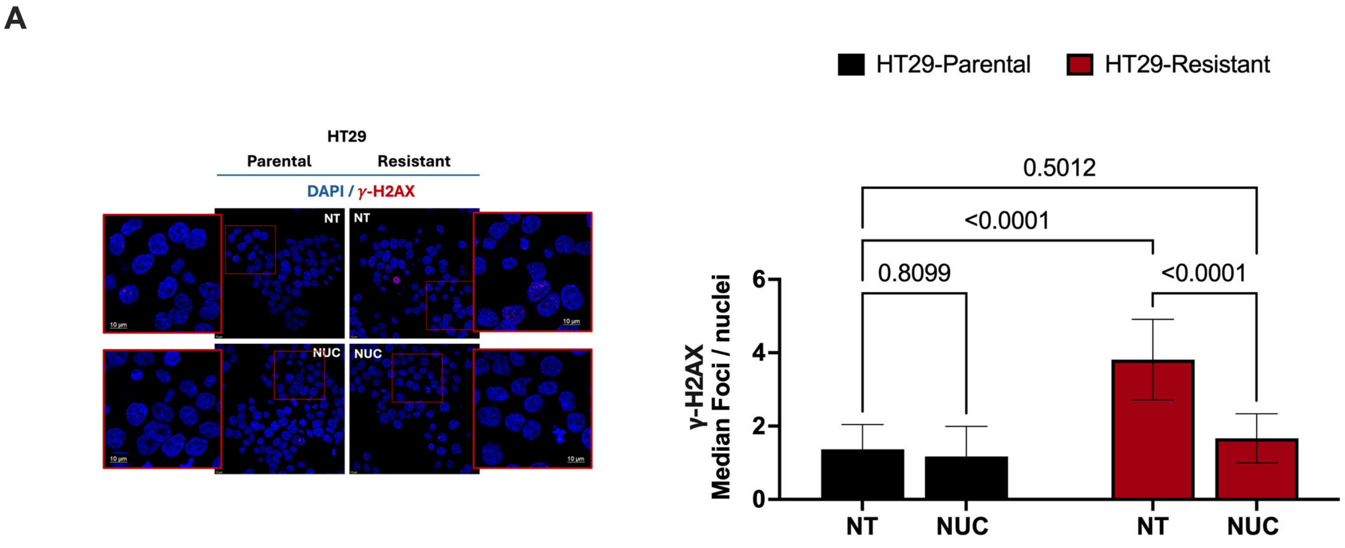

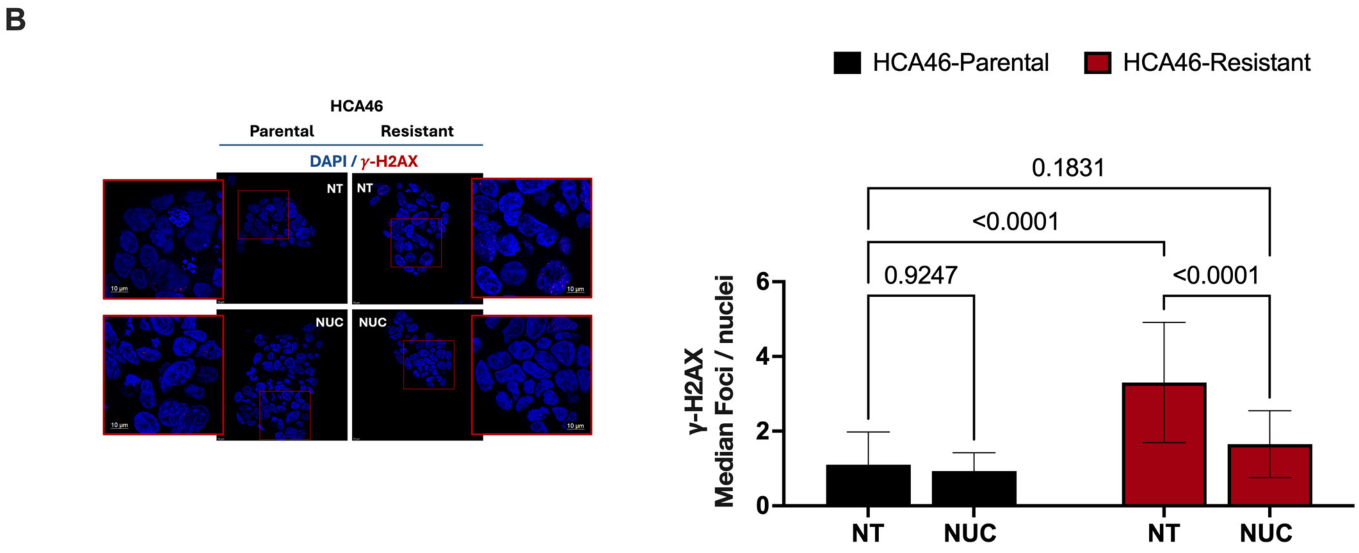

Figure EV7. Rescue of replication of DNA damage by nucleoside supplementation in CRC cells with acquired resistance to anti-EGFR therapies.

γ-H2AX immunofluorescence analysis in parental and resistant HT29 (**A**) and HCA46 (**B**) cells under basal conditions or after nucleoside supplementation (NUC). Cells were seeded on coverslips and supplemented the following day. Twenty-four hours post-supplementation, cells were fixed and processed for γ-H2AX immunostaining. Nuclei were counterstained with DAPI. Data are presented as mean ± SEM from $n = 3$ independent biological experiments (at least 5 images were quantified, counting for a total of 100 nuclei/biological experiment). Images were acquired using a Leica Stellaris confocal microscope equipped with a 63× oil-immersion objective (NA 1.4) under identical acquisition settings for all conditions. Scale bar, 10 µm. Statistical significance was assessed using one-way ANOVA with Tukey's multiple-comparisons test.

© The Author(s)

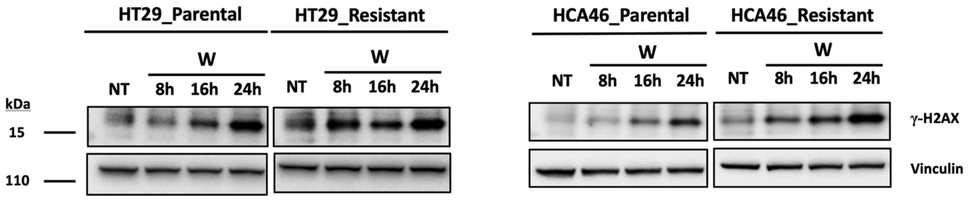

Figure EV8. Biochemical assessment of DNA damage in HT29 and HCA46 pairs upon treatment with WEE1i.

γ-H2AX protein levels were analyzed by immunoblot as a marker of DNA damage in parental and resistant HT29 and HCA46 cell line pairs under basal conditions or after time-course treatment with the WEE1 inhibitor adavosertib (300 nM) for 8, 16, or 24 h. Cells were seeded at 5×10^5 cells per well in 6-well plates on day 0 and treated the following day. Vinculin was used as a loading control in all panels. Images are representative of two biological replicates.

 © The Author(s)

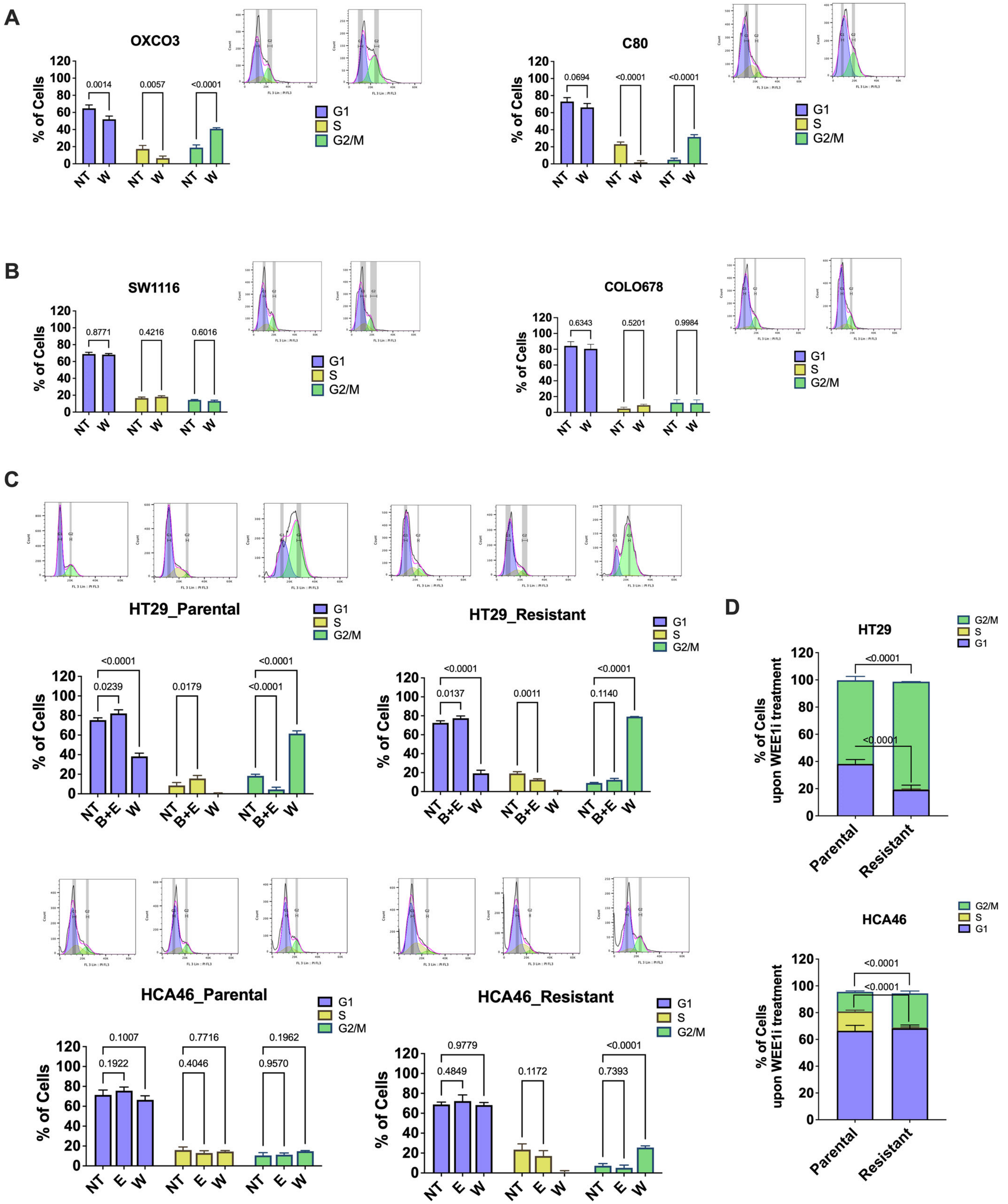
A
OXCO3
C80
% of Cells
0.0014
0.0057
<0.0001
0.0694
<0.0001
<0.0001
NT W NT W NT W
G1
S
G2/M
B
SW1116
COLO678
0.8771
0.4216
0.6016
0.6343
0.5201
0.9984
C
HT29_Parental
HT29_Resistant
<0.0001
0.0239
0.0179
<0.0001
0.0137
0.0011
0.1140
NT B+E W
HCA46_Parental
HCA46_Resistant
0.1007
0.1922
0.7716
0.4046
0.1962
0.9570
0.9779
0.4849
0.1172
0.7393
NT E W
D
HT29
HCA46
% of Cells upon WEE1i treatment
Parental
Resistant

Figure EV9. Effect of WEE1 Inhibition on the cell cycle in CRC cells.

Cell cycle profiles analyzed in control models including WEE1 inhibitor-sensitive (OXCO3, C80; (**A**)) and WEE1 inhibitor-resistant (SW1116, COLO678; (**B**)) cell lines, as well as HT29 and HCA46 parental and resistant pairs (**C**, **D**). Cells were treated with adavosertib (300 nM) for 24 h. For HT29 and HCA46 pairs, additional conditions included E: EGFRi-cetuximab (25 μg/ml) for HCA46 or B + E: BRAFi-dabrafenib (4 μM) in in combination with EGFRi-cetuximab (5 μg/ml). DNA content was measured by propidium iodide staining followed by flow cytometry, and the percentage of cells in G1, S, and G2/M phases was quantified using FlowJo software. Data are presented as mean ± SEM from $n = 3$ independent biological experiments. Statistical significance was assessed using two-way ANOVA with Tukey's multiple-comparisons test.

 © The Author(s)

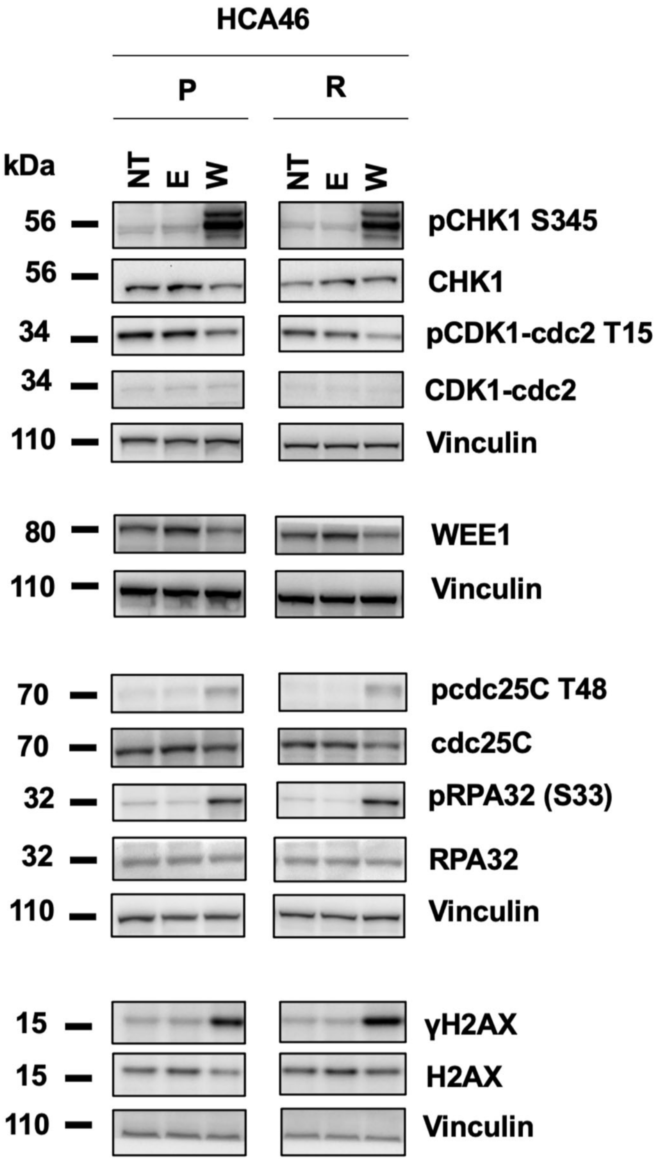

Figure EV10. WEE1 inhibition induces replication stress and DNA damage signaling in parental and resistant HCA46 cells.

Immunoblot analysis of markers of replication stress, cell cycle and DNA damage response players in in parental (P) and resistant (R) HCA46 cells seeded at 5×10^5 cells per well in 6-well plates and treated after 24 h with EGFRi-cetuximab (25 µg/ml, 24 h) or W-WEE1i-adavosertib (300 nM, 24 h). Total protein levels are shown as controls, and vinculin was used as a loading control. Representative immunoblots from two independent biological experiments are shown. Source data are available online for this figure.

© The Author(s)

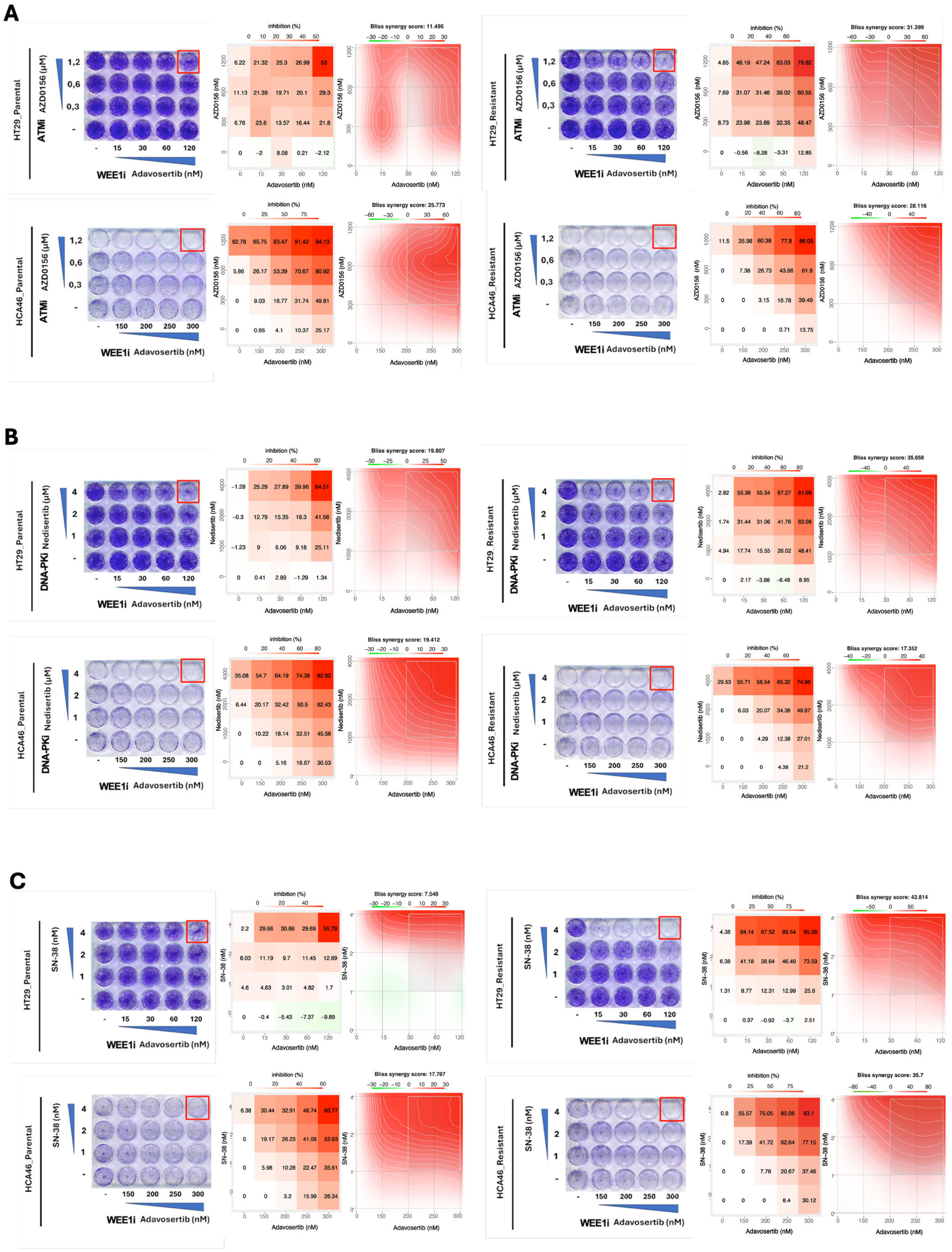
A
B
C
HT29_Parental
HT29_Resistant
HCA46_Parental
HCA46_Resistant
ATMi AZD0156 (µM)
DNA-PKi Nedisertib (µM)
SN-38 (nM)
WEE1i Adavosertib (nM)
inhibition (%)
Bliss synergy score: 11.495
Bliss synergy score: 31.399
Bliss synergy score: 25.773
Bliss synergy score: 28.116
Bliss synergy score: 19.807
Bliss synergy score: 35.658
Bliss synergy score: 19.412
Bliss synergy score: 17.352
Bliss synergy score: 7.548
Bliss synergy score: 42.814
Bliss synergy score: 17.787
Bliss synergy score: 35.7
Adavosertib (nM)
AZD0156 (nM)
Nedisertib (nM)
SN-38 (nM)

Figure EV11. Evaluation of synergism by combining WEE1i with different DDRi (ATM, DNA-PK) or chemotherapeutic agents (SN-38).

The indicated parental and resistant cells were seeded on day 0 and treated the following day with combinations of sublethal concentrations of a W:WEE1-adavosertib (15–120 nM) for HT29 and (150–300 nM) for HCA46 together with ATMi-AZD0156 (300–1200 nM), DNA-Pki Nedisertib (1–4 µM), or SN-38-active compound of irinotecan (1–4 nM), arranged in a dose–response matrix for synergy score calculation (see Methods). After 6 days of treatment, cells were fixed and stained with crystal violet. Quantification was performed by measuring the absorbance of the dye dissolved in acetic acid. Panel (**A**) shows combinations of WEE1 inhibitor with ATMi; panel (**B**) shows combinations with DNA-PKi; panel (**C**) shows combinations with SN-38. Images are representative of two biological replicates.

© The Author(s)

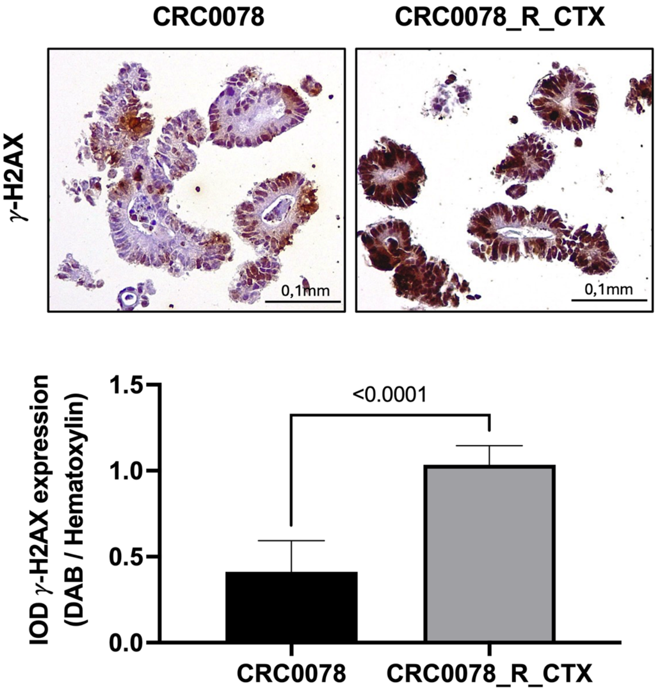

Figure EV12. γ-H2AX expression analysis in patient-derived xenograft FFPE.

Representative immunohistochemistry images showing γ-H2AX staining as a marker of DNA damage in patient-derived organoids under basal conditions. Organoids were cultured embedded in matrix until reaching adequate size, collected, formalin fixed and paraffin-embedded (FFPE), sectioned, and stained. Scale bar, 0.1 mm. Immunoreactivity was quantified with the NIH Image J software using the color-deconvolution plug-in that has a built-in vector for separating hematoxylin (H) and diaminobenzidine (DAB) stainings. After color deconvolution DAB images and hematoxylin are processed separately. Data are presented as mean ± SD from $n = 10$ images/condition. Statistical significance was assessed using a two-tailed Mann–Whitney U test.

 © The Author(s)
