# Supplementary material for: WEE1 kinase inhibition to overcome acquired resistance to targeted therapies in colorectal cancer
Source: EMBO Mol Med. 2026 May 19;18(6):2322–59. doi: 10.1038/s44321-026-00434-4 (PMC13269805; doi:10.1038/s44321-026-00434-4)
Supplement: Supplementary file 2 — Peer Review File [file 44321_2026_434_MOESM2_ESM.pdf]

EMBO Molecular Medicine

WEE1 kinase inhibition to overcome acquired resistance to targeted therapies in colorectal cancer

Kristi Buzo, Laura Bizzozero, Marilena Lentini, Alena Opattova, Beatriz Hernandez Suarez, Michael Torres, Giulia Chiabotto, Clelia Nisticò, Giada De Lazzari, Gaia Grasso, Elisa Mariella, Erika Durinikova, Eugenia Zanella, Michael Linnebacher, Andrea Sartore Bianchi, Salvatore Siena, Livio Trusolino, Alberto Bardelli, and Sabrina Arena

Corresponding author: Sabrina Arena (sabrina.arena@unito.it)

Review Timeline:

Submission Date:	10th Oct 25
Editorial Decision:	31st Oct 25
Revision Received:	27th Feb 26
Editorial Decision:	27th Mar 26
Revision Received:	9th Apr 26
Accepted:	17th Apr 26

Editor: Lise Roth

Transaction Report:

(Note: Please note that the manuscript was previously reviewed at another journal and the reports were taken into account in the decision making process at EMBO Molecular Medicine. Since the original reviews are not subject to EMBO Press' transparent review process policy, the reports and author response cannot be published.
With the exception of the correction of typographical or spelling errors that could be a source of ambiguity, letters and reports are not edited. Depending on transfer agreements, referee reports obtained elsewhere may or may not be included in this compilation. Referee reports are anonymous unless the Referee chooses to sign their reports.)

31st Oct 2025

Dear Prof. Arena,

Thank you for the submission of your manuscript to EMBO Molecular Medicine, following its rejection after peer-review by another journal.
While the initial referees raised concerns related to the mechanistic insights and unclear translational advance, we found your revised manuscript within the scope of EMBO Molecular Medicine, and sent it to a single reviewer for evaluation, together with your point-by-point rebuttal letter to the initial referees' concerns. We have now received the enclosed report from this reviewer, who supports publication pending minor revisions (see below). We would therefore welcome the submission of your revised manuscript in accordance with this referee's recommendations.

EMBO Molecular Medicine encourages a single round of revision only and therefore, acceptance or rejection of the manuscript will depend on the completeness of your responses included in the next, final version of the manuscript. For this reason, and to save you from any frustrations in the end, I would strongly advise against returning an incomplete revision.

When submitting your revised manuscript, please carefully review the instructions that follow below. We perform an initial quality control of all revised manuscripts before re-review; failure to include requested items will delay the evaluation of your revision.

We require:

1) A .docx formatted version of the manuscript text (including legends for main figures, EV figures and tables). Please make sure that the changes are highlighted to be clearly visible.

2) Individual production quality figure files as .eps, .tif, .jpg (one file per figure). For guidance, download the 'Figure Guide PDF' (https://www.embopress.org/page/journal/17574684/authorguide#figureformat).

3) At EMBO Press we ask authors to provide source data for the main manuscript figures. You will receive a separate email with instructions for providing source data with your revised manuscript, including how to upload and organize the files.

Additional information on source data and instruction on how to label the files are available

4) A .docx formatted letter INCLUDING the reviewers' reports and your detailed point-by-point responses to their comments. As part of the EMBO Press transparent editorial process, the point-by-point response is part of the Review Process File (RPF), which will be published alongside your paper.

5) A complete author checklist, which you can download from our author guidelines (https://www.embopress.org/page/journal/17574684/authorguide#submissionofrevisions). Please insert information in the checklist that is also reflected in the manuscript. The completed author checklist will also be part of the RPF.

6) All Materials and Methods need to be described in the main text using our 'Structured Methods' format. According to this format, the Methods section includes a Reagents and Tools Table (listing key reagents, experimental models, software and relevant equipment and including their sources and relevant identifiers) followed by a Methods and Protocols section describing the methods, ideally using a step-by-step protocol format. The aim is to facilitate adoption of the methodologies across labs. Please download and fill our Reagents and Tools Table template (.docx), which you can find in our author guidelines: https://www.embopress.org/page/journal/14693178/authorguide#structuredmethods.
When submitting your revised manuscript, please do not include the Reagents and Tools Table in the Methods section of the manuscript but upload it as a separate file choosing the file type "Reagent Table".

7) Please note that all corresponding authors are required to supply an ORCID ID for their name upon submission of a revised manuscript.

8) It is mandatory to include a 'Data Availability' section after the Materials and Methods. Before submitting your revision, primary datasets produced in this study need to be deposited in an appropriate public database, and the accession numbers and database listed under 'Data Availability'. Please remember to provide a reviewer password if the datasets are not yet public (see https://www.embopress.org/page/journal/17574684/authorguide#dataavailability).

In case you have no data that requires deposition in a public database, please state so in this section. Note that the Data Availability Section is restricted to new primary data that are part of this study.

9) For data quantification: please specify the name of the statistical test used to generate error bars and P values, the number

(n) of independent experiments (specify technical or biological replicates) underlying each data point and the test used to calculate p-values in each figure legend. The figure legends should contain a basic description of n, P and the test applied. Graphs must include a description of the bars and the error bars (s.d., s.e.m.). Please provide exact p values.

10) Our journal encourages inclusion of *data citations in the reference list* to directly cite datasets that were re-used and obtained from public databases. Data citations in the article text are distinct from normal bibliographical citations and should directly link to the database records from which the data can be accessed. In the main text, data citations are formatted as follows: "Data ref: Smith et al, 2001" or "Data ref: NCBI Sequence Read Archive PRJNA342805, 2017". In the Reference list, data citations must be labeled with "[DATASET]". A data reference must provide the database name, accession number/identifiers and a resolvable link to the landing page from which the data can be accessed at the end of the reference. Further instructions are available at .

11) We replaced Supplementary Information with Expanded View (EV) Figures and Tables that are collapsible/expandable online. EV Figures should be cited as 'Figure EV1, Figure EV2" etc... in the text and their respective legends should be included in the main text after the legends of regular figures.
- For the figures that you do NOT wish to display as Expanded View figures, they should be bundled together with their legends in a single PDF file called *Appendix*, which should start with a short Table of Content. Appendix figures should be referred to in the main text as: "Appendix Figure S1, Appendix Figure S2" etc.
- Additional Tables/Datasets should be labeled and referred to as Table EV1, Dataset EV1, etc. Legends have to be provided in a separate tab in case of .xls files. Alternatively, the legend can be supplied as a separate text file (README) and zipped together with the Table/Dataset file.
See detailed instructions here:

12) The paper explained: EMBO Molecular Medicine articles are accompanied by a summary of the articles to emphasize the major findings in the paper and their medical implications for the non-specialist reader. Please provide a draft summary of your article highlighting
- the medical issue you are addressing,
- the results obtained and
- their clinical impact.
This may be edited to ensure that readers understand the significance and context of the research. Please refer to any of our published articles for an example.

13) Author contributions: CRediT has replaced the traditional author contributions section because it offers a systematic machine readable author contributions format that allows for more effective research assessment. Please remove the Authors Contributions from the manuscript and use the free text boxes beneath each contributing author's name in our system to add specific details on the author's contribution. More information is available in our guide to authors.

14) Disclosure statement and competing interests: We updated our journal's competing interests policy in January 2022 and request authors to consider both actual and perceived competing interests. Please review the policy https://www.embopress.org/competing-interests and update your competing interests if necessary.

15) Every published paper now includes a 'Synopsis' to further enhance discoverability. Synopses are displayed on the journal webpage and are freely accessible to all readers. They include a short stand first (maximum of 300 characters, including space) as well as 2-5 one-sentences bullet points that summarizes the paper. Please write the bullet points to summarize the key NEW findings. They should be designed to be complementary to the abstract - i.e. not repeat the same text. We encourage inclusion of key acronyms and quantitative information (maximum of 30 words / bullet point). Please use the passive voice. Please attach these in a separate file or send them by email, we will incorporate them accordingly.

Please also suggest a visual abstract to illustrate your article as a PNG file 550 px wide x 300-600 px high. A cropped portion of this image will serve as thumbnail for the table of content on our webpage.

16) As part of the EMBO Publications transparent editorial process initiative (see our Editorial at http://embomolmed.embopress.org/content/2/9/329), EMBO Molecular Medicine will publish online a Review Process File (RPF) to accompany accepted manuscripts.
In the event of acceptance, this file will be published in conjunction with your paper and will include the anonymous referee reports, your point-by-point response and all pertinent correspondence relating to the manuscript. Let us know whether you agree with the publication of the RPF and as here, if you want to remove or not any figures from it prior to publication.
Please note that the Authors checklist will be published at the end of the RPF.

EMBO Molecular Medicine has a "scooping protection" policy, whereby similar findings that are published by others during review or revision are not a criterion for rejection. Should you decide to submit a revised version, I do ask that you get in touch after three months if you have not completed it, to update us on the status.

I look forward to receiving your revised manuscript.

Yours sincerely,

Lise Roth

Lise Roth, PhD
Senior Editor
EMBO Molecular Medicine

***** Reviewer's comments *****

Referee #1 (Remarks for Author):

WEE1 kinase inhibition to overcome acquired resistance to targeted therapies in colorectal cancer

In this study the authors have explored the use of DDR inhibitors to target colorectal cancers which have developed acquired resistance to EGFR blockade. To address this the authors have developed cell lines which have acquired resistance to a combination of BRAF inhibitors (dabrafenib) and anti-EGFR monoclonal antibody (Cetuximab) in HT29 (microsatellite stability) and VACO432 (microsatellite instability). Further they have developed cell lines derived from the C106 and SW837 which are resistant to combination of KRASG12C inhibitor (AMG510) and anti-EGFR (cetuximab). They then examined the impact of PARPi, DNA-PKi, ATMi ATRi and WEE1i. They show that of these, WEE1 inhibition resulted in a striking reduction in viability in the majority of these resistant cell lines both in vitro and in vivo (xenografts and organoids).
In terms of mechanism they show that WEE1i addition is associated with significant levels of DNA damage (Comet; gH2AX); and altered DNA replication kinetics (reduced CldU and IdU ratios and nucleotide imbalance). RAD51 foci are significantly reduced following WEE1 inhibition in both resistant cell lines. They also explore combining WEE1i with other agents, and observe robust synergy with SN-38.

General comments:
the manuscript is very well conceived, identifying an important problem and indeed a potential solution to acquired resistance to EGFR blockade. The manuscript is well written and the data are generally supportive of the conclusions drawn. The authors have identified a novel niche in a busy area as while there are numerous studies examining the role of DDR /RS inhibitors, little emphasis has been put on resistant cell lines, which is more around MAPK than EGFR based resistance.

The data does contain some curious observations however, which would benefit from further analysis:

1. 'To corroborate these findings, we next evaluated nucleotide metabolism, a key contributor to replication stress, and found that both resistant HT29 and HCA46 cells exhibited a greater imbalance in nucleotide pools between purines and pyrimidines (Figure 4C and D)'
Can you perform a rescue assay/titration of the nucleotides to demonstrate that nucleotide imbalance is responsible for the stalled replication forks?

2. Curiously in the p53 null HCA46 parental cell line cells were arrested in G1 after WEE1 inhibition was maintained. This is surprising! Could the authors explain this? Indeed are the cells possibly entering and arresting in non-replicating S-phase following WEE1 inhibition which has been shown elsewhere?

3. They also note an increase in RAD51 foci in resistant HT29 cells rather than the parental cells at baseline. However, RAD51 foci are significantly reduced following WEE1 inhibition in both resistant cell lines, suggesting that HR is downregulated in resisistant cells? However, it this is a bad comparison as these cells are treated for different periods of time -24H (for E, B+E or HU) compared to 8h (for WEE1i). This needs to be addressed. As it is misleading to have these in the same histogram. Also a timecourse should be performed comparing the impact of WEE1i over time in parental and resistant cell lines.

4. The authors demonstrate robust synergy with WEE1i and SN-38. Could they and found a robust synergy of the two drugs.

However, this is barely mentioned in the text and explanation of the results provided. Could the authors put further details in and perhaps discuss what they think is going on here?

Typos:
116/117: the crucial role of WEE1 in ensuring e proper repair
P223: with respect to their parental counterpart
250: although to different extents
268: effectors, such as RAD51
355: might drives a resistance state (no s).
P388: cancers including recurrent..... (replace as with including)
403: The identification of biomarkers predicting response to WEE1i remains an unmet need.

Point-by-point response for Buzo et al., EMM-2025-22719

******* Reviewer's comments *******

Referee #1 (Remarks for Author):

WEE1 kinase inhibition to overcome acquired resistance to targeted therapies in colorectal cancer

In this study the authors have explored the use of DDR inhibitors to target colorectal cancers which have developed acquired resistance to EGFR blockade. To address this the authors have developed cell lines which have acquired resistance to a combination of BRAF inhibitors (dabrafenib) and anti-EGFR monoclonal antibody (Cetuximab) in HT29 (microsatellite stability) and VACO432 (microsatellite instability). Further they have developed cell lines derived from the C106 and SW837 which are resistant to combination of KRASG12C inhibitor (AMG510) and anti-EGFR (cetuximab). They then examined the impact of PARPi, DNA-PKi, ATMi ATRi and WEE1i. They show that of these, WEE1 inhibition resulted in a striking reduction in viability in the majority of these resistant cell lines both in vitro and in vivo (xenografts and organoids).
In terms of mechanism they show that WEE1i addition is associated with significant levels of DNA damage (Comet; gH2AX); and altered DNA replication kinetics (reduced CldU and IdU ratios and nucleotide imbalance). RAD51 foci are significantly reduced following WEE1 inhibition in both resistant cell lines. They also explore combining WEE1i with other agents, and observe robust synergy with SN-38.

General comments:
the manuscript is very well conceived, identifying an important problem and indeed a potential solution to acquired resistance to EGFR blockade. The manuscript is well written and the data are generally supportive of the conclusions drawn. The authors have identified a novel niche in a busy area as while there are numerous studies examining the role of DDR /RS inhibitors, little emphasis has been put on resistant cell lines, which is more around MAPK than EGFR based resistance.

R. We thank the reviewer for his/her positive and encouraging assessment of our work. We are pleased that he/she considers our manuscript to be well-conceived, clearly written, and relevant to a significant clinical issue. We particularly appreciate the recognition of our focus on acquired resistance to EGFR blockade, an area where DNA damage response (DDR) and replication stress (RS)–targeting strategies have been comparatively underexplored relative to MAPK-driven resistance mechanisms. Our study aimed to fill this gap by generating and analyzing resistant models across various genomic backgrounds (MSS, MSI, BRAF-mutant, and KRAS-mutant) and by systematically testing multiple DDR/RS inhibitors. We are glad the reviewer finds our identification of WEE1 inhibition as a potential therapeutic vulnerability to be compelling and well-supported by both mechanistic and functional

data, including induction of DNA damage, disruption of replication dynamics, suppression of RAD51 foci formation, and robust synergy with SN-38.

The data does contain some curious observations, however, which would benefit from further analysis:

1. 'To corroborate these findings, we next evaluated nucleotide metabolism, a key contributor to replication stress, and found that both resistant HT29 and HCA46 cells exhibited a greater imbalance in nucleotide pools between purines and pyrimidines (Figure 4C and D)'
Can you perform a rescue assay/titration of the nucleotides to demonstrate that nucleotide imbalance is responsible for the stalled replication forks?

R. We thank the reviewer for this insightful suggestion. To directly assess whether nucleotide imbalance contributes to the elevated basal replication stress observed in the acquired-resistant models, we performed a DNA fiber assay following exogenous nucleoside supplementation (NUC). This rescue experiment was designed to determine whether restoration of nucleotide pool balance could alleviate replication fork stalling. Consistent with the hypothesis that altered nucleotide metabolism underlies the replication defects, nucleoside supplementation significantly restored replication fork progression in resistant HT29 and HCA46 cells. Specifically, supplementation increased replication tract length and reduced the frequency of stalled forks, leading to replication dynamics similar to the parental model (Rebuttal Figure 1A-C). We have now included this new data in the manuscript as Figure 5F–H.

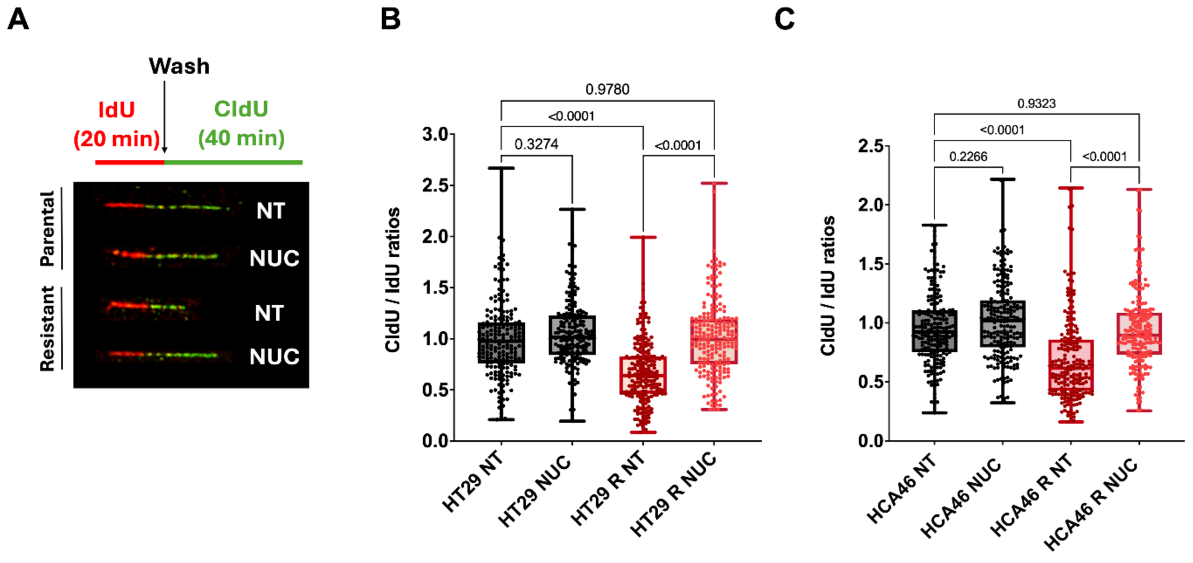

Rebuttal Figure 1(Figure 5F-G-H in the revised manuscript) Rescue of replication stress by nucleoside supplementation in CRC cells with acquired resistance to anti-EGFR therapies.
(A) Schematic overview of the DNA fiber assay showing sequential labeling with IdU (25 µM, 20 min) followed by CldU (250 µM, 40 min). Representative fiber images are shown for basal conditions and following nucleoside supplementation.

(B, C) Quantification of replication fork progression in parental and resistant HT29 (B) and HCA46 (C) cells cultured under basal conditions or supplemented with a nucleoside mixture (EmbryoMax® Nucleoside Mix, 100× stock; Merck/Millipore). Cells were seeded at 2 × 10^5 cells per well in 6-well plates and supplemented the following day under drug-free conditions prior to DNA fiber labeling. Replication dynamics were assessed by measuring the CldU/IdU tract length ratio (with IdU values normalized to 2× to account for pulse duration differences). For each condition, a total of n = 200 replication tracts were analyzed from two independent biological replicates. Statistical significance was determined using a two-tailed Mann–Whitney U test.

In addition, to further substantiate the mechanistic link between replication stress and DNA damage, we assessed RS-associated DNA damage by quantifying γH2AX levels following nucleoside supplementation. γH2AX serves as a well-established marker of endogenous DNA damage arising from replication stress arising under conditions of insufficient or imbalanced nucleotide pools.
Parental and acquired-resistant HT29 and HCA46 cells were cultured in complete medium with or without a balanced nucleoside supplement (EmbryoMax Nucleosides, 100× stock) for 24 h prior to analysis. As shown in the Rebuttal Figure 2, nucleoside supplementation significantly reduced basal γH2AX levels in the acquired-resistant cells, whereas parental cells were largely unaffected.
These findings indicate that the restoration of nucleotide availability and pool balance alleviates endogenous DNA damage in resistant cells, supporting a functional link between altered nucleotide metabolism and elevated basal replication stress. We have now included this new data in the manuscript as Figure EV7A, B.

A

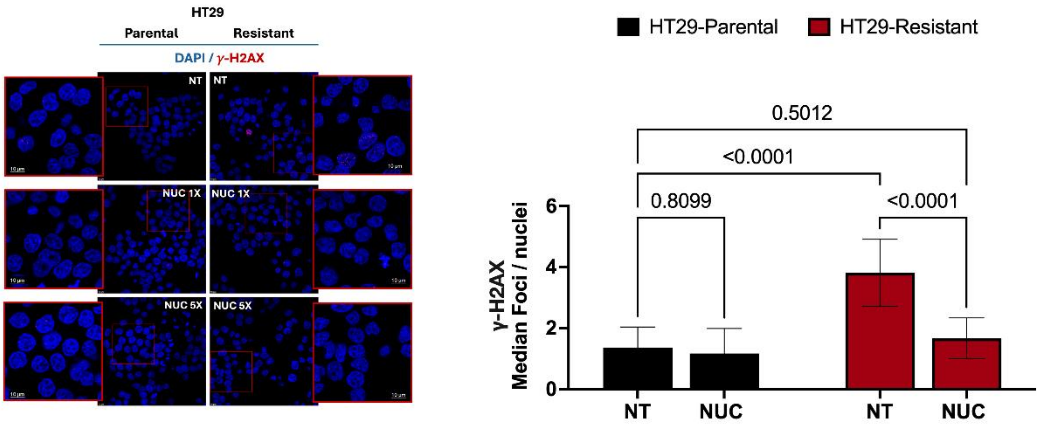

B

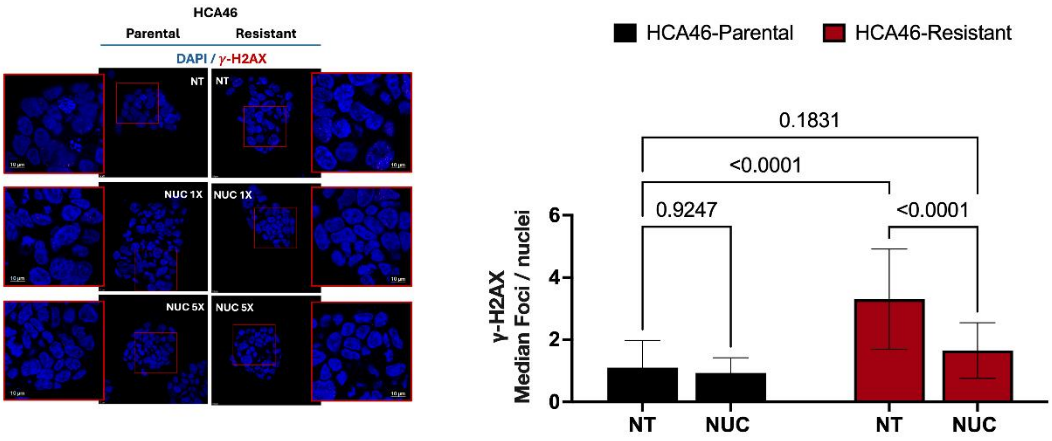

Rebuttal Figure 2 (Figure EV7A, B in the revised manuscript) Rescue of DNA damage following nucleoside supplementation in CRC cells with acquired resistance to anti-EGFR therapies.

(A,B) γH2AX immunofluorescence analysis in parental and resistant HT29 (A) and HCA46 (B) cells under basal conditions or after nucleoside supplementation (NUC). Cells were seeded on coverslips and supplemented the following day. Twenty-four hours post-supplementation, cells were fixed and processed for γH2AX immunostaining. Nuclei were counterstained with DAPI. Data represent ≥600 nuclei from n=3 independent biological experiments. Images were acquired using a Leica Stellaris confocal microscope equipped with a 63× oil-immersion objective (NA 1.4) under identical acquisition settings for all conditions. Scale bar, 10 μm. Statistical significance was assessed using one-way ANOVA with Tukey's multiple-comparisons test.

2. Curiously in the p53 null HCA46 parental cell line cells were arrested in G1 after WEE1 inhibition was maintained. This is surprising! Could the authors explain this? Indeed are the cells possibly entering and arresting in non-

replicating S-phase following WEE1 inhibition which has been shown elsewhere?

R. We thank the reviewer for this insightful comment. We agree that, given the p53-null status of HCA46, a canonical p53-dependent G1 checkpoint arrest would not be expected. Even though there is evidence in other colorectal cell lines regarding a G1-independent block (Shen, G., Xu, C., Chen, C., Hebbar, V., & Kong, A. N. (2006). p53-independent G1 cell cycle arrest of human colon carcinoma cells HT-29 by sulforaphane is associated with induction of p21CIP1 and inhibition of expression of cyclin D1. Cancer chemotherapy and pharmacology, 57(3), 317–327. https://doi.org/10.1007/s00280-005-0050-3; Matsuda T., Kato T., Kiyotani K., Tarhan Y. Emre, Saloura V., Chung S., Ueda K., Nakamura Y., Park J. p53-independent p21 induction by MELK inhibition. Oncotarget. 2017; 8: 57938-57947), to our knowledge this phenomenon has not been specifically described in HCA46 cells.
Our data suggest that the population accumulating in "G1" after WEE1 inhibition does not represent a canonical G1 checkpoint arrest, but rather a failure to enter S-phase due to overwhelming replication stress.
Specifically, following WEE1i treatment, HCA46 cells exhibit marked activation of replication stress and DNA damage signalling pathways, including increased pCHK1, pRPA and γH2AX, consistent with extensive replication fork dysfunction occurring shortly after attempted S-phase entry (Rebuttal figure 3A). In parallel, we observe activation of CDC25C (T48) and a reduction in inhibitory pCDK1-Y15 (Rebuttal figure 3B), consistent with the expected biochemical effect of WEE1 inhibition and indicative of premature CDK activation.
Together, these findings support a model in which WEE1 inhibition forces p53-deficient HCA46 cells into unscheduled or abortive S-phase entry, resulting in rapid accumulation of replication-associated DNA damage.
We have clarified this point in the revised manuscript to better reflect the underlying biology in the section "WEE1 inhibition triggers higher levels of DNA damage in tumor cells that have developed acquired resistance to EGFR blockade" (line 295-303 "In HCA46-resistant model, a higher percentage of cells in the G2/M phase was observed after treatment with WEE1i (Fig. EV9D). Yet a block in G1 after WEE1i was also observed in both HCA46 parental and resistant cells. Although HCA46 cells accumulate in the G1 gate following WEE1 inhibition, this is unlikely to represent a canonical G1 arrest, as these cells are p53-null (Kramer *et al.*, 2016)*. Instead, strong induction of replication-stress markers (pCHK1, pRPA and γH2AX) (Fig. EV10A) followed by activation of CDC25C (T48) and reduction of inhibitory pCDK1-Y15 (Figure EV10B) is observed and suggest premature CDK activation and entry into non-productive S-phase, resulting in replication collapse.*

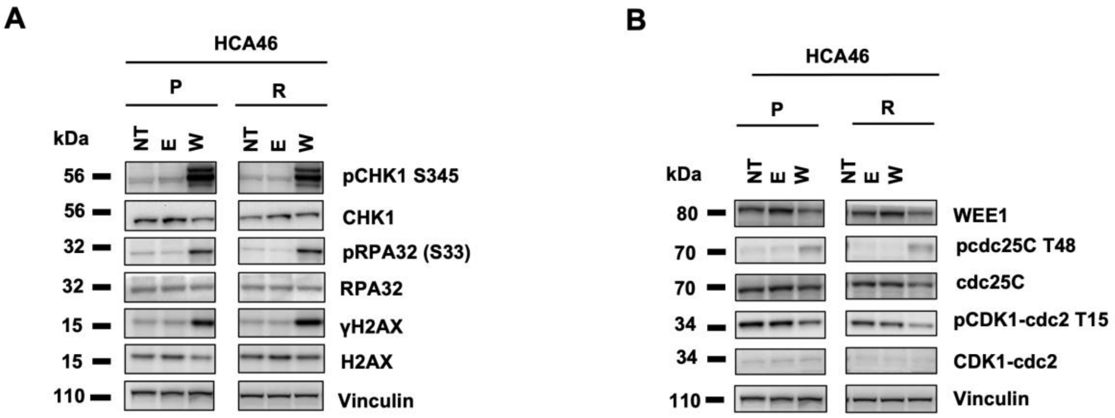

Rebuttal figure 3 (Figure EV10 in the revised manuscript) WEE1 inhibition induces replication stress and DNA damage signalling in parental and resistant HCA46 cells. *Immunoblot analysis in parental (P) and resistant (R) HCA46 cells seeded at* 5 × 10^5 cells per well in 6-well plates *and treated the next day with EGFRi-cetuximab (*25 µg/ml, 24h*) or W-WEE1i-adavosertib (300nM, 24h).(A) Immunoblot analysis of replication stress and DNA damage response markers (B) immunoblot analysis of WEE1 pathway players. Total protein levels are shown as controls, and vinculin was used as a loading control. Representative immunoblots from two independent biological experiments are shown.*

3. They also note an increase in RAD51 foci in resistant HT29 cells rather than the parental cells at baseline. However, RAD51 foci are significantly reduced following WEE1 inhibition in both resistant cell lines, suggesting that HR is downregulated in resisistant cells? However, it this is a bad comparison as these cells are treated for different periods of time -24H (for E, B+E or HU) compared to 8h (for WEE1i). This needs to be addressed. As it is misleading to have these in the same histogram. Also a timecourse should be performed comparing the impact of WEE1i over time in parental and resistant cell lines.

R. We thank the reviewer for raising this important point and we apologize for the unclear figure. We agree that RAD51 foci quantified after different treatments cannot be directly compared when treatment durations differ. In the present study, RAD51 foci following WEE1 inhibition were compared only to the untreated condition within the same cell line (parental or resistant), or between parental and resistant cells under the same treatment condition. However, in order to avoid any misleading or confusing comparisons, we have now generated new graphs separating 24h (E, B+E or HU) from 8h (W) treatment conditions rebuttal figure 4; these graphs are now part of Fig. 4 (panel C) and Fig. 7 (panel A) of the "clean" revised version.

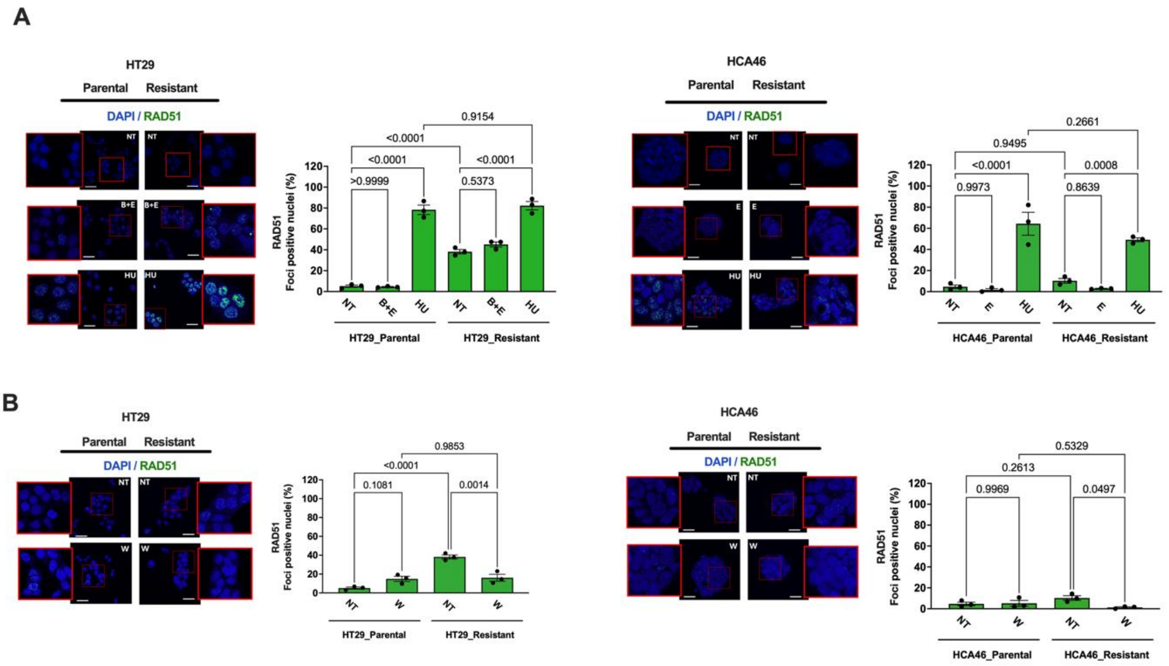

Rebuttal figure 4.(Fig. 4C and fig. 7A in the revised manuscript)

(A)RAD51 immunofluorescence analysis. Cells were seeded on coverslips and treated with targeted therapy (B+E): BRAFi-dabrafenib (4 µM, 24h) plus (EGFRi-cetuximab (5 µg/ml, 24h) for HT29 and E: EGFRi-cetuximab (25 µg/ml, 24h) for HCA46; HU: hydroxyurea (2.5 mM, 24 h) was used as a positive control for replication stress and DNA damage induction. (B) RAD51 foci formation in parental and resistant HT29 and HCA46 cells treated with W: WEE1i-adavosertib (300 nM, 8h). Nuclei were counterstained with DRAQ-5. Data are presented as mean ± SEM from n = 3 independent biological experiments (at least 5 independent fields were quantified, for a total of 100 nuclei/biological experiment). Images were acquired using a Leica Stellaris confocal microscope equipped with a 63× oil-immersion objective (NA 1.4) under identical acquisition settings. Scale bar, 25 µm. Statistical significance was assessed using one-way ANOVA with Tukey's multiple-comparisons test.

However, we would like to clarify that the use of distinct treatment durations reflects fundamental experimental constraints associated with WEE1 inhibition compared with hydroxyurea (HU) or other targeted agents. Indeed, we initially performed a time course experiment (8 and 24 hours), where also a 24 h WEE1 inhibition timepoint was tested to match the duration used for other treatments; however, this resulted in extensive cytotoxicity in resistant cells, including marked nuclear fragmentation and loss of cellular integrity. Under these conditions, γH2AX and RAD51 signals could not be reliably quantified, precluding meaningful interpretation of DDR dynamics in the comparison between the parental and resistant settings (see Rebuttal figure 5).

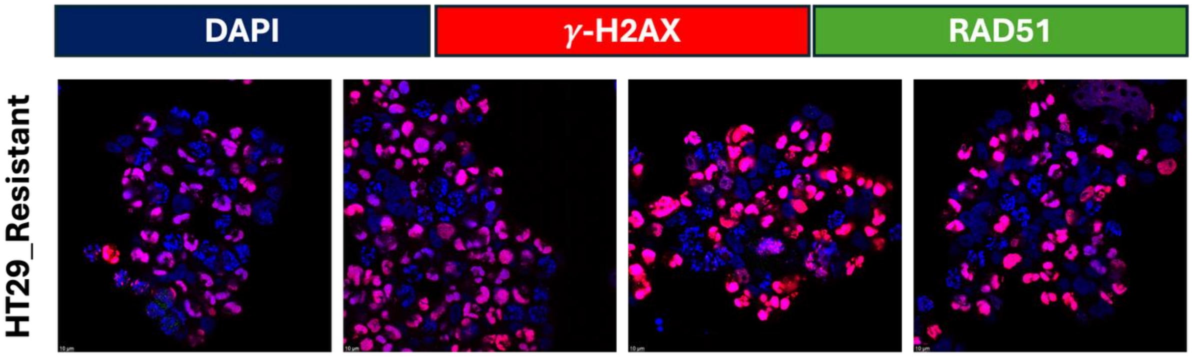

Rebuttal figure 5. A twenty-four-hour WEE1 inhibition causes marked nuclear fragmentation and loss of cellular integrity. *Four independent images of γH2AX and RAD51 co-staining in HT29 resistant cells are shown. Cells were seeded at 1 × 10^5 cells per coverslip and treated the following day with the WEE1 inhibitor adavosertib (300 nM, 24 h). Nuclei were stained with DAPI (blue), γH2AX foci are shown in red (appears as pan nuclear staining due to the high amount of damage), and RAD51 foci were stained in green (undetectable under these conditions).*

The 8 h WEE1 inhibition timepoint was therefore selected based on:
-prior studies using the same WEE1 inhibitor in replication stress and DNA repair analyses (1-Jennings L, Walters HA, McCraw TJ, et al. FBH1 deficiency sensitizes cells to WEE1 inhibition by promoting mitotic catastrophe. DNA Repair (Amst). 2024; 135: 103641.doi:10.1016/j.dnarep.2023.103641; 2- Dias MH, Friskes A, Wang S, et al. Paradoxical activation of oncogenic signaling as a cancer treatment strategy. Trends in Cancer. 2023; 9(12): 1016–1031. doi:10.1016/j.trecan.2023.09.003)
-our preliminary data of early replication-associated DNA damage responses,

Regarding RAD51 foci formation ability at the mechanistic level, WEE1 inhibition consistently resulted in a reduction in RAD51 foci in both parental and resistant cells, indicating impaired RAD51-mediated DNA repair. This observation is consistent with the established role of WEE1 in regulating replication-associated DNA damage (1-Krajewska M, Heijink AM, Bisselink YJ, et al. Forced activation of Cdk1 via Wee1 inhibition impairs homologous recombination. Nat Struct Mol Biol. 2013;20(7):856–863. doi:10.1038/nsmb.2590; 2- Kausar T, Li J, Lang J, et al. Sensitization of pancreatic cancer cells to gemcitabine by inhibition of WEE1 kinase correlates with impaired RAD51 focus formation. Cancer Biol Ther. 2015;16(5):598–607. doi:10.1080/15384047.2015.1026516; 3- Ghelli Luserna Di Rora A, et al. WEE1 inhibition induces DNA damage response, replication stress, and impairs homologous recombination in gynecological cancer models. Clin Cancer Res. 2020;26(24):6475–6487. doi:10.1158/1078-0432.CCR-20-1354 ; 4- Xu Y, Shen J, Ran X, et al. WEE1 inhibition targets homologous recombination proficiency and sensitizes to PARP inhibitors. Cancer Res. 2021;81(13):3410–3422. doi:10.1158/0008-5472.CAN-20-2811). The higher basal RAD51 foci levels observed in resistant cells likely reflect an increased burden of endogenous DNA damage (as demonstrated by increased levels of γH2AX) and greater reliance on DNA damage response pathways. Consequently, impairment of RAD51-dependent repair has disproportionately affected resistant cells.

We hope that this revision addresses the reviewer's concerns while accurately reflecting both the experimental constraints and the biological interpretation supported by the data.

4. The authors demonstrate robust synergy with WEE1i and SN-38. Could they and found a robust synergy of the two drugs. However, this is barely mentioned in the text and explanation of the results provided. Could the authors put further details in and perhaps discuss what they think is going on here?

R. We apologize with the referee if our text was not clear enough and we agree that the observed robust synergy between WEE1 inhibition (WEE1i) and SN-38 warranted a more detailed description and mechanistic discussion.

In the revised manuscript, we have now expanded both the ***Results*** *(line 325-338: "All combinations displayed synergistic activity in both parental and resistant cells, with synergy scores exceeding 10 (Fig. 8B and Fig. EV11). Notably, the combination of WEE1i with SN-38 emerged as the most synergistic and effective, particularly in resistant models compared with their parental counterparts (Synergy score >70 in resistant versus score <30 in parental cells). Mechanistically, SN-38 is known to stabilize Top1–DNA cleavage complexes, thereby generating replication-associated DNA lesions that promote replication fork stalling and collapse. Concomitant WEE1 inhibition abrogates checkpoint control and we propose that the combination pushes replication stress beyond a tolerable threshold, resulting in excessive accumulation of DNA damage and cell death. Collectively, these results provide a mechanistic rationale for the strong and consistent synergy observed between WEE1 inhibition and SN-38 and further support the translational potential of this combination as a strategy to overcome acquired resistance to EGFR-targeted therapies in CRC.") and* ***Discussion*** *(line 438-454: "Combination of sublethal WEE1i doses with DNA-damaging agents such as SN38 (the active metabolite of irinotecan), or with other DDRi such as ATMi or DNA-PKi, showed synergistic effects in cells and patient-derived models, with even greater efficacy in resistant tumors, supporting further exploration of rational combinations centred on WEE1 inhibition. This concept is consistent with the emerging paradigm that therapy-resistant cancers often harbor targetable non-oncogene dependencies, particularly in DNA damage response pathways. From a translational perspective, these findings are highly relevant. Irinotecan-based regimens remain a backbone of CRC treatment and the strong and reproducible synergy between WEE1 inhibition and SN-38, especially in EGFR inhibitor–resistant models, provides a compelling mechanistic rationale for clinical evaluation of this combination. Pharmacological inhibition of WEE1 overrides protective checkpoints, and, when combined with SN-38, these effects push replication stress beyond a tolerable threshold, leading to catastrophic DNA damage accumulation and cell death. Moreover, the preferential activity in resistant cells suggests the potential to selectively target tumors that have progressed on EGFR-targeted therapies, addressing a major unmet clinical need in metastatic CRC") sections to more thoroughly describe the magnitude of the combinatorial effect, including clarification of the synergy analyses performed and the consistency of the effect across models. We have better contextualize these findings within the framework of replication stress biology and provided a clearer mechanistic rationale for the strong synergy observed between WEE1i and SN-38.*

We thank the reviewer for prompting us to strengthen this aspect of the study.

Typos

- Lines 116–117: “the crucial role of WEE1 in ensuring e proper repair”

The phrase “WEE1 crucial role in ensuring e proper repair was substituted” was corrected to “the crucial role of WEE1 in ensuring the proper repair.” (see line 119 of the revised clean version)

- Line 223: “with respect to their parental counterpart”

The text was rephrased for better understanding. Revised text (line 219-220 clean version): “Comet assay analysis revealed elevated basal DNA damage (NT label) in the resistant models compared with their respective parental counterparts (Fig. 4A).”

- Line 250: “although at different extents”

“*At” was substituted with “to”, correcting the phrase “although to different extents*.”
(see line 284 of the revised clean version)

- Line 268: “effectors, such as RAD51”

“Such” was added to improve clarity.
Revised text (line 307 of the clean version): “as they both involve common effectors, such as RAD51.”

- Line 355: “might drives a resistance state”

The extra “s” was removed for grammar correction.
Revised text (line 391-392 of the clean version): “might drive a resistance state.”

- Line 381: “cancers including recurrent …”

“As” was replaced with “including.”
Revised text (line 421-422 of the clean version): “results in other types of cancers, including recurrent uterine serous cancer with TP53 mutations.”

- Line 403: “The identification of biomarkers predicting response to WEE1i remains an unmet need.”

The sentence “An unmet need remains the identification of biomarkers predicting response to WEE1i” was replaced with the reviewer-suggested wording.
(see line 461-462 of the revised clean version)

27th Mar 2026

Dear Prof. Arena,

Thank you for submitting your revised study. We have now received the referee's report, and as you will see below, they are satisfied with the revisions. I will therefore be able to accept your manuscript once the following minor editorial matters are addressed:

1/ Manuscript text:
- Please indicate any new modification in track changes mode.
- Please correct the following discrepancies in the authors' names: Giada De Lazzari (manuscript) vs Giada DeLazzari (submission system), Andrea Sartore-Bianchi (manuscript) vs Andrea Sartore Bianchi (submission system).
- Please correct the headings and order of the sections in the manuscript text to: Abstract / The Paper Explained / Introduction / Results / Discussion / Methods / Data Availability / Acknowledgements / Disclosure and Competing Interests Statement / References / Figure Legends / Expanded View Figure Legends.
- Remove the Statement of Significance and the list of suppl. materials from the manuscript text.
- "Material and Methods" should be renamed "Methods".
o Please indicate the origin of the cells.
o Please indicate the origin of the mice, as well as the housing and husbandry conditions. Please provide permit number for animal experiments.
o Human samples: Include a statement confirming that the experiments conformed to the principles set out in the WMA Declaration of Helsinki and the Department of Health and Human Services Belmont Report. If collected and within the bounds of privacy constraints report on age, sex and gender or ethnicity for all study participants.
o Statistics: Please provide statements on inclusion/exclusion criteria, sample size, as well as blinding and randomization.
- Data availability section: Thank you for providing a link to the Source Data. Please note that the datasets must be public before acceptance of the manuscript. Metabolomics data must be deposited.
- Funding should be merged with Acknowledgments. In the submission system, please move the funders from the comments section into the into the line-by-line list. The list of funders in our system will be linked in published articles to PubMed; it is therefore important that the list in our system is complete and accurate. The comments section will not be displayed in PubMed.
- Conflict of interest should be renamed to Disclosure and Competing Interests Statements.
- References: Please correct to alphabetical order and ten author names listed before et al; please remove the links.

2/ Figures:
- Figure EV1 spans 3 pages, please condense into one. If possible, we would also encourage you to merge several EV figures together to reduce the total number of EV figures.
- Please add the legend to the file of Table EV1.
- Please include the panel callouts for Fig. EV11.
- During our standard image analysis, we detected potential aberrations in the figure set. We kindly invite you to check the composition of Figure 4C, 6A, 6B, EV5 B and E, Figure EV10 A and B. Please provide an explanation if you introduce changes in these figure panels, and upload individual source data for the EV figure panels. Please note that figure reuse is allowed, but should be mentioned in the figure legends.
- Please address the queries from our data editors in the figure legends:
1. Please indicate the statistical test used for data analysis in the legends of figures 5G, H; EV7 A, B; EV12
2. Please note that the box plots need to be defined in terms of minima, maxima, centre, bounds of box and whiskers, and percentile in the legends of figures 5B, C, G, H; EV6
3. Please note that information related to n is missing in the legends of figures 5G, H; EV7 A, B; EV12
4. Please note that the error bars are not defined in the legends of figures EV7 A, B; EV 12

3/ Source Data:
Duplication were found in the Source Data for Figure 4A (see attached), please check and clarify.

4/ The "Draft Summary" should be renamed to "The Paper Explained" and added to the manuscript text.

5/ Thank you for providing the visual abstract. Please resize it to 550 x 300 pixels, remove the title and ensure that the text within the schematic remains legible.

6/ As part of the EMBO Publications transparent editorial process initiative (see our Editorial at http://embomolmed.embopress.org/content/2/9/329), EMBO Molecular Medicine will publish online a Review Process File (RPF) to accompany accepted manuscripts.
This file will be published in conjunction with your paper and will include the anonymous referee reports, your point-by-point response and all pertinent correspondence relating to the manuscript. Let us know whether you agree with the publication of the

RPF and as here, if you want to remove or not any figures from it prior to publication.
Please note that the Authors checklist will be published at the end of the RPF.

I look forward to receiving your revised manuscript.

Yours sincerely,

Lise Roth

Lise Roth, PhD

Senior Editor

EMBO Molecular Medicine

***** Reviewer's comments *****

Referee #1 (Comments on Novelty/Model System for Author):

The manuscript addresses an impotant issue relating to drug resistance to KRASG12C inhibitors and anti-EGFR in colorectal cancers. Here they document the role for WEE1 inhibitors in tareting resistant cell line models both in vitro and in vivo. These findings together with mechanistic insights provide a robust and clinically relevant study.

Referee #1 (Remarks for Author):

The authors have responded to all of this referees comments and the manuscript is improved.

The authors addressed the remaining editorial issues.

17th Apr 2026

Dear Prof. Arena,

Thank you for addressing the last editorial concerns. We are pleased to inform you that your manuscript is accepted for publication and is now being sent to our publisher to be included in the next available issue of EMBO Molecular Medicine.

Your manuscript will be processed for publication by EMBO Press. It will be copy edited and you will receive page proofs prior to publication. Please note that you will be contacted by Springer Nature Author Services to complete licensing and payment information.

You may qualify for financial assistance for your publication charges - either via a Springer Nature fully open access agreement or an EMBO initiative. Check your eligibility: https://link.springer.com/journal/44321/how-to-publish-with-us

Should you be planning a Press Release on your article, please get in contact with embo_production@springernature.com as early as possible in order to coordinate publication and release dates.

If you have any questions, please do not hesitate to contact the Editorial Office. Thank you for your contribution to EMBO Molecular Medicine.

Yours sincerely,

Lise Roth

Lise Roth, Ph.D
Senior Editor
EMBO Molecular Medicine

>>> Please note that it is EMBO Molecular Medicine policy for the transcript of the editorial process (containing referee reports and your response letter) to be published as an online supplement to each paper. If you do NOT want this, you will need to inform the Editorial Office via email immediately. More information is available here: https://link.springer.com/partners/embo-press/editorial-policies#Peer%20review

EMBO Press Author Checklist

Corresponding Author Name: Sabrina Arena	
Journal Submitted to: EMBO Molecular Medicine	
Manuscript Number: EMM-2025-22719	

USEFUL LINKS FOR COMPLETING THIS FORM
The EMBO Journal - Author Guidelines
EMBO Reports - Author Guidelines
Molecular Systems Biology - Author Guidelines
EMBO Molecular Medicine - Author Guidelines

Reporting Checklist for Life Science Articles (updated January
This checklist is adapted from Materials Design Analysis Reporting (MDAR) Checklist for Authors. MDAR establishes a minimum set of requirements in transparent reporting in the life sciences (see Statement of Task: 10.31222/osf.io/9sm4x). Please follow the journal's guidelines in preparing your
Please note that a copy of this checklist will be published alongside your article.

Abridged guidelines for figures
1. Data
The data shown in figures should satisfy the following conditions:
- the data were obtained and processed according to the field's best practice and are presented to reflect the results of the experiments in an accurate and unbiased manner.
- ideally, figure panels should include only measurements that are directly comparable to each other and obtained with the same assay.
- plots include clearly labeled error bars for independent experiments and sample sizes. Unless justified, error bars should not be shown for technical
- if n<5, the individual data points from each experiment should be plotted. Any statistical test employed should be justified.
- Source Data should be included to report the data underlying figures according to the guidelines set out in the authorship guidelines on Data

2. Captions
Each figure caption should contain the following information, for each panel where they are relevant:
- a specification of the experimental system investigated (eg cell line, species name).
- the assay(s) and method(s) used to carry out the reported observations and measurements.
- an explicit mention of the biological and chemical entity(ies) that are being measured.
- an explicit mention of the biological and chemical entity(ies) that are altered/varied/perturbed in a controlled manner.
- the exact sample size (n) for each experimental group/condition, given as a number, not a range;
- a description of the sample collection allowing the reader to understand whether the samples represent technical or biological replicates (including how many animals, litters, cultures, etc.).
- a statement of how many times the experiment shown was independently replicated in the laboratory.
- definitions of statistical methods and measures:
  - common tests, such as t-test (please specify whether paired vs. unpaired), simple χ2 tests, Wilcoxon and Mann-Whitney tests, can be unambiguously identified by name only, but more complex techniques should be described in the methods section;
  - are tests one-sided or two-sided?
  - are there adjustments for multiple comparisons?
  - exact statistical test results, e.g., P values = x but not P values < x;
  - definition of 'center values' as median or average;
  - definition of error bars as s.d. or s.e.m.

Please complete ALL of the questions below.
Select "Not Applicable" only when the requested information is not relevant for your study.

Materials

Newly Created Materials	Information included in the manuscript?	In which section is the information available? (Reagents and Tools Table, Materials and Methods, Figures, Data Availability Section)
New materials and reagents need to be available; do any restrictions apply?	Not Applicable	

Antibodies	Information included in the manuscript?	In which section is the information available? (Reagents and Tools Table, Materials and Methods, Figures, Data Availability Section)
For **antibodies** provide the following information:<br>- Commercial antibodies: RRID (if possible) or supplier name, catalogue number and or/clone number<br>- Non-commercial: RRID or citation	Yes	Reagent and Tools Table, Materials and Methods

DNA and RNA sequences	Information included in the manuscript?	In which section is the information available? (Reagents and Tools Table, Materials and Methods, Figures, Data Availability Section)
**Short novel DNA or RNA including primers, probes**: provide the sequences.	Not Applicable	

Cell materials	Information included in the manuscript?	In which section is the information available? (Reagents and Tools Table, Materials and Methods, Figures, Data Availability Section)
**Cell lines:** Provide species information, strain. Provide accession number in repository **OR** supplier name, catalog number, clone number, and/**OR** RRID.	Yes	Reagent and Tools Table, Materials and Methods
**Primary cultures:** Provide species, strain, sex of origin, genetic modification status.	Yes	Data Availability Section
Report if the cell lines were recently **authenticated** (e.g., by STR profiling) and tested for mycoplasma contamination.	Yes	Material and Methods

Experimental animals	Information included in the manuscript?	In which section is the information available? (Reagents and Tools Table, Materials and Methods, Figures, Data Availability Section)
**Laboratory animals or Model organisms:** Provide species, strain, sex, age, genetic modification status. Provide accession number in repository **OR** supplier name, catalog number, clone number, **OR** RRID.	Yes	Data Availability Section
**Animal observed in or captured from the field:** Provide species, sex, and age where possible.	Not Applicable	
Please detail **housing and husbandry conditions**.	Yes	Data Availability Section

Plants and microbes	Information included in the manuscript?	In which section is the information available? (Reagents and Tools Table, Materials and Methods, Figures, Data Availability Section)
**Plants:** provide species and strain, ecotype and cultivar where relevant, unique accession number if available, and source (including location for collected wild specimens).	Not Applicable	
**Microbes:** provide species and strain, unique accession number if available, and source.	Not Applicable	

Human research participants	Information included in the manuscript?	In which section is the information available? (Reagents and Tools Table, Materials and Methods, Figures, Data Availability Section)
If collected and within the bounds of privacy constraints report on age, sex and gender or ethnicity for all study participants.	Not Applicable	

Core facilities	Information included in the manuscript?	In which section is the information available? (Reagents and Tools Table, Materials and Methods, Figures, Data Availability Section)
If your work benefited from core facilities, was their service mentioned in the acknowledgments section?	Yes	Materials and Methods

Design

Study protocol	Information included in the manuscript?	In which section is the information available? (Reagents and Tools Table, Materials and Methods, Figures, Data Availability Section)
If study protocol has been **pre-registered, provide DOI in the manuscript**. For clinical trials, provide the trial registration number **OR** cite DOI.	Not Applicable	
Report the **clinical trial registration number** (at ClinicalTrials.gov or equivalent), where applicable.	Not Applicable	

Laboratory protocol	Information included in the manuscript?	In which section is the information available? (Reagents and Tools Table, Materials and Methods, Figures, Data Availability Section)
Provide DOI OR other citation details if **external detailed step-by-step protocols** are available.	Not Applicable	

Experimental study design and statistics	Information included in the manuscript?	In which section is the information available? (Reagents and Tools Table, Materials and Methods, Figures, Data Availability Section)
Include a statement about **sample size** estimate even if no statistical methods were used.	Yes	Figures
Were any steps taken to minimize the effects of subjective bias when allocating animals/samples to treatment (e.g. **randomization procedure**)? If yes, have they been described?	Yes	Figures, Materials and Methods
Include a statement about **blinding** even if no blinding was done.	Not Applicable	
Describe **inclusion/exclusion criteria** if samples or animals were excluded from the analysis. Were the criteria pre-established?<br><br>If sample or data points were omitted from analysis, report if this was due to attrition or intentional exclusion and provide justification.	Yes	Figures
For every figure, are **statistical tests** justified as appropriate? Do the data meet the assumptions of the tests (e.g., normal distribution)? Describe any methods used to assess it. Is there an estimate of variation within each group of data? Is the variance similar between the groups that are being statistically compared?	Yes	Figures

Sample definition and in-laboratory replication	Information included in the manuscript?	In which section is the information available? (Reagents and Tools Table, Materials and Methods, Figures, Data Availability Section)
In the figure legends: state number of times the experiment was **replicated** in laboratory.	Yes	Figures
In the figure legends: define whether data describe **technical or biological replicates**.	Yes	Figures

Ethics

Ethics	Information included in the manuscript?	In which section is the information available? (Reagents and Tools Table, Materials and Methods, Figures, Data Availability Section)
Studies involving **human participants**: State details of **authority granting ethics approval** (IRB or equivalent committee(s), provide reference number for approval.	Yes	Materials and Methods
Studies involving **human participants**: Include a statement confirming that **informed consent** was obtained from all subjects and that the experiments conformed to the principles set out in the WMA Declaration of Helsinki and the Department of Health and Human Services Belmont Report.	Yes	Materials and Methods
Studies involving **human participants: For publication of patient photos**, include a statement confirming that consent to publish was obtained.	Not Applicable	
Studies involving experimental **animals**: State details of **authority granting ethics approval** (IRB or equivalent committee(s), provide reference number for approval. Include a statement of compliance with ethical regulations.	Yes	Materials and Methods
Studies involving **specimen and field samples:** State if relevant **permits** obtained, provide details of authority approving study; if none were required, explain why.	Not Applicable	

Dual Use Research of Concern (DURC)	Information included in the manuscript?	In which section is the information available? (Reagents and Tools Table, Materials and Methods, Figures, Data Availability Section)
Could your study fall under dual use research restrictions? Please check biosecurity documents and list of **select agents and toxins** (CDC): https://www.selectagents.gov/sat/list.htm	Not Applicable	
If you used a select agent, is the security level of the lab appropriate and reported in the manuscript?	Not Applicable	
If a study is subject to dual use research of concern regulations, is the name of the **authority granting approval and reference number** for the regulatory approval provided in the manuscript?	Not Applicable	

Reporting

The MDAR framework recommends adoption of discipline-specific guidelines, established and endorsed through community initiatives. Journals have their own policy about requiring specific guidelines and recommendations to complement MDAR.

Adherence to community standards	Information included in the manuscript?	In which section is the information available? (Reagents and Tools Table, Materials and Methods, Figures, Data Availability Section)
State if relevant guidelines or checklists (e.g., **ICMJE, MIBBI, ARRIVE, PRISMA**) have been followed or provided.	Not Applicable	
For **tumor marker prognostic studies,** we recommend that you follow the **REMARK** reporting guidelines (see link list at top right). See author guidelines, under 'Reporting Guidelines'. Please confirm you have followed these guidelines.	Not Applicable	
For **phase II and III randomized controlled trials**, please refer to the **CONSORT** flow diagram (see link list at top right) and submit the CONSORT checklist (see link list at top right) with your submission. See author guidelines, under 'Reporting Guidelines'. Please confirm you have submitted this list.	Not Applicable	

Data Availability

Data availability	Information included in the manuscript?	In which section is the information available? (Reagents and Tools Table, Materials and Methods, Figures, Data Availability Section)
Have **primary datasets** been deposited according to the journal's guidelines (see 'Data Deposition' section) and the respective accession numbers provided in the Data Availability Section?	Yes	Figures, Data Availability Section
Were **human clinical and genomic datasets** deposited in a public access-controlled repository in accordance to ethical obligations to the patients and to the applicable consent agreement?	Not Applicable	
Are **computational models** that are central and integral to a study available without restrictions in a machine-readable form? Were the relevant accession numbers or links provided?	Not Applicable	
If publicly available data were reused, provide the respective **data citations in the reference list**.	Not Applicable	
